# Integrating population genetics and species distribution modelling to guide conservation of the noble crayfish, *Astacus astacus,* in Croatia

**DOI:** 10.1038/s41598-022-06027-8

**Published:** 2022-02-07

**Authors:** Leona Lovrenčić, Martina Temunović, Riho Gross, Marin Grgurev, Ivana Maguire

**Affiliations:** 1 Faculty of Science, University of Zagreb, Rooseveltov trg 6, 10000 Zagreb, Croatia; 2 Faculty of Forestry and Wood Technology, University of Zagreb, Zagreb, Croatia; 3 Estonian University of Life Sciences, Tartu, Estonia

**Keywords:** Climate change, Population genetics, Conservation biology, Ecological modelling, Invasive species, Zoology

## Abstract

The noble crayfish, *Astacus astacus,* is an indigenous European freshwater species. Its populations show significant declines caused by anthropogenic pressure on its habitats, climate change and the spread of invasive species. Diminishing populations’ trends and loss of genetic diversity highlight the need for effective conservation that will ensure their long-term survival. We combined population genetics and species distribution modelling (SDM) to reveal the impact of climate change and invasive species on the noble crayfish, and to guide future conservation programs of current populations. Our study showed that Croatian populations of *A. astacus* harbour an important part of species genetic diversity and represent significant genetic reservoir at the European level. The SDM results predicted substantial reductions of suitable habitats for *A. astacus* by the 2070; only 13% of its current potential distribution is projected to remain stable under pessimistic Representative Concentration Pathway (RCP 8.5) emission scenario. Moreover, most of the populations with high genetic diversity are located in the areas predicted to become unsuitable, and consequently have a high probability of being lost in the future. Further, SDM results also indicated considerable decrease of future habitat suitability for invasive crayfish species in Croatia, suggesting that climate change poses a major threat to already endangered *A. astacus*. The obtained results help in the identification of populations and areas with the highest conservation value which should be given the highest priority for protection. In order to preserve present diversity in areas that are predicted as suitable, we propose assisted migration and repopulation approaches, for enhancing populations’ size and saving maximum genetic variability. The result of our research emphasizes once again the benefits of multidisciplinary approach in the modern biodiversity conservation.

## Introduction

One of the greatest challenges faced by humanity is the mitigation of rapid biodiversity loss, associated with negative anthropogenic activities^1^. Indigenous crayfish species are among the most threatened animal taxa in European freshwaters where they are experiencing substantial population declines across their entire distribution ranges^2,3^. Thus, the requirement for appropriate conservation actions and policies are urgently needed^4^.

The noble crayfish, *Astacus astacus,* is a keystone species of high ecological, economic, and cultural importance in Europe^5^. It is an indigenous European freshwater species whose gene pool and wide current distribution have been shaped by geo-climatic events (i.e. Pleistocene glaciations) and anthropogenic impacts (i.e. translocations, pollution, habitat degradation). In Croatia, *A. astacus* is recorded in all three biogeographical regions: Continental, Alpine and Mediterranean. It is naturally distributed in the waterbodies of the Black Sea drainage, with a few recorded populations in the Adriatic Sea drainage that are of anthropogenic origin^6^. Large-scale genetic analyses revealed that *A. astacus* encompasses several mitochondrial lineages that have separated and diversified during the Pleistocene glaciations in the western and southern Balkans^7–9^, as well as in the lower Danube basin^7^. Results of microsatellite analysis revealed a differentiation of northern European populations from central European populations, with the former exhibiting a lower genetic diversity^10^. Furthermore, Schrimpf et al.^7,11^ and Laggis et al.^8^ revealed that this species harbours the highest genetic diversity in south-eastern Europe, while, low genetic diversity was detected in central and northern Europe, resulting from founder effects due to postglacial re-colonization and frequent human translocations for economic reasons^7^. Climate change is also among the major pressures for a decline in *A. astacus* populations, and its recognised vulnerability^12^ prompted the protection afforded by international legislation (Annex III of the Bern Convention, Annex V of Habitat Directive (92/43/EEC). In Croatia, its conservation status in all three biogeographical regions is unfavourable-inadequate following EU Habitats Directive (details are provided at https://nature-art17.eionet.europa.eu/article17/); during the last decade, 55% of *A. astacus* populations extirpated^6^, demonstrating the inadequacy of the species conservation. Consequently, as decreasing population trends continued^13^, *A. astacus* is listed as vulnerable even though protected by national legislation (NN 80/13).

Apart from being threatened by climate change, habitat loss and deterioration, the introduction of non-indigenous crayfish species (NICS), and their pathogens, are of additional major concern for indigenous crayfish species decline in freshwater ecosystems across Europe^5^. The NICS displace indigenous crayfish species through transmission of diseases such as crayfish plague caused by the oomycete *Aphanomyces astaci*, which is listed among the World’s 100 Worst Invasive Alien Species^14^. Furthermore, NICS’ success is attributed to competitive exclusion, tolerance to poor water quality and altered habitat^5^. In addition, apart from direct competition with indigenous crayfish populations, NICS possess the ability to change food webs and entire ecosystems^5^. During the last two decades, three NICS have been recorded in Croatian freshwaters: the marbled crayfish (*Procambarus virginalis*), the spiny-cheek crayfish (*Faxonius limosus*) and the signal crayfish (*Pacifastacus leniusculus*)^6,15^. Since the presence of *P. virginalis* remains limited to one local population^16^, and *P. leniusculus* and *F. limosus* have more extensive ranges, they represent the most problematic NICS for *A. astacus* populations in Croatian freshwaters*. Faxonius limosus* was first recorded in the Nature Park Kopački rit (eastern part of Continental Croatia) in 2003 where it has spread naturally along the Danube River from Hungary^6^. Compared to previous data this species has significantly expanded its range and continues to successfully spread in Croatian freshwater ecosystems displacing native *A. astacus* and *Pontastacus leptodactylus*^6,15^. *Pacifastacus leniusculus* is one of the most successful crayfish invaders in Europe^2^. Indeed, Chucholl^3^ found *P. leniusculus* to be the greatest threat to indigenous crayfish species among six NICS, in south-western Germany. This species is the most widespread NICS in Croatia due to its high dispersal rate which is among the highest recorded rates in Europe^15^. It is distributed in the continental part of the country, particularly in the Mura and Drava Rivers^6^, as well as in the Korana River, where it was illegally introduced^15^. The Korana River and its tributaries are the hotspots of indigenous Croatian astacofauna diversity, namely, *A. astacus*, *Austropotamobius torrentium* and *P. leptodactylus*, which encompass various divergent lineages, are distributed in those freshwater ecosystems^6,17^. To guide best-practice conservation and management actions for *A. astacus* populations in Croatia, we aimed to identify areas of potential current and future habitat suitability overlap between the indigenous *A. astacus* and the two problematic NICS using Species Distribution Modelling (SDM) (details are provided below). Identifying such areas will enable perceiving locations where endangered *A. astacus* may overlap with its invasive competitors, currently or in the future, under different climate change scenarios. This information, combined with genetic data, is a crucial piece of information for selecting *A. astacus* populations with the highest priority for protection.

Recent substantial declines and local extinctions of indigenous European crayfish populations have highlighted the need for developing appropriate conservation programmes and policies^4^. Contemporary, conservation planning includes genetic screening and selection of potentially suitable habitats for long-term preservation^4^. Genetic variability within the species is essential for species survival by securing its evolutionary and adaptive potential which result in populations’ capability of responding to new environmental conditions^18^. Reintroductions and/or restocking, as an approach for conservation of endangered species, are frequently disputed^19,20^, but they are still emphasized as management strategies for the conservation of indigenous crayfish species^20,21^. For successful conservation, greater insight into genetic diversity and structure of endangered crayfish populations is needed^4^. Microsatellites are among the most popular and versatile genetic markers with wide applications in population genetics, conservation biology, and evolutionary biology^22^. Population genetics analyses using microsatellites have been used successfully in several studies of *A. astacus* by providing insights into patterns of contemporary genetic diversity and structure, gene flow, effective population sizes, evolutionary history, and fates of introductions^7–11,23,24^. Conservation of indigenous crayfish species does not only include the assessment of a population’s genetic diversity in order to obtain restocking material for restoring their populations, but also includes the selection of potentially suitable habitats/sites in the future. The translocation of crayfish for reintroduction or restocking has been attempted many times across Europe, but the low rate of this measure’s success showed the need to improve site selection^4^. Species distribution modelling, also known as ecological niche modelling, is increasingly suggested as part of conservation decision making by forecasting environmental suitability for an endangered species^25,26^. Species distribution models require georeferenced biodiversity observations (e.g., species occurrences) and geographic layers of environmental information (e.g., climate, land cover, soil attributes). This approach represents a useful tool for selecting suitable habitats for conservation actions, such as translocation, and selection of sites for protection, and at the same time taking into consideration impacts of invasive species and climate change on species and habitats^3,27–30^. Global climate changes impact the size and extent of areas that may potentially be inhabited by many species^31,32^. Moreover, climate change is causing distributional shifts of many species worldwide due to altering environmental conditions to which they are adapted^32^. Factors like increasing water temperatures and long-term droughts could, undoubtedly impact dispersal‐limited freshwater crayfish impairing their survival^33^, while favouring the future spread of warm-water adapted NICS^34^.

Maintaining genetic diversity in an indigenous species is a pivotal goal at the global (European) and local (Croatian) levels (EU-Biodiversity strategy for 2030). Therefore, the present study has combined population genetic analyses and SDM as a guide for the future conservation actions of *A. astacus* genetic diversity. To provide such a baseline for conservation programs, the aims of our study were:

1. To reveal genetic diversity and population structure of *A. astacus* from 17 localities in Croatia (Table 1), using mitochondrial DNA (mtDNA) and nuclear DNA (microsatellite) markers;
2. To assess potential suitable habitats for the current and future period under different climate change scenarios for endangered *A. astacus* as well as for two NICS (*P. leniusculus* and *F. limosus*), and to identify areas of their potential current and future distribution overlap in Croatia using SDM;
3. To combine genetic data from *A. astacus* with its potential future distribution areas, as well as with future potential distribution of both NICS in order to identify populations and areas of the highest conservation value and priority for protection.

**Table 1:** Summarizing results across 15 microsatellite loci of population genetic diversity of studied *A. astacus* populations (N number of specimens, P proportion of polymorphic loci, N_*A*_ average number of alleles/locus, A_R_ allelic richness, A_PR_ rarefied number of private alleles, H_E_ expected heterozygosity, H_O_ observed heterozygosity, F_IS_ inbreeding coefficient and P_HWE_ probability of deviation from Hardy–Weinberg equilibrium after Bonferroni adjustments (not significant (ns) or significant (*)), null alleles—loci showing null alleles. Reference populations from Gross et al. (2021): JAR, MAK, TOT, JAN, VUK.

Population	Abbr	N	P	N_A_	A_R_	A_PR_	H_E_	H_O_	F_IS_	P_HWE_	Null alleles
Motičnjak	MOT	21	1.00	3.73	3.33	2.70	0.580	0.562	0.032	ns	4_42
Breznica	BRE	14	1.00	3.67	3.20	2.10	0.541	0.495	0.087	*	4_35
Burgeti	BUR	19	1.00	3.33	2.94	0.15	0.450	0.453	-0.005	ns	4_42
Ilova	ILO	24	1.00	5.20	4.19	1.95	0.684	0.630	0.081	ns	4_17, 4_20
Otuča	OUT	9	1.00	3.47	3.41	1.50	0.573	0.511	0.114	ns	4_3
Bijela	BIJ	21	1.00	4.47	3.72	3.30	0.590	0.568	0.037	ns	4_42, 4_48
Glogovica	GLO	28	1.00	5.20	3.95	0.90	0.638	0.569	0.109	ns	4_2, 4_3
Kikovac	KIK	30	1.00	4.00	3.42	0.60	0.554	0.566	-0.021	ns	
Sloboština	SLO	27	0.93	4.07	3.32	2.70	0.565	0.455	0.198*	*	4_17, 4_37, 4_42, 4_32, 4_3, 4_35
Bednja	BED	30	1.00	6.00	4.18	2.25	0.624	0.582	0.069	ns	4_35, 4_3
Kutjevačka	KUT	16	1.00	4.93	4.23	3.30	0.674	0.586	0.133	*	4_37
Veličanka	VEL	30	1.00	5.27	4.07	3.90	0.600	0.515	0.145*	ns	4_38, 4_37, 4_3
Jaruga	JAR	23	0.93	3.47	3.05	2.10	0.562	0.577	-0.03	ns	
Maksimir	MAK	30	0.93	2.93	2.39	3.30	0.355	0.350	0.013	ns	4_3
Totovec	TOT	30	1.00	3.27	3.09	1.35	0.577	0.557	0.036	ns	4_42
Jankovac	JAN	30	1.00	4.13	3.26	0.30	0.557	0.529	0.051	*	4_32, 4_44
Vuka	VUK	31	0.87	2.67	2.33	0.15	0.404	0.411	-0.02	ns	

We expect that a combination of SDM and genetic data will provide the required information needed to develop conservation programs for endangered *A. astacus*. Genetic characterisation will help identifying populations that should be given the highest priority in conservation, and which can also serve as suitable donor populations for possible repopulation and reintroduction programs not only in Croatia, but also in other European countries. Furthermore, we will be able to define areas and habitats that will be under the greatest pressure from NICS and climate change, as well as potential ark sites for this species long-time survival.

## Results

### Phylogenetic assignment of studied populations using mtDNA sequencing

**Figure 1:**
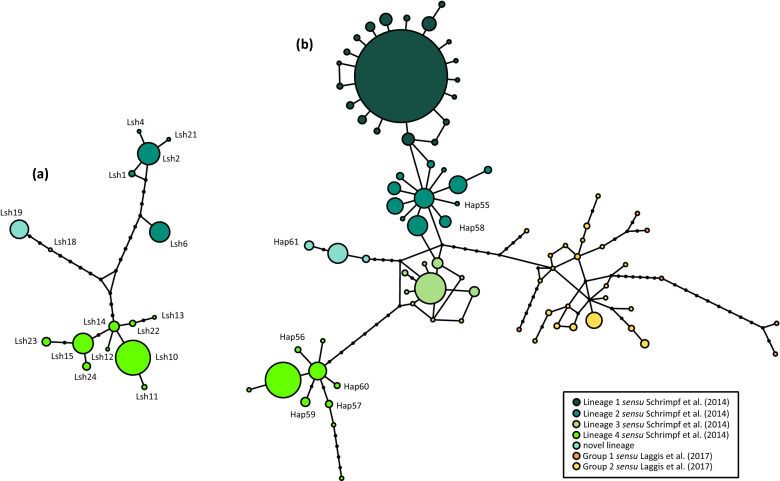
Median joining networks showing intraspecific phylogenetic relationships among **(a)** *COI* haplotypes from studied Croatian populations, and **(b)** concatenated *COI* + *16S* haplotypes from a European-wide dataset of *Astacus astacus,* with novel haplotypes labelled. Colours depict samples affiliation to mitochondrial lineages *sensu* Schrimpf et al.^7^ and groups *sensu* Laggis et al.^8^.

Intraspecific phylogenetic relationships and haplotype relatedness within *A. astacus* were described by the Median-joining (MJ) networks (Fig. 1). Reconstruction based on concatenated mtDNA data indicated the existence of six previously reported genetic lineages *undefined sensu* Schrimpf et al.^7^ and Laggis et al.^8^ within *A. astacus* in Europe (Fig. 1). Both *COI* and *16S* + *COI* MJ networks exhibited comparable results and based on the number of mutational steps could possibly indicate the presence of a new distinct lineage containing haplotypes from the two Croatian populations and several Slovenian populations (Lsh18/Hap51 and Lsh19/Hap61 in Fig. 1**,** Supplementary Table S1). Remaining novel concatenated haplotypes obtained from studied Croatian populations (Hap55-Hap60) were nested within formerly recognised mtDNA lineages. Precisely, haplotypes were recovered within two lineages, Lineages 2 and 4 *sensu* Schrimpf et al.^7^, with some populations harbouring crayfish with haplotypes from both lineages (populations JAN, MOT, OTU) (Fig. 1, Supplementary Table S1). The most widespread were populations belonging to Lineage 4 *sensu* Schrimpf et al.^7^ encompassing the whole *A. astacus* distribution range in Croatia, while Lineage 2 *sensu* Schrimpf et al.^7^ was found only in a few populations (Supplementary Fig. S1 and Supplementary Table S1).

### Population genetics

#### Genetic diversity

The final data set for the microsatellite analyses comprised 413 samples and 15 microsatellite loci; 269 successfully genotyped noble crayfish samples from 12 populations in this study, and reference data from five populations obtained in Gross et al.^9^. No evidence of linkage disequilibrium between pairs of loci tested over all populations was detected after Bonferroni correction (p = 0.0004), hence all microsatellites were considered as independent markers. Likewise, no signs for genotyping error due to stuttering or large allele dropout were observed as assessed by MICRO-CHECKER software. Null alleles were detected within several loci and populations (Table 1). Estimated null allele frequencies were mostly low, ranged from 0.00001 (several combinations) to 0.284 (4_37 in population VEL). Only two loci (4_37 and 4_42) exhibited a high null allele frequency, > 0.2, according to Chapuis and Estoup^35^. Even though the F_ST_ values increased slightly when recalculated using adjusted allele frequencies (Table 2), no significant differences were observed between uncorrected F_ST_ values and F_ST_ values corrected for null alleles (t-test = 0.44, p = 0.32). Furthermore, no locus showed null alleles across all populations and a bias on our evaluation of the population structure due to null alleles was very unlikely. Therefore, all subsequent analyses were conducted using the original data set.

**Table 2:** Pairwise uncorrected F_ST_ values (below) and F_ST_ values corrected for null alleles (above) from 15 microsatellite loci between all populations pairs (all values are statistically significant, p < 0.05; see Table 1 for populations’ abbreviation).

	MOT	BRE	BUR	ILO	OTU	BIJ	GLO	KIK	SLO	BED	KUT	VEL	JAR	MAK	TOT	JAN	VUK
MOT		0.292	0.305	0.207	0.228	0.271	0.224	0.294	0.229	0.147	0.228	0.209	0.240	0.462	0.267	0.272	0.422
BRE	0.296		0.371	0.227	0.254	0.278	0.228	0.339	0.278	0.285	0.202	0.227	0.287	0.500	0.307	0.346	0.381
BUR	0.311	0.377		0.274	0.309	0.313	0.235	0.288	0.334	0.255	0.251	0.286	0.204	0.550	0.382	0.336	0.478
ILO	0.212	0.241	0.281		0.191	0.146	0.160	0.207	0.171	0.188	0.157	0.169	0.222	0.392	0.245	0.247	0.331
OTU	0.230	0.262	0.316	0.196		0.245	0.222	0.288	0.182	0.202	0.121	0.171	0.239	0.447	0.267	0.290	0.426
BIJ	0.270	0.284	0.317	0.150	0.246		0.180	0.270	0.226	0.243	0.213	0.207	0.241	0.491	0.328	0.323	0.435
GLO	0.228	0.236	0.242	0.164	0.228	0.183		0.134	0.207	0.178	0.182	0.243	0.187	0.448	0.266	0.273	0.361
KIK	0.297	0.345	0.298	0.206	0.289	0.273	0.129		0.268	0.249	0.224	0.293	0.275	0.498	0.346	0.309	0.448
SLO	0.237	0.293	0.344	0.179	0.190	0.235	0.209	0.273		0.235	0.172	0.212	0.237	0.461	0.279	0.321	0.386
BED	0.149	0.286	0.261	0.195	0.206	0.243	0.184	0.251	0.241		0.221	0.234	0.203	0.408	0.217	0.154	0.409
KUT	0.233	0.216	0.255	0.167	0.116	0.218	0.192	0.226	0.188	0.225		0.130	0.239	0.440	0.266	0.260	0.358
VEL	0.213	0.242	0.291	0.177	0.180	0.213	0.250	0.296	0.222	0.236	0.147		0.256	0.438	0.268	0.291	0.377
JAR	0.239	0.293	0.205	0.228	0.246	0.241	0.188	0.277	0.240	0.204	0.245	0.259		0.506	0.312	0.310	0.439
MAK	0.464	0.506	0.556	0.393	0.446	0.492	0.450	0.496	0.457	0.406	0.441	0.440	0.509		0.361	0.462	0.538
TOT	0.278	0.321	0.394	0.259	0.278	0.338	0.276	0.356	0.293	0.227	0.281	0.284	0.321	0.378		0.276	0.383
JAN	0.284	0.360	0.349	0.258	0.300	0.331	0.287	0.318	0.336	0.160	0.274	0.303	0.320	0.469	0.289		0.438
VUK	0.424	0.389	0.486	0.332	0.428	0.435	0.365	0.449	0.386	0.411	0.364	0.382	0.442	0.539	0.392	0.448	

The summary statistics of the genetic diversity indices, for each population across 15 microsatellite loci, is shown in Table 1. All 15 microsatellite loci were polymorphic across all studied populations (Table 1). A total of 175 alleles were observed across the 15 microsatellite loci with an average of 12 alleles per locus, ranging from 5 alleles at locus 4_19 to 22 at locus 4_17. The mean number of alleles across loci ranged from 2.67 (VUK) to 6.00 (BED), mean allelic richness from 2.33 (VUK) to 4.23 (KUT), and H_E_ from 0.355 (MAK) to 0.684 (ILO) (Table 1). Total number of private alleles was 32. Rarefied number of private alleles ranged from 0.15 to 3.90, with the highest number observed in populations VEL, BIJ, KUT and MAK. The lowest number of private alleles was indicated for populations BUR, VUK, JAN and KIK (Table 1).

The H_O_ among populations ranged from 0.350 (MAK) to 0.630 (ILO), and the H_E_ from 0.355 (MAK) to 0.684 (ILO). H_E_ and H_O_ averaged to 0.560 and 0.524, respectively. The inbreeding coefficient per population was low to moderate and statistically not significant in the majority of populations (F_IS_ = -0.027 up to 0.198), so the intra-population variability was still evident. Only populations SLO and VEL exhibited statistically significant higher values of F_IS_ (0.198 and 0.145, respectively) indicating homozygote excess/heterozygote deficit. Significant deviations from Hardy–Weinberg equilibrium (HWE) were observed in four populations (BRE, SLO, KUT, JAN). Deviations from HWE were accompanied by positive F_IS_ values, indicating the heterozygote deficit and homozygote excess. Also in those populations, null alleles were detected (Table 1). Bottleneck analysis revealed consistent signs for recent contraction of population size in the populations JAR and TOT, which showed significant (p < 0.05) heterozygote excess according to the three mutational models tested (Supplementary Table S2).

#### Genetic differentiation and structure

The pairwise F_ST_ values ranged from 0.116 (between population KUT and OTU) to 0.556 (between MAK and BUR), with the global F_ST_ = 0.319 (Table 2). Populations MAK and VUK exhibited the highest F_ST_ values in comparison with other populations.

**Figure 2:**
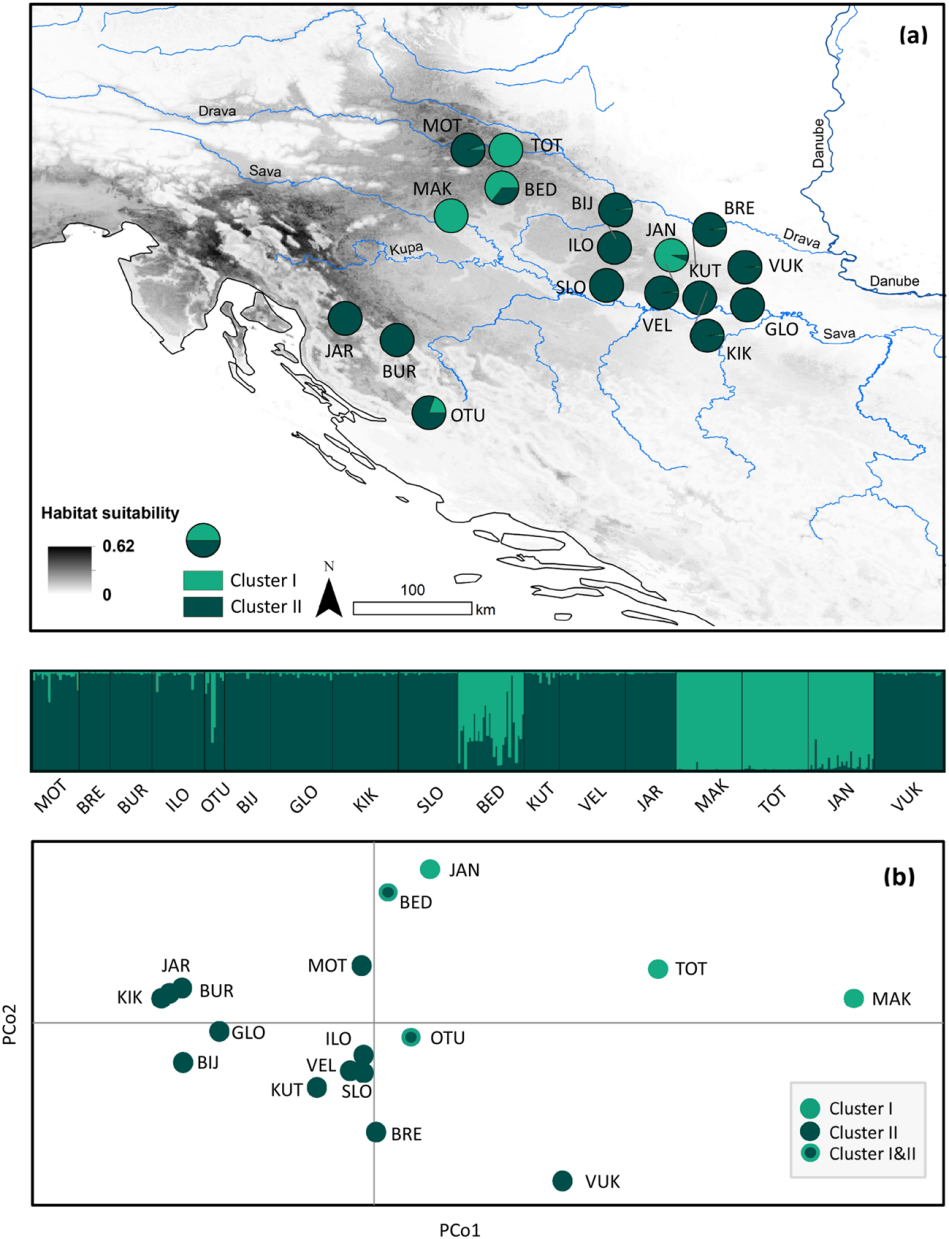
Genetic structure of the 17 studied *Astacus astacus* populations (see Table 1 for abbreviation) based on 15 microsatellites. **(a)** Genetic clustering inferred by STRUCTURE with the suggested K = 2 clusters. **(b)** Plots of the first two axes of a principal coordinates analysis (PCoA) based on Nei’ D_A_ genetic distances. Each dot represents one population with colours depicting genetic cluster identified in STRUCTURE. Grey shading in the map indicates projected future habitat suitability for *A. astacus* under RCP 8.5 scenario in 2070. Map was produced in ArcGIS 10.3 program package by authors of this study.

Population genetic structure was detected by the Bayesian clustering analysis implemented in the software STRUCTURE. The Bayesian Assignment Test was applied in order to assign individuals into clusters. The Evanno method, as implemented in STRUCTURE HARVESTER, revealed that the optimal number of clusters was two (ΔK = 2). Individuals were assigned to a certain cluster if their assignment probability was ≥ 0.8, where individuals with membership to a cluster below this threshold were considered to be admixed. Most individuals showed a high assignment to one genetic cluster. The cluster I included individuals from populations MAK, TOT, JAN and BED, whereas the cluster II comprised crayfish from populations MOT, BRE, BUR, ILO, BIJ, GLO, KIK, SLO, KUT, VEL, JAR, VUK and OTU (Fig. 2). In the populations OTU and BED evidence of admixture was observed in some individuals (Fig. 2). In addition, with the purpose of getting finer insight into genetic structure of *A. astacus*, we report the second most probable number of distinct genetic clusters, ΔK = 5: I) MOT, BED, JAN; II) BRE, VUK; III) BUR, GLO, KIK, JAR; IV) ILO, OTU, BIJ, SLO, KUT, VEL; and V) MAK, TOT (Supplementary Fig. S2).

Structure in the distribution of genetic variation was also depicted by the principal coordinates analysis (PCoA) (Fig. 2), where the PCo1 axis accounted for 25.94%, while the PCo2 axis accounted for 16.84% of the variation in the data. The PCoA revealed the existence of two well separated distinct clusters, with indication of another cluster between them. These results were congruent with the results of STRUCTURE (Fig. 2).

In order to reveal partitioning of genetic variance by AMOVA, populations were grouped according to their affiliation to the genetic clusters inferred by the Bayesian clustering analysis (Fig. 2). The results of the hierarchical genetic diversity analysis by AMOVA revealed that most of the genetic variation was represented among crayfish within populations (66.67% of variance) followed by variation among populations within clusters (27.01% of variance), while there was less variation between genetic clusters (6.31% of variance) (Supplementary Table S3).

### Species distribution models (SDMs)

#### Model performances

We evaluated model performance using area under the receiver operating characteristic curve (AUC)^36^. All SDMs for all species had excellent performance following interpretations for AUC values given in the literature^37,38^, with AUC > 0.9, regardless of the method used (Supplementary Table S4). The current ensemble model for *A. astacus* had an AUC value of 0.998, while for the NICS (*P. leniusculus* and *F. limosus*) AUC values were 0.999 for both species.

### Current and future Habitat suitability

**Figure 3:**
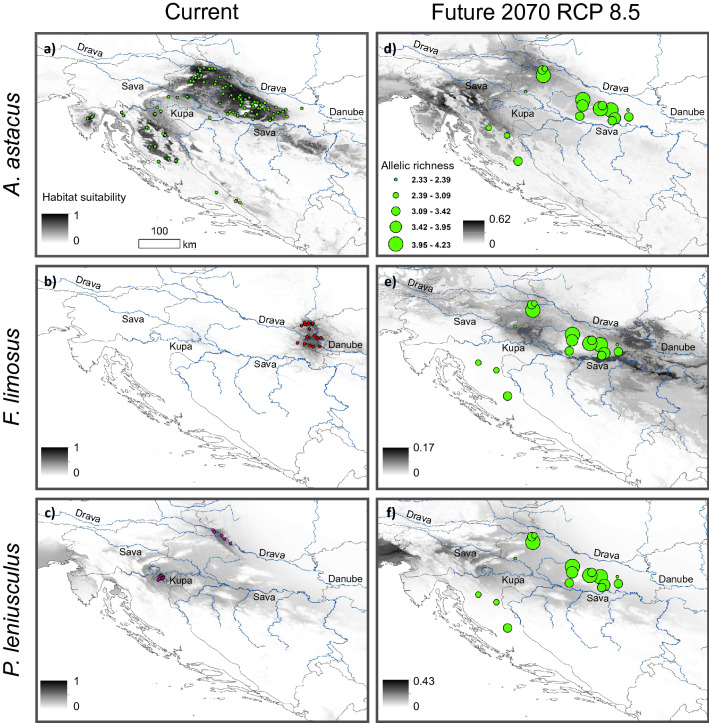
Ensemble potential habitat suitability for indigenous *Astacus astacus* and the two NICS, *Pacifastacus leniusculus* and *Faxonius limosus* in Croatia under current conditions **(a–c)** and future RCP 8.5 scenario in 2070 **(d–f)** based on SDMs. Occurrences of each species used for building SDMs are shown with coloured points **(a–c)**. Note that habitat suitability values in current projections are on the scale from 0 (unsuitable) to 1 (high suitability), while in the future projections habitat suitability values are on the scale from 0 (unsuitable) to maximum projected habitat suitability value. Future projections **(d–f)** of all species are shown in relation with the distribution of *A. astacus* allelic richness. Maps were produced in ArcGIS 10.3 program package by authors of this study.

Based on model projections under current environmental conditions, *A. astacus* habitat suitability values (ranging from 0, indicating areas of no or low suitability, to 1 indicating areas of the highest suitability) largely corresponded to current known distribution of this species in Croatia (Fig. 3a). Largest continuous suitable habitat for this species was projected into Continental Croatia, along and between the Drava and Sava Rivers, and along the Kupa River towards the south into Alpine Croatia, while smaller and more isolated areas of suitable habitat were predicted in the area of Mediterranean Croatia, where this species is not indigenous.

Current projections for NICS revealed highly suitable habitats for *F. limosus* in the easternmost part of Croatia corresponding to the regions along the Danube River and lower parts of the Sava River, and the small areas of suitable habitat were predicted along the middle part of the Sava River that could enable this species spreading towards the west of Croatia (Fig. 3b). For *P. leniusculus,* suitable habitats under current conditions were predicted in the Continental Croatia. The suitable habitats were anticipated along and between the Sava and Drava Rivers, as well as along the Kupa River, overlapping with habitats suitable for *A. astacus* (Fig. 3c).

Main trends in projected future habitat suitability under two considered RCP scenarios were similar for all species; therefore, we only report and show results for the more extreme RCP 8.5 pessimistic scenario (Fig. 3), while results for mid-range RCP 4.5 scenario are in the Supplement (Supplementary Fig. S3).

**Figure 4:**
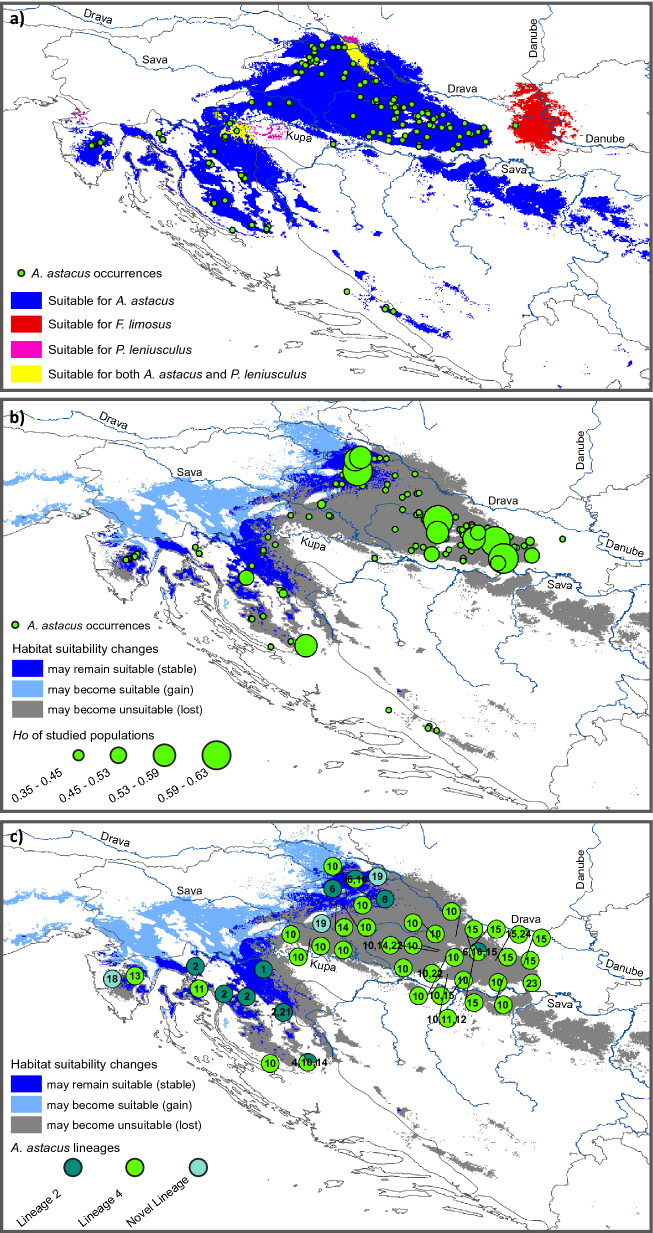
Potential overlap between suitable habitats for *Astacus astacus* and the two NICS. (**a**) Potential overlap between suitable habitats for *Astacus astacus* and the two NICS, *Pacifastacus leniusculus* and *Faxonius limosus* shown under current conditions**.** Projected changes between current and future habitat suitability for *A. astacus* under RCP 8.5 scenario in 2070 in relation to (**b**) observed heterozygosity (*H*_*o*_) and (**c**) *COI* haplotypes depicted by numbers and coloured according to mitochondrial lineages (both corresponding to Fig. 1). Known species occurrences are also shown in (**a,b**). Maps were produced in ArcGIS 10.3 program package by authors of this study.

Future projections for *A. astacus* suggest considerable negative impact of climate change on habitat suitability of this endangered species in Croatia (Fig. 3d). In particular, future climate change projections forecasted severe reduction in suitable habitat by 2070 in the easternmost parts of the distribution in Croatia (along and between the Sava and Drava Rivers) and to some (lesser) extent in the western part along the Kupa River towards the Alpine Croatia. In addition, future maximum habitat suitability values did not exceed 0.62, compared to current maximum of 0.98. Overall, potential future distribution of *A. astacus* was predicted to shift towards north-west with some gain of suitable habitat predicted in the area of Slovenia (Figs. 3 and 4). Ensemble model projections suggested that 87% of the current suitable habitat will be lost by 2070 under pessimistic RCP 8.5 scenario and only 13% will remain suitable (Fig. 4). Under mid-range RCP 4.5 scenario 65% of the current suitable habitat is projected to be lost and 35% remains stable.

Although the projected future suitable areas for NICS were wider compared to current ones, we found a severe decrease in habitat suitability values for both NICS under future climate predictions which were the most pronounced in *F. limosus* (Fig. 3e,f). Future maximum habitat suitability values did not exceed 0.43 for *P. leniusculus* and 0.17 for *F. limosus* (Fig. 3e,f). In most global circulation model (GCM) projections, maximum habitat suitability values were below the threshold maximizing the sum of sensitivity and specificity. Consequently, binary maps did not provide any suitable areas for NICS in the future, regardless of the RCP scenario. We therefore show and interpret only continuous future habitat suitability projections for NICS. Under future environmental conditions *F. limosus* is predicted to gain suitable habitats towards the west from its current distribution, along the Sava and Drava Rivers, although with very low probability, while suitable habitats for *P. leniusculus* are predicted to remain relatively similar to current ones, however with lower probability (Fig. 3e,f). Under current conditions we found an overlap between suitable habitats for *A. astacus* and *P. leniusculus* in the north-west Croatia along the Drava River and southern tributaries of the Sava River, which seem to be suitable for both species (Fig. 4a). Contrary, no overlap was detected between current suitable areas for *A. astacus* and *F. limosus* (Fig. 4a)*.*

Overlapping genetic variation of *A. astacus* with projected changes between its current and future habitat suitability indicated that majority of the areas harbouring highly diverse *A. astacus* populations are expected to become unsuitable in the future (Figs. 3d and 4b). The only populations (sampled for microsatellites) remaining in areas predicted to remain suitable in 2070 under RCP 8.5 scenario are MOT, BUR, BED, JAR and TOT (Fig. 4b). Considering mtDNA, overlap of *COI* haplotypes and future habitat suitability indicated that 50% (8/16) of *COI* haplotypes recorded in Croatia may be lost (namely, Lsh1, Lsh4, Lsh12, Lsh15, Lsh18, Lsh22, Lsh23 and Lsh24) (Fig. 4c). Majority of those haplotypes are distributed only in the eastern part of the Continental Croatia (Fig. 4c).

## Discussion

In our research, the fine scale phylogenetics, population genetics and species distribution modelling were used to explore genetic diversity and structure of *A. astacus*, as well as the impact of climate changes and invasive species on its populations. Analyses of genetic data coupled with species distribution models revealed the vulnerability of this keystone species to climate change.

The phylogenetic network based on mtDNA displayed intraspecific relationships within *A. astacus* consistent with the findings of previous studies^7–9^. Our results confirmed the existence of several genetic lineages, with the indication of novel divergent lineage containing haplotypes from Croatia and Slovenia (Hap51/Lsh18 and Hap61/Lsh19). Phylogenetic analysis indicates that all Croatian haplotypes belong to two mtDNA lineages (Lineages 2 and 4 *sensu* Schrimpf et al.^7^) that were also recorded in different countries across Europe. *Astacus astacus* exhibits lower mtDNA diversity and lower genetic structuring, without an obvious geographical pattern^7^, compared to other native European crayfish species^17,39–42^. Precisely, the MJ network showed weak phylogeographic structure and high haplotype-sharing even between geographically distant populations. This finding is consistent with the results of previous studies^9,11^ showing that the contemporary distribution and genetic structure of *A. astacus* were shaped through past geo-climatic events, strong anthropogenic influence on its habitat and frequent human mediated translocations that partly eroded their genetic structure. Similarities between distant *A. astacus* populations in several cases were explained by artificial stockings from different countries or populations^10,11^. Such a case was also observed in our study; crayfish from population JAR were used for aquaculture in the geographically distant hatchery Otočac, and consequently samples from both populations belonged to the same mtDNA lineage and shared the same haplotype (Lsh2). The genetic lineages of *A. astacus* diversified during the late Pliocene and throughout the Pleistocene, within the period between 1.7 and 0.5 mya^9^. Current *A. astacus* lineage distribution shows a divergence pattern congruent with the phenomena of insularity and isolation of multiple southern glacial refugia during repeated climatic pulses in the Pleistocene that produced a mosaic of lineages^43^.

Population genetic analyses on *A. astacus* across the sampled localities revealed high within-population genetic diversity and moderate differentiation among populations, that differed from the results of previous studies using the same^9^ or different microsatellite loci^7,8,10,11,24^. Overall, we detected a high number of alleles, proportion of polymorphic loci (P), allelic richness (A_R_) and observed heterozygosity (H_O_) in the study area. Genetic diversity, expressed as the P, A_R_ and H_O_ was higher in populations ILO, BED, KUT, VEL, while the level of genetic diversity was lower in populations MAK, VUK, BUR. Reduced genetic diversity in the populations MAK and BUR could be explained by the fact that they represent introduced populations^44,45^. Overall genetic diversity across the sampled localities of *A. astacus* was high when compared to the results of Gross et al.^10^, Schrimpf et al.^7,11^, Laggis et al.^8^ and Panicz et al.^24^ that used different set of microsatellite loci. A considerable number of private alleles was found in the majority of populations suggesting the presence of the unique genetic variation. Besides, private alleles are considered important in the long-term response to selection and the survival of populations and species^46^. We found that two populations (SLO and VEL), with significant homozygote excess, are vulnerable to inbreeding which may reduce the populations’ genetic diversity, and consequently lead to the loss of adaptive evolutionary potential of the species^47^. Furthermore, we analysed whether the recent bottleneck events influenced the observed genetic structure of the studied populations, and found that two populations did experience a recent bottleneck event (JAR and TOT). Bottlenecks in small remnant populations with limited gene flow could lead to low effective population sizes and cause fitness reductions across at least part of the species distribution. In Croatia, as elsewhere in Europe, *A. astacus* populations are mostly isolated by natural (i.e., watershed boundaries) or artificial (i.e., anthropologically influenced) barriers, and their distribution being frequently limited to small fragmented areas (geographical regions). Therefore, there is a reasonable concern that they may undergo significant declines in effective population size and that much of their genetic diversity might be lost.

The results of STRUCTURE and PCoA indicated the presence of genetic structuring among *A. astacus* populations in Croatia by identifying two main genetic clusters. Moreover, they revealed the presence of admixed individuals/populations assigned to different genetic cluster reflecting contributions of different ancestral groups or artificial translocation. Furthermore, results indicated to some extant populations’ structuring according to different river basins what is similar to that found by Gross et al.^9^. The pairwise F_ST_ values and AMOVA indicated moderate to high levels of genetic differentiation among studied populations demonstrating isolated populations with limited gene flow. The most genetically differentiated populations were MAK, TOT, JAN and VUK when compared to other studied populations. Contrary to other populations, the Vuka River (population VUK) flows directly into the Danube River which may explain the high F_ST_. Lower F_ST_ values obtained in this study reflected well geographical proximity, with the exception of populations OTU and KUT. The native range of *A. astacus* is restricted to the rivers of the Black Sea basin, whereas population OTU belongs to the Adriatic Sea basin. Thus, low F_ST_ value obtained for this population pair could indicate anthropogenic translocation between those two populations. Likewise, high values of F_ST_ for MAK populations could also be explained by artificial stockings from an unknown source. Moreover, it should be pointed out that MAK is recorded in an urban lake in Zagreb City, and that it may have been introduced from the Sava River where Karaman^48^ recorded *A. astacus*. Therefore, it is possible that this population represents a remnant astacofauna formerly present in the Sava River, with unique genetics that no longer exists elsewhere. This study discovered a higher value of global F_ST_ (= 0.319) compared to the study of *A. astacus* in central and northern Europe by Gross et al. (^10^; F_ST_ = 0.264) and Schrimpf et al. (^7^; F_ST_ = 0.232), but a lower value than that was found by Laggis et al. (^8^; F_ST_ = 0.400) and Gross et al. (^9^; F_ST_ = 0.512), in *A. astacus* populations in Greece and across the Balkan Peninsula, respectively. A pattern of isolated populations of freshwater species that contain high genetic diversity is characteristic for the Balkan Peninsula, that is recognised as one of the freshwater biodiversity hotspots^43,49,50^. Currently this region is characterised by fragmented and complex habitats with frequently no suitable surface water connections. Therefore, restricted dispersal and gene flow among populations probably led to genetic isolation of numerous freshwater species in this area, including crayfish. However, limited gene flow may lead to reduced effective population sizes, lower genetic diversity and increase the risk of local extinction, resulting in cascading effects through freshwater ecosystems^51^. Moreover, geographically isolated populations with low dispersal capabilities such as crayfish could experience problems in accommodating to ongoing climate changes due to limited possibilities for migration and a shift in their distribution toward more climate-suitable habitats.

Sensitivity to climate change in freshwater taxa was proved to be higher than in terrestrial taxa^52^, and vulnerability of freshwater crayfish to climate change, as well as to NICS has been demonstrated in many studies^3,30,33^. To evaluate the impact of climate change and NICS on the endangered *A. astacus*, we performed SDM. The models were able to capture the known ranges of *A. astacus* and two NICS in Croatia. Our predictions are concordant with previous studies of *A. astacus* distribution in Croatia^6^; majority of areas currently suitable for *A. astacus* are located in the area of Continental Croatia, including parts of Alpine Croatia and small isolated areas in Mediterranean Croatia where species was introduced^6^. Likewise, current projections for NICS, *F. limosus* and *P. leniusculus,* revealed highly suitable habitats corresponding to their present distribution in Croatia, but also encompassing areas for their potential spread. Our current projections suggested overlap between suitable habitats for *A. astacus* and *P*. *leniusculus*, a competitor which negatively affects *A. astacus* populations in the rivers of the continental part of Croatia through competitive exclusion and *A. astaci* transmission^5,15^. On the contrary, modelling the current potential distribution of *F. limosus* in Croatia did not detect any overlap between current suitable areas with *A. astacus,* as expected, since waterbodies of eastern Croatia are inhabited by *P. leptodactylus*^6^.

Overall, our future projections demonstrated that climate change may have major negative effects on the distribution of *A. astacus* by reducing the surface of climate-suitable areas available for this native European species. This result is in line with findings for other endangered aquatic species in Europe^54^. Change in thermal and precipitation regimes caused by global warming will probably lead to drastic range contractions of *A. astacus*. Consequently, this could drive population declines across the species distribution range in Croatia. This conclusion supports the alarming studies of Capinha et al.^34^ and Hossain et al.^52^ that predicted extreme loss of habitat suitability for freshwater crayfish due to climate change. Thus, our results indicate that climate change‐driven habitat loss represents a greater threat to *A. astacus* than the potential future distribution of the two studied NICS. A similar scenario was found for *Austropotamobius pallipes* in relation to the invasive *P. leniusculus* (^30,53,54^, see below).

Future SDM projections suggested that the suitable habitat for *A. astacus* will likely shift towards the north-west and practically disappear from the easternmost parts of Croatia due to the severe reduction (87% of currently suitable habitats) in habitat suitability by 2070. Furthermore, the most suitable areas for *A. astacus* in the future were forecasted to be in the western Croatian waterbodies, some of which are at high altitudes where *Austropotamobius torrentium* is currently recorded^6^. Even though the Alpine region and its freshwater ecosystems represent suitable habitats for most crayfish species^53^, these two indigenous species might compete for habitats and resources^29^. To overcome this, potential ark sites for *A. astacus* should be placed in the rivers and artificial lakes at lower altitudes in the Alpine region, as well as within gravel pits and oxbows alongside the Drava and Sava Rivers in the north-western part of Continental Croatia that were predicted as suitable in the future. These lower altitude waterbodies of the Alpine region would provide suitable ark sites for the crayfish from mtDNA Lineage 2 and/or Genetic cluster II, while crayfish from mtDNA Lineage 4 and/or Genetic cluster I could find refugia within gravel pits along the Drava and Sava Rivers in the north-western part of Continental Croatia. Keeping in mind that those suitable habitats are inaccessible to *A. astacus* due to natural dispersal barriers*,* human interventions would be needed. Assisted migration (AM) as an adaptation strategy for mitigating the projected effects of climate change on species is widely proposed^20,21^, especially for those with a life history features that prevents them from migrating to suitable habitats. However, it is a controversial topic among conservation biologists, with numerous identified risks. Arguments against AM include: risk of translocated species becoming invasive with associated negative biological, ecosystem and socioeconomic effects; spread of diseases and pathogens that can be transferred into new host species; removing individuals from existing populations increases the extinction risks facing those source populations^19,55^. In order to overcome those arguments, careful planning encompassing risk assessments, cost–benefit analyses, conducting AMs on a small scale, with robust monitoring that would enable prompt corrective actions to be taken if needed, along with political and public promotion, could insure successful AM implementations^21^.

Regardless of the RCP scenarios, our binary projections did not forecast any suitable areas for NICS in the future which should be interpreted with caution due to (a) the small number of available occurrences for NICS in the Croatian waterbodies used for SDMs; (b) models that do not account for human-mediated dispersal of NICS^56^; (c) the naturalised climatic niches of NICS that can differ from their natives’ climatic niches^28^; and (d) underestimated potential range expansion in the future due to the known issue of non-equilibrium of NICS with the environment within the invaded range^57^. Nevertheless, our continuous future habitat suitability projections showed that, even though the projected future suitable areas for NICS were more extensive than the current ones, drastic decrease in habitat suitability values for both NICS were displayed under future climate predictions. Explicitly, potential areas where *A. astacus* would overlap and compete with NICS virtually disappeared by 2070 under both climate change scenarios of high-warming (RCP 8.5) and low-warming conditions (RCP 4.5). This result is consistent with the results of Préau et al.^30^ showing no overlap between future suitable areas for *A. pallipes* and *P. leniusculus* in France based on SDMs, despite substantial ecological niche overlap between the two species. Likewise, Gallardo & Aldridge^54^ found that both endangered *A. pallipes* and invasive *P. leniusculus* were predicted to be negatively affected by climate changes in Europe. However, the range contraction was predicted to be more dramatic for the invasive *P. leniusculus*, leading to decreased overlap and consequently competition between the two species in the future, particularly in our study area. A more recent study by Zhang et al.^58^ confirmed that invasive *P. leniusculus* may lose a substantial portion of suitable habitat in Europe by 2070 in response to climate change. Moreover, Capinha et al.^34^ studied the potential distribution of indigenous crayfish species and NICS in Europe and found that climate-suitable areas were predicted to decrease by nearly 70% for *A. astacus*, 42% for *P. leniusculus*, and 49% for *F. limosus* by 2080. However, their models predicted that overlap of suitable ranges for native European crayfishes and invasive crayfishes would increase in the future which is contrary to our results. This may be because south-eastern Europe seems to be less suitable for *P. leniusculus* under changing climatic conditions^54,58^. It is therefore crucial to continue the monitoring of NICS invasions in the future.

Estimated reduction in habitat suitability by the end of this century indicates potential loss of a significant portion of the *A. astacus* genetic variability, especially in the eastern part of Continental Croatia that may potentially lose populations with high and unique genetic diversity. Minimising such possible losses in the future requires viable *A. astacus* populations to be established and maintained in ark sites/climate change refugia. Our results exposed an alarming need to prioritise conservation planning and management that will support existing populations and potentially establish new ones in the areas of stable habitat suitability that are expected to sustain *A. astacus* into the future. Species responses to climate change will depend on their distribution shifts to accommodate climate changes, and/or rely on the adaptation based on the standing genetic variation. Keeping in mind low dispersal abilities and isolated populations, we argue that assisted migration and population mixing approaches will be probably needed in the future to enhance the size and genetic diversity of remnant populations in order to maintain the long-term survival of the species^30,34,59^. Based on our results, we propose several donor populations for future restocking and reintroduction strategies. Namely, populations ILO, KUT, VEL, BAČ and BIJ, contain high and unique genetic diversity both at the mitochondrial and nuclear level, but they are predicted to be lost due to unsuitable habitats in the future. Dispersal as a fundamental behavioural mechanism is of great importance for adaptation and species’ responses to rapidly changing climate^26^. Strong dispersal limitations, habitat discontinuities and limited gene flow have a major effect on the ability of crayfish populations to withstand climate changes. Thus, assisted migration in climate change refugia seems a logical solution for slowing down genetic diversity erosion, reducing genetic load and the detrimental consequences of inbreeding, but also allows variations in allele frequencies^60–62^.

The adoption of such approaches for conservation purposes has gained significant momentum over the last few decades; reintroduction of the *A. astacus* into restored waterbodies has become common practice, even though the genetic origin of stocking material has rarely been considered^10^. Thus, potential ark sites should represent areas that maintain the highest contemporary genetic diversity in the species and predicted climate-suitable habitats for the future. *Astacus astacus* relocation should be preceded with a careful assessment regarding potential negative consequences of assisted gene flow that can impact the success of relocated populations, particularly when populations exhibit local adaptation to factors other than climate^63,64^. Also, introgression between local and translocated populations could result in outbreeding depression^60,61^. Still, Bláha et al.^23^ found no significant decline in genetic diversity between the source and translocated *A. astacus* populations after introduction. Furthermore, their study showed that even though the source populations did not possess high genetic diversity, their distinctiveness still made them suitable for conservation purposes. In addition, it is critical that climatically suitable sites outside *A. astacus* historical range for conservation purposes should be free from diseases, such as crayfish plague caused by oomycete *Aphanomyces astaci*.

In conclusion, our results suggest that securing the future of *A. astacus* will require significant interventions. This paper provides a baseline to guide these actions. Specifically, SDM combined with population genetics provided essential guidance for conservation actions aimed at safeguarding endangered *A. astacus* in Croatia by revealing genetic structure and identifying sites most suitable for protection and sites where climate change constitutes a threat. In addition, our study corroborates SDM as a valuable tool for conservation planning of threatened crayfish species by identifying areas within a species' distribution that may be vulnerable and suitable areas for assisted migration as shown in studies on European crayfish^34,52^, *A. pallipes* complex^3,29,30,53,65^, and *A. torrentium*^3^.

## Material and methods

### Genetic diversity and population structure

#### Sampling and DNA extraction

**Figure 5:**
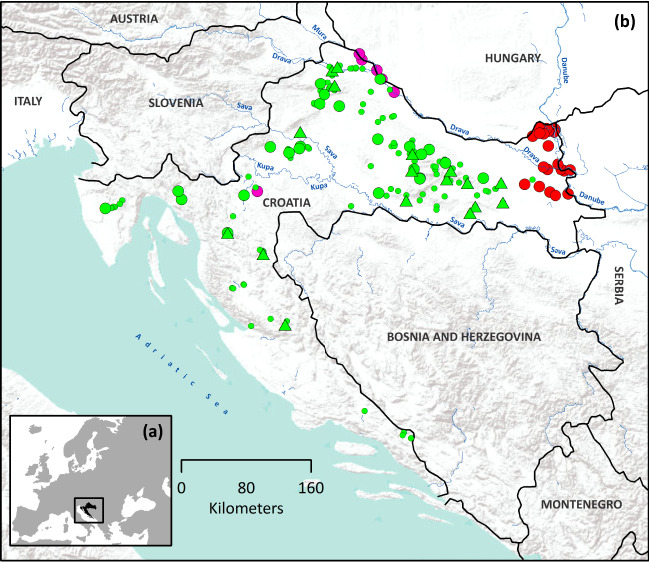
**(a)** Position of Croatia in Europe and **(b)** Geographical distribution of indigenous *Astacus astacus* and the two NICS, *Pacifastacus leniusculus* and *Faxonius limosus* in Croatia. Pink dots—*P. leniusculus*; red dots—*F. limosus*; small green dots—*A. astacus* occurrences; bigger green dots—*A. astacus* populations included into mtDNA analyses; green triangles—*A. astacus* populations included in both mtDNA and microsatellites analyses. Map was produced in ArcGIS 10.3 program package by authors of this study.

We collected *A. astacus* samples across its entire distribution range in Croatia (Supplementary Table S1, Fig. 5). Specimens were collected by hand or baited traps in accordance with ethical standards and with permissions of local authorities. One pereiopod from each individual was sampled and stored in 96% ethanol at 4 °C. Genomic DNA was extracted from the pereiopod’s muscle tissue using GenElute Mammalian Genomic DNA Miniprep kit (Sigma-Aldrich, St. Louis, MO) following the manufacturer’s protocol and stored at −20 °C.

#### Phylogenetic assignment of studied populations using mtDNA

Samples used for phylogenetic reconstruction are reported in Supplementary Table S1. Mitochondrial *16S* and *COI* genes were amplified and sequenced with universal primers 16Sar/16Sbr^66^ and LCO-1490/HCO-2198^67^ allowing comparison with previously published *A. astacus* sequences^7–9,68^. Polymerase chain reactions (PCR), purification and sequencing were performed according to Gross et al.^9^. Sequences were edited and aligned in Bioedit v. 7.2.5^69^. The final *COI* alignment did not contain any length variants or ambiguous sites and its final length was 623 bp, while *16S* alignment contained 1 length variation and its final length was 475 bp. In order to perform phylogenetic analysis comparable with previous studies of *A. astacus*, *COI* sequences (trimmed to 350 bp length) and *16S* sequences (475 bp length) from the same individual were concatenated (final alignment was 825 bp long). Sequences were collapsed to unique haplotypes using FaBox^70^, and all newly obtained haplotypes were submitted to GenBank (Supplementary Table S1).

Median-joining (MJ) network approach^71^ was used to visualise intraspecific relationships among haplotypes within *A. astacus* using PopArt^72^. Since *A. astacus* is characterised by low diversification in mtDNA^9^ and within-species data sets have fewer characters for phylogenetic analysis which diminish the statistical power of traditional phylogenetic methods^73^, we used phylogenetic networks that are better suited for description of intraspecific evolutionary relationships. Two MJ networks were reconstructed in order to determine non-hierarchical phylogenetic relationships between *A. astacus* haplotypes. Median-joining network I comprised *COI* sequences (623 bp long) from Croatian populations obtained in this study and in the study by Gross et al.^9^. Median joining network II included concatenated *16S* + *COI* sequences (825 bp long) obtained in this study and assembled with all available sequences at European level^7–9,11,68^. This approach enabled us to associate haplotypes obtained in the present study to the haplotypes obtained in previous research and indirectly to the lineages *sensu* Schrimpf et al.^7^ and groups *sensu* Laggis et al.^8^.

#### Population genetics of studied populations using microsatellites

For microsatellite analyses we amplified 19 species-specific tetranucleotide repeat microsatellite loci developed by Gross et al.^74^, and following modified protocols and procedures as in Gross et al.^9^. Microsatellite loci were genotyped on Applied Biosystems 3500 XL Genetic Analyser (Life Technologies, USA) using internal GeneScan 600 LIZ Size Standard v2.0 (Life Technologies, USA). Genotypes were scored using GeneMapper v.5 software (Life Technologies, USA), and were double-checked manually by two experts. Since four loci had overlapping allele size ranges (Aast4_26, Aast4_47, Aast4_10 and Aast4_30) they were omitted from further data analyses which were performed using 15 microsatellite loci. Also, several samples from different populations with more than two non-amplified loci were omitted from further analysis. Microsatellite loci were tested for potential presence of genotyping errors due to null alleles, stutter peaks and large allele dropout using MICRO-CHECKER v.2.2.3^75^. Pairwise linkage disequilibrium between all pairs of loci was tested using Fisher's exact test in GENEPOP v. 4.7.2^76^. Null allele frequencies based on the expectation–maximization (EM) algorithm^77^ and corrected F_ST_ values using the ENA method were estimated using FreeNA^35^ with a number of bootstrap replicates fixed to 10,000. The estimations of F_ST_, with and without null allele correction, were compared for each population using t-test in STATISTICA 13 (StatSoft. Inc).

Population genetics analyses were conducted with the microsatellite genotype data of 12 *A. astacus* populations obtained in this study that were supplemented with the microsatellite genotype data of five Croatian *A. astacus* populations (JAR, MAK, TOT, JAN and VUK) from the study by Gross et al.^9^ in order to enlarge data set and make analyses more robust.

#### Population genetic diversity

Population genetic diversity was assessed with standard descriptive statistics using GenAlEx v. 6.51^78^. Statistics included the percentage of polymorphic loci (P), mean number of alleles (N_A_), observed heterozygosity (H_O_) and unbiased expected heterozygosity (H_E_). Further, FSTAT v.2.9.4^79^ was used for estimation of allelic richness (A_R_) that was calculated as the number of alleles per locus independent of sample size, and the inbreeding coefficient (F_IS_).

The number of private alleles (A_PR_) was estimated by rarefaction method using HP Rare v.Feb-2-2009^80^ and multiplied by the number of used loci. Deviations from the Hardy–Weinberg equilibrium (HWE) for each population across all loci were tested using GENEPOP v. 4.7.2^76^. All probability tests were based on the Markov chain algorithm using 10,000 dememorization steps, 100 batches and 5000 iterations per batch. Significance levels were adjusted applying the Bonferroni correction to correct for the effect of multiple tests. Recent reductions in the effective population size using allele frequency data and potential signatures of recent bottlenecks were tested using the heterozygosity excess method implemented in BOTTLENECK v.1.2.02^81^ under three different mutational models: infinite allele model (IAM), stepwise mutation model (SMM) and two-phase model (TPM). Significant deviations from mutational-drift equilibrium were tested using the Wilcoxon sign rank test with 10,000 simulations.

#### Population genetic differentiation and structure

Genetic differentiation between all population pairs was estimated through pairwise F_ST_ values using FSTAT v.2.9.4^79^. Genetic structure among studied populations and assembling of individuals into groups (genetic clusters) was assessed using the Bayesian model-based clustering approach implemented in STRUCTURE v.2.3.4.^82^. The conditions performed were 10 runs for each genetic cluster (K) between 1 and 17 using a 100,000 burn-in period followed by 100,000 MCMC iterations, under the admixture model, with correlated allelic frequencies. The number of optimal K was inferred using the protocol defined by Evanno et al.^83^ as implemented in STRUCTURE HARVESTER v. 0.6.93^84^. STRUCTURE graphical results were plotted with CLUMPAK^85^. In addition, structure in the distribution of genetic variation was visualized by principal coordinates analysis (PCoA) using Nei's genetic distance in GenAlEx v. 6.51. Hierarchical analysis of molecular variance (AMOVA) was carried out using ARLEQUIN v. 3.5.1.2^86^ in order to estimate partitioning of genetic variance among groups, among populations within groups and within population. Populations were grouped according to their affiliation to the genetic clusters inferred from STRUCTURE; The cluster I included individuals from populations MAK, TOT, JAN and BED, and the cluster II comprised crayfish from populations MOT, BRE, BUR, ILO, BIJ, GLO, KIK, SLO, KUT, VEL, JAR, VUK and OTU (Fig. 2).

### Species distribution models (SDMs)

#### Species occurrence data

We compiled all known presence-only occurrences of *A. astacus* and the two NICS (*P. leniusculus* and *F. limosus*) from across Croatia from our own published and unpublished field sampling^6^. This resulted in a total of 174 occurrences for *A. astacus*, 22 for *F. limosus* and 17 for *P. leniusculus* (Fig. 3).

#### Environmental data

We initially considered 22 environmental variables from various sources and databases describing climate, topography and forest cover of the study area (Table 3). The 19 bioclimatic variables were obtained from the WorldClim 1.4 database^87^, altitude and slope were derived from a digital elevation model from the NASA Shuttle Radar Topography Mission (SRTM) elevation data (https://www2.jpl.nasa.gov/srtm), while the variable percentage of forest cover in 1 km^2^ was calculated from the Corine Land Cover 2018 dataset (https://land.copernicus.eu/pan-european/corine-land-cover). All environmental variables were used at a spatial resolution of ~ 1 km^2^. Predictor variables for SDMs of *A. astacus* and the two NICS were then selected based on our expert knowledge about their ecological relevance for the target species (potentially influencing species’ physiology and life history), excluding highly correlated ones based on variance inflation factor, VIF < 10 (usdm R package;^88^). Thus, the final predictor set for *A. astacus* included ten, and for NICS nine environmental variables (see Table 3).

**Table 3:** Environmental predictor variables used for building SDMs of indigenous *Astacus astacus* and the two NICS, *Pacifastacus leniusculus* and *Faxonius limosus*.

Variable ID	Variable description (unit)	*Astacus astacus*	NICS	Reference
bio2	Mean Diurnal Range (°C)	x	x	Hijmans et al., 2005^87^
bio4	Temperature Seasonality (SD × 100)	x	x	Hijmans et al., 2005^87^
bio5	Max Temperature of Warmest Month (°C)		x	Hijmans et al., 2005^87^
bio9	Mean Temperature of Driest Quarter (°C)	x		Hijmans et al., 2005^87^
bio14	Precipitation of Driest Month (mm)	x	x	Hijmans et al., 2005^87^
bio15	Precipitation Seasonality (CV)	x	x	Hijmans et al., 2005^87^
bio18	Precipitation of Warmest Quarter (mm)	x	x	Hijmans et al., 2005^87^
bio19	Precipitation of Coldest Quarter (mm)	x	x	Hijmans et al., 2005^87^
alt	Altitude (m)	x	x	https://www2.jpl.nasa.gov/srtm
slope	Slope derived from altitude (%)	x	x	https://www2.jpl.nasa.gov/srtm
forest_clc	Percentage of forest cover in 1km^2^ (%)	x		https://land.copernicus.eu/pan-european/corine-land-cover

x—used in model building.

#### Modelling procedure

To assess the potential current and future habitat suitability of *A. astacus* and two NICS (*P. leniusculus and F. limosus*), we developed SDMs using an ensemble approach implemented in R package BIOMOD2 ver. 3.3-7^89,90^. For each species we applied three different modelling methods (Random Forest—RF, Generalized Boosted Model—GBM and Maximum Entropy—Maxent) with ten replicates for each method (a total of 30 models for each species). Occurrences were combined with 10,000 random pseudo-absences drawn across the study area for methods that require absences^91^. In each run, 70% of the occurrences were used for model calibration, and the remaining 30% were used for model evaluation using AUC^36^.

To build the current ensemble model we used only highly reliable models with AUC > 0.9^37^ and obtained this ensemble as an AUC weighted average. The obtained current ensemble model was then projected under both current and future environmental conditions to obtain potential habitat suitability maps for each species. For future projections we used two RCP scenarios (mid-range emission scenario RCP 4.5 and pessimistic scenario RCP 8.5) and four global circulation models (GCMs) suitable for Europe^92^ (CCSM4, MIROC5, MPI-ESM-LR and HadGEM2-CC) for the 2070-time period (average for 2061–2080). Since future projections for variables slope, altitude and forest cover were not available, we kept them as constant in our future projections, assuming that they will not change for our study area during the considered time period. The available data on forest cover change in Croatia during the last decades and current forest management structure and practices provide confidence that at least for our study area, forest cover may remain stable in the future^93^ [https://forest.eea.europa.eu]. An ensemble future projection for each RCP scenario was obtained by taking an average of the four GCM projections. Multiple RCPs and GCMs were used to address the associated uncertainties arising from different climate change predictions^94^.

To obtain binary presence/absence maps helpful in model interpretation and for calculating changes in habitat suitability for *A. astacus*, we applied a threshold maximizing the sum of sensitivity and specificity^95^ to ensemble current and future continuous habitat suitability maps.

Finally, to estimate the effects of climate change on genetic diversity and structure of the focal species, we overlapped genetic data of *A. astacus* with its potential current and future suitable areas, as well as with future potential distribution of both NICS.

## Supplementary Information

Supplementary Table S1.

Supplementary Information.

Supplementary Legends.
