# Supplementary material for: Integrating population genetics and species distribution modelling to guide conservation of the noble crayfish, Astacus astacus, in Croatia
Source: Sci Rep. 2022 Feb 7;12:2040. doi: 10.1038/s41598-022-06027-8 (PMC8821615; doi:10.1038/s41598-022-06027-8)
Supplement: Supplementary file 1 — Supplementary Table S1. [file 41598_2022_6027_MOESM1_ESM.pdf]

Supplementary Table S1. Information on the *Astacus astacus* populations sampling sites (country and sampling site), *COI* haplotype codes (350 bp and 655 bp) with accession numbers, *16S* rRNA haplotype names with accession numbers and concatenated haplotype codes.

Country and sampling site	*COI* haplotype code (350 bp)	Acc. No. *COI* (350 bp)	*COI* haplotype code (623 bp)	Acc. No. *COI* (623 bp)	*16S* haplotype code (475 bp)	Acc. No. *16s* (475 bp)	Concatenated haplotype code
Albania: Devolli River			Lsh20/C32	KY682290			
Albania: Devolli River			Lsh20/C32	KY682290			
Albania: Devolli River			Lsh20/C32	KY682291			
Albania: Devolli River			Lsh20/C32	KY682291			
Albania: Lake Ohrid			Lsh17	KY682289			
Albania: Lake Prespa	AAS34_COI		Lsh17	MW726440	AAS19_16S	MW726238	Hap50
Albania: Lake Prespa	AAS34_COI		Lsh17	MW726439	AAS19_16S	MW726237	Hap50
Albania: Lake Prespa	AAS34_COI		Lsh17	MW726438			
Albania: Lake Prespa	AAS34_COI		Lsh17	MW726437	AAS19_16S	MW726236	Hap50
Albania: Lake Prespa	AAS34_COI		Lsh17	MW726436	AAS19_16S	MW726235	Hap50
Albania: Lake Prespa	AAS34_COI		Lsh17	MW726435	AAS19_16S	MW726234	Hap50
Albania: Lake Prespa	AAS34_COI		Lsh17	MW726434	AAS19_16S	MW726233	Hap50
Albania: Lake Prespa	AAS34_COI		Lsh17	MW726433			
Albania: Lake Prespa	AAS34_COI		Lsh17	MW726432			
Albania: Lake Prespa	AAS34_COI		Lsh17	MW726431			
Albania: Lake Prespa	AAS34_COI		Lsh17	MW726430			
Albania: Lake Prespa	AAS34_COI		Lsh17	MW726429	Aas19_16S	MW726232	Hap50
Albania: Lake Prespa	AAS34_COI		Lsh17	MW726428	Aas19_16S	MW726231	Hap50
Austria: Freundsheimer Weiher	Aas01_COI	KF888296			Aas01_16S	KF888279	Hap01
Austria: Freundsheimer Weiher	Aas01_COI	KF888296			Aas01_16S	KF888279	Hap01
Austria: Freundsheimer Weiher	Aas18_COI	KF888313			Aas08_16S	KF888286	Hap25
Belgium: Antoing	Aas01_COI	KF888296			Aas01_16S	KF888279	Hap01
Belgium: Antoing	Aas01_COI	KF888296			Aas01_16S	KF888279	Hap01
Belgium: Antoing	Aas01_COI	KF888296			Aas01_16S	KF888279	Hap01
Belgium: Antoing	Aas01_COI	KF888296			Aas01_16S	KF888279	Hap01
Belgium: Antoing	Aas01_COI	KF888296			Aas01_16S	KF888279	Hap01
Belgium: Antoing	Aas01_COI	KF888296			Aas01_16S	KF888279	Hap01
Belgium: Antoing	Aas01_COI	KF888296			Aas01_16S	KF888279	Hap01
Belgium: Antoing	Aas01_COI	KF888296			Aas01_16S	KF888279	Hap01
Belgium: Antoing	Aas01_COI	KF888296			Aas01_16S	KF888279	Hap01
Belgium: Antoing	Aas01_COI	KF888296			Aas01_16S	KF888279	Hap01
Belgium: Beloeil	Aas01_COI	KF888296			Aas01_16S	KF888279	Hap01
Belgium: Beloeil	Aas01_COI	KF888296			Aas01_16S	KF888279	Hap01
Belgium: Beloeil	Aas01_COI	KF888296			Aas01_16S	KF888279	Hap01
Belgium: Beloeil	Aas01_COI	KF888296			Aas01_16S	KF888279	Hap01
Belgium: Beloeil	Aas01_COI	KF888296			Aas01_16S	KF888279	Hap01
Belgium: Beloeil	Aas01_COI	KF888296			Aas01_16S	KF888279	Hap01
Belgium: Beloeil	Aas01_COI	KF888296			Aas01_16S	KF888279	Hap01
Belgium: Beloeil	Aas01_COI	KF888296			Aas01_16S	KF888279	Hap01
Belgium: Beloeil	Aas01_COI	KF888296			Aas01_16S	KF888279	Hap01
Belgium: Beloeil	Aas01_COI	KF888296			Aas01_16S	KF888279	Hap01
Belgium: Braine-le-Château	Aas01_COI	KF888296			Aas01_16S	KF888279	Hap01
Belgium: Braine-le-Château	Aas01_COI	KF888296			Aas01_16S	KF888279	Hap01
Belgium: Braine-le-Château	Aas01_COI	KF888296			Aas01_16S	KF888279	Hap01
Belgium: Braine-le-Château	Aas01_COI	KF888296			Aas01_16S	KF888279	Hap01
Belgium: Braine-le-Château	Aas01_COI	KF888296			Aas01_16S	KF888279	Hap01
Belgium: Braine-le-Château	Aas01_COI	KF888296			Aas01_16S	KF888279	Hap01
Belgium: Braine-le-Château	Aas01_COI	KF888296			Aas01_16S	KF888279	Hap01

Belgium: Braine-le-Château	Aas01_COI	KF888296		Aas01_16S	KF888279	Hap01
Belgium: Braine-le-Château	Aas01_COI	KF888296		Aas01_16S	KF888279	Hap01
Belgium: Braine-le-Château	Aas01_COI	KF888296		Aas01_16S	KF888279	Hap01
Belgium: Ciney	Aas01_COI	KF888296		Aas01_16S	KF888279	Hap01
Belgium: Ciney	Aas01_COI	KF888296		Aas01_16S	KF888279	Hap01
Belgium: Ciney	Aas01_COI	KF888296		Aas01_16S	KF888279	Hap01
Belgium: Ciney	Aas01_COI	KF888296		Aas01_16S	KF888279	Hap01
Belgium: Ciney	Aas01_COI	KF888296		Aas01_16S	KF888279	Hap01
Belgium: Ciney	Aas01_COI	KF888296		Aas01_16S	KF888279	Hap01
Belgium: Ciney	Aas01_COI	KF888296		Aas01_16S	KF888279	Hap01
Belgium: Ciney	Aas01_COI	KF888296		Aas01_16S	KF888279	Hap01
Belgium: Ciney	Aas01_COI	KF888296		Aas01_16S	KF888279	Hap01
Belgium: Ciney	Aas01_COI	KF888296		Aas01_16S	KF888279	Hap01
Belgium: Ecaussinnes	Aas01_COI	KF888296		Aas01_16S	KF888279	Hap01
Belgium: Ecaussinnes	Aas01_COI	KF888296		Aas01_16S	KF888279	Hap01
Belgium: Ecaussinnes	Aas01_COI	KF888296		Aas01_16S	KF888279	Hap01
Belgium: Ecaussinnes	Aas24_COI	KF888319		Aas01_16S	KF888279	Hap35
Belgium: Ecaussinnes	Aas24_COI	KF888319		Aas01_16S	KF888279	Hap35
Belgium: Ecaussinnes	Aas24_COI	KF888319		Aas01_16S	KF888279	Hap35
Belgium: Ecaussinnes	Aas42_COI			Aas01_16S	KF888279	Hap47
Belgium: Ecaussinnes	Aas42_COI			Aas01_16S	KF888279	Hap47
Belgium: Ecaussinnes	Aas43_COI			Aas01_16S	KF888279	Hap48
Belgium: Ecaussinnes	Aas43_COI			Aas01_16S	KF888279	Hap48
Belgium: Ecaussinnes	Aas01_COI	KF888296		Aas01_16S	KF888279	Hap01
Belgium: Ecaussinnes	Aas01_COI	KF888296		Aas01_16S	KF888279	Hap01
Belgium: Ecaussinnes	Aas01_COI	KF888296		Aas01_16S	KF888279	Hap01
Belgium: Ecaussinnes	Aas01_COI	KF888296		Aas01_16S	KF888279	Hap01
Belgium: Ecaussinnes	Aas01_COI	KF888296		Aas01_16S	KF888279	Hap01
Belgium: Ecaussinnes	Aas01_COI	KF888296		Aas01_16S	KF888279	Hap01
Belgium: Ecaussinnes	Aas01_COI	KF888296		Aas01_16S	KF888279	Hap01
Belgium: Ecaussinnes	Aas01_COI	KF888296		Aas01_16S	KF888279	Hap01
Belgium: Ecaussinnes	Aas24_COI	KF888319		Aas01_16S	KF888279	Hap35
Belgium: Ecaussinnes	Aas24_COI	KF888319		Aas01_16S	KF888279	Hap35
Belgium: Farm in Belgium	Aas01_COI	KF888296		Aas01_16S	KF888279	Hap01
Belgium: Farm in Belgium	Aas24_COI	KF888319		Aas01_16S	KF888279	Hap35
Belgium: Florenville	Aas01_COI	KF888296		Aas01_16S	KF888279	Hap01
Belgium: Florenville	Aas01_COI	KF888296		Aas01_16S	KF888279	Hap01
Belgium: Florenville	Aas01_COI	KF888296		Aas01_16S	KF888279	Hap01
Belgium: Florenville	Aas01_COI	KF888296		Aas01_16S	KF888279	Hap01
Belgium: Florenville	Aas01_COI	KF888296		Aas01_16S	KF888279	Hap01
Belgium: Fosses-la-Ville	Aas01_COI	KF888296		Aas01_16S	KF888279	Hap01
Belgium: Fosses-la-Ville	Aas01_COI	KF888296		Aas01_16S	KF888279	Hap01
Belgium: Fosses-la-Ville	Aas01_COI	KF888296		Aas01_16S	KF888279	Hap01
Belgium: Fosses-la-Ville	Aas01_COI	KF888296		Aas01_16S	KF888279	Hap01
Belgium: Fosses-la-Ville	Aas01_COI	KF888296		Aas01_16S	KF888279	Hap01
Belgium: Fosses-la-Ville	Aas01_COI	KF888296		Aas01_16S	KF888279	Hap01
Belgium: Fosses-la-Ville	Aas01_COI	KF888296		Aas01_16S	KF888279	Hap01
Belgium: Fosses-la-Ville	Aas01_COI	KF888296		Aas01_16S	KF888279	Hap01
Belgium: Fosses-la-Ville	Aas01_COI	KF888296		Aas01_16S	KF888279	Hap01
Belgium: Fosses-la-Ville	Aas01_COI	KF888296		Aas01_16S	KF888279	Hap01
Belgium: Gedinne	Aas01_COI	KF888296		Aas01_16S	KF888279	Hap01
Belgium: Gedinne	Aas01_COI	KF888296		Aas01_16S	KF888279	Hap01
Belgium: Gedinne	Aas01_COI	KF888296		Aas01_16S	KF888279	Hap01
Belgium: Gedinne	Aas01_COI	KF888296		Aas01_16S	KF888279	Hap01

Belgium: Gedinne	Aas01_COI	KF888296	Aas01_16S	KF888279	Hap01
Belgium: Gedinne	Aas01_COI	KF888296	Aas01_16S	KF888279	Hap01
Belgium: Gedinne	Aas01_COI	KF888296	Aas01_16S	KF888279	Hap01
Belgium: Gedinne	Aas01_COI	KF888296	Aas01_16S	KF888279	Hap01
Belgium: Gedinne	Aas01_COI	KF888296	Aas01_16S	KF888279	Hap01
Belgium: Gedinne	Aas01_COI	KF888296	Aas01_16S	KF888279	Hap01
Belgium: Gesves	Aas01_COI	KF888296	Aas01_16S	KF888279	Hap01
Belgium: Gesves	Aas01_COI	KF888296	Aas01_16S	KF888279	Hap01
Belgium: Gesves	Aas01_COI	KF888296	Aas01_16S	KF888279	Hap01
Belgium: Gesves	Aas01_COI	KF888296	Aas01_16S	KF888279	Hap01
Belgium: Gesves	Aas01_COI	KF888296	Aas01_16S	KF888279	Hap01
Belgium: Gesves	Aas01_COI	KF888296	Aas01_16S	KF888279	Hap01
Belgium: Gesves	Aas01_COI	KF888296	Aas01_16S	KF888279	Hap01
Belgium: Gesves	Aas01_COI	KF888296	Aas01_16S	KF888279	Hap01
Belgium: Gesves	Aas01_COI	KF888296	Aas01_16S	KF888279	Hap01
Belgium: Gesves	Aas01_COI	KF888296	Aas01_16S	KF888279	Hap01
Belgium: La Roche	Aas01_COI	KF888296	Aas01_16S	KF888279	Hap01
Belgium: La Roche	Aas01_COI	KF888296	Aas01_16S	KF888279	Hap01
Belgium: La Roche	Aas01_COI	KF888296	Aas01_16S	KF888279	Hap01
Belgium: La Roche	Aas01_COI	KF888296	Aas01_16S	KF888279	Hap01
Belgium: La Roche	Aas01_COI	KF888296	Aas01_16S	KF888279	Hap01
Belgium: La Roche	Aas01_COI	KF888296	Aas01_16S	KF888279	Hap01
Belgium: La Roche	Aas01_COI	KF888296	Aas01_16S	KF888279	Hap01
Belgium: La Roche	Aas01_COI	KF888296	Aas01_16S	KF888279	Hap01
Belgium: La Roche	Aas01_COI	KF888296	Aas01_16S	KF888279	Hap01
Belgium: La Roche	Aas01_COI	KF888296	Aas01_16S	KF888279	Hap01
Belgium: Libramont	Aas01_COI	KF888296	Aas01_16S	KF888279	Hap01
Belgium: Libramont	Aas01_COI	KF888296	Aas01_16S	KF888279	Hap01
Belgium: Libramont	Aas01_COI	KF888296	Aas04_16S	KF888282	Hap13
Belgium: Libramont	Aas01_COI	KF888296	Aas04_16S	KF888282	Hap13
Belgium: Lierneux	Aas01_COI	KF888296	Aas01_16S	KF888279	Hap01
Belgium: Lierneux	Aas01_COI	KF888296	Aas01_16S	KF888279	Hap01
Belgium: Lierneux	Aas01_COI	KF888296	Aas01_16S	KF888279	Hap01
Belgium: Lierneux	Aas01_COI	KF888296	Aas01_16S	KF888279	Hap01
Belgium: Lierneux	Aas01_COI	KF888296	Aas01_16S	KF888279	Hap01
Belgium: Lierneux	Aas01_COI	KF888296	Aas01_16S	KF888279	Hap01
Belgium: Lierneux	Aas01_COI	KF888296	Aas01_16S	KF888279	Hap01
Belgium: Lierneux	Aas01_COI	KF888296	Aas01_16S	KF888279	Hap01
Belgium: Lierneux	Aas01_COI	KF888296	Aas01_16S	KF888279	Hap01
Belgium: Lierneux	Aas01_COI	KF888296	Aas01_16S	KF888279	Hap01
Belgium: Marchin	Aas01_COI	KF888296	Aas01_16S	KF888279	Hap01
Belgium: Marchin	Aas01_COI	KF888296	Aas01_16S	KF888279	Hap01
Belgium: Marchin	Aas01_COI	KF888296	Aas01_16S	KF888279	Hap01
Belgium: Marchin	Aas01_COI	KF888296	Aas01_16S	KF888279	Hap01
Belgium: Marchin	Aas01_COI	KF888296	Aas01_16S	KF888279	Hap01
Belgium: Marchin	Aas01_COI	KF888296	Aas01_16S	KF888279	Hap01
Belgium: Marchin	Aas01_COI	KF888296	Aas01_16S	KF888279	Hap01
Belgium: Marchin	Aas01_COI	KF888296	Aas01_16S	KF888279	Hap01
Belgium: Marchin	Aas01_COI	KF888296	Aas01_16S	KF888279	Hap01
Belgium: Marchin	Aas01_COI	KF888296	Aas01_16S	KF888279	Hap01
Belgium: Momignies	Aas01_COI	KF888296	Aas01_16S	KF888279	Hap01
Belgium: Momignies	Aas01_COI	KF888296	Aas01_16S	KF888279	Hap01
Belgium: Momignies	Aas01_COI	KF888296	Aas01_16S	KF888279	Hap01
Belgium: Momignies	Aas01_COI	KF888296	Aas01_16S	KF888279	Hap01

Belgium: Momignies	Aas01_COI	KF888296	Aas01_16S	KF888279	Hap01
Belgium: Momignies	Aas01_COI	KF888296	Aas01_16S	KF888279	Hap01
Belgium: Momignies	Aas01_COI	KF888296	Aas01_16S	KF888279	Hap01
Belgium: Momignies	Aas01_COI	KF888296	Aas01_16S	KF888279	Hap01
Belgium: Momignies	Aas01_COI	KF888296	Aas01_16S	KF888279	Hap01
Belgium: Momignies	Aas01_COI	KF888296	Aas01_16S	KF888279	Hap01
Belgium: Ohey	Aas20_COI	KF888315	Aas13_16S	KF888291	Hap40
Belgium: Ohey	Aas20_COI	KF888315	Aas13_16S	KF888291	Hap40
Belgium: Ohey	Aas20_COI	KF888315	Aas13_16S	KF888291	Hap40
Belgium: Ohey	Aas20_COI	KF888315	Aas13_16S	KF888291	Hap40
Belgium: Ohey	Aas20_COI	KF888315	Aas13_16S	KF888291	Hap40
Belgium: Ohey	Aas20_COI	KF888315	Aas13_16S	KF888291	Hap40
Belgium: Ohey	Aas20_COI	KF888315	Aas13_16S	KF888291	Hap40
Belgium: Ohey	Aas20_COI	KF888315	Aas13_16S	KF888291	Hap40
Belgium: Ohey	Aas20_COI	KF888315	Aas13_16S	KF888291	Hap40
Belgium: Ohey	Aas20_COI	KF888315	Aas13_16S	KF888291	Hap40
Belgium: Ohey	Aas01_COI	KF888296	Aas01_16S	KF888279	Hap01
Belgium: Ohey	Aas01_COI	KF888296	Aas01_16S	KF888279	Hap01
Belgium: Ohey	Aas01_COI	KF888296	Aas01_16S	KF888279	Hap01
Belgium: Ohey	Aas01_COI	KF888296	Aas01_16S	KF888279	Hap01
Belgium: Ohey	Aas01_COI	KF888296	Aas01_16S	KF888279	Hap01
Belgium: Ohey	Aas01_COI	KF888296	Aas01_16S	KF888279	Hap01
Belgium: Ohey	Aas01_COI	KF888296	Aas01_16S	KF888279	Hap01
Belgium: Ohey	Aas01_COI	KF888296	Aas01_16S	KF888279	Hap01
Belgium: Ohey	Aas01_COI	KF888296	Aas01_16S	KF888279	Hap01
Belgium: Ohey	Aas01_COI	KF888296	Aas01_16S	KF888279	Hap01
Belgium: Paliseul	Aas01_COI	KF888296	Aas01_16S	KF888279	Hap01
Belgium: Paliseul	Aas01_COI	KF888296	Aas01_16S	KF888279	Hap01
Belgium: Paliseul	Aas01_COI	KF888296	Aas01_16S	KF888279	Hap01
Belgium: Paliseul	Aas01_COI	KF888296	Aas01_16S	KF888279	Hap01
Belgium: Paliseul	Aas01_COI	KF888296	Aas01_16S	KF888279	Hap01
Belgium: Paliseul	Aas01_COI	KF888296	Aas01_16S	KF888279	Hap01
Belgium: Paliseul	Aas01_COI	KF888296	Aas01_16S	KF888279	Hap01
Belgium: Paliseul	Aas01_COI	KF888296	Aas01_16S	KF888279	Hap01
Belgium: Paliseul	Aas01_COI	KF888296	Aas01_16S	KF888279	Hap01
Belgium: Paliseul	Aas01_COI	KF888296	Aas01_16S	KF888279	Hap01
Belgium: Sainte-Cécile	Aas01_COI	KF888296	Aas01_16S	KF888279	Hap01
Belgium: Sainte-Cécile	Aas01_COI	KF888296	Aas01_16S	KF888279	Hap01
Belgium: Sainte-Cécile	Aas01_COI	KF888296	Aas01_16S	KF888279	Hap01
Belgium: Sainte-Cécile	Aas01_COI	KF888296	Aas01_16S	KF888279	Hap01
Belgium: Sainte-Cécile	Aas01_COI	KF888296	Aas01_16S	KF888279	Hap01
Belgium: Sainte-Cécile	Aas01_COI	KF888296	Aas01_16S	KF888279	Hap01
Belgium: Sainte-Cécile	Aas01_COI	KF888296	Aas01_16S	KF888279	Hap01
Belgium: Sainte-Cécile	Aas01_COI	KF888296	Aas01_16S	KF888279	Hap01
Belgium: Sainte-Cécile	Aas01_COI	KF888296	Aas01_16S	KF888279	Hap01
Belgium: Sainte-Cécile	Aas24_COI	KF888319	Aas01_16S	KF888279	Hap35
Belgium: Tournai	Aas01_COI	KF888296	Aas01_16S	KF888279	Hap01
Belgium: Tournai	Aas01_COI	KF888296	Aas01_16S	KF888279	Hap01
Belgium: Tournai	Aas01_COI	KF888296	Aas01_16S	KF888279	Hap01
Belgium: Tournai	Aas01_COI	KF888296	Aas01_16S	KF888279	Hap01
Belgium: Tournai	Aas01_COI	KF888296	Aas01_16S	KF888279	Hap01
Belgium: Tournai	Aas01_COI	KF888296	Aas01_16S	KF888279	Hap01
Belgium: Tournai	Aas01_COI	KF888296	Aas01_16S	KF888279	Hap01
Belgium: Tournai	Aas01_COI	KF888296	Aas01_16S	KF888279	Hap01

Belgium: Tournai	Aas01_COI	KF888296			Aas01_16S	KF888279	Hap01
Belgium: Tournai	Aas01_COI	KF888296			Aas01_16S	KF888279	Hap01
Belgium: Tournai	Aas01_COI	KF888296			Aas01_16S	KF888279	Hap01
Belgium: Tournai	Aas01_COI	KF888296			Aas01_16S	KF888279	Hap01
Belgium: Tournai	Aas01_COI	KF888296			Aas01_16S	KF888279	Hap01
Belgium: Tournai	Aas01_COI	KF888296			Aas01_16S	KF888279	Hap01
Belgium: Tournai	Aas01_COI	KF888296			Aas01_16S	KF888279	Hap01
Belgium: Tournai	Aas01_COI	KF888296			Aas01_16S	KF888279	Hap01
Belgium: Tournai	Aas01_COI	KF888296			Aas01_16S	KF888279	Hap01
Belgium: Tournai	Aas01_COI	KF888296			Aas01_16S	KF888279	Hap01
Belgium: Tournai	Aas01_COI	KF888296			Aas01_16S	KF888279	Hap01
Belgium: Tournai	Aas24_COI	KF888319			Aas01_16S	KF888279	Hap35
Belgium: Walcourt	Aas01_COI	KF888296			Aas01_16S	KF888279	Hap01
Belgium: Walcourt	Aas01_COI	KF888296			Aas01_16S	KF888279	Hap01
Belgium: Walcourt	Aas01_COI	KF888296			Aas01_16S	KF888279	Hap01
Belgium: Walcourt	Aas01_COI	KF888296			Aas01_16S	KF888279	Hap01
Belgium: Walcourt	Aas01_COI	KF888296			Aas01_16S	KF888279	Hap01
Belgium: Walcourt	Aas01_COI	KF888296			Aas01_16S	KF888279	Hap01
Belgium: Walcourt	Aas01_COI	KF888296			Aas01_16S	KF888279	Hap01
Belgium: Walcourt	Aas01_COI	KF888296			Aas01_16S	KF888279	Hap01
Belgium: Walcourt	Aas01_COI	KF888296			Aas01_16S	KF888279	Hap01
Bulgaria: Beli Osam	Aas09_COI	KF888304			Aas01_16S	KF888279	Hap11
Bulgaria: Beli Osam	Aas09_COI	KF888304			Aas01_16S	KF888279	Hap11
Bulgaria: Beli Osam	Aas09_COI	KF888304			Aas01_16S	KF888279	Hap11
Bulgaria: Beli Osam	Aas09_COI	KF888304			Aas01_16S	KF888279	Hap11
Bulgaria: Gorna Trape	Aas01_COI	KF888296			Aas01_16S	KF888279	Hap01
Bulgaria: Gorna Trape	Aas09_COI	KF888304			Aas01_16S	KF888279	Hap11
Bulgaria: Gorna Trape	Aas09_COI	KF888304			Aas01_16S	KF888279	Hap11
Bulgaria: Gorna Trape	Aas09_COI	KF888304			Aas01_16S	KF888279	Hap11
Bulgaria: Gorna Trape	Aas09_COI	KF888304			Aas01_16S	KF888279	Hap11
Bulgaria: Gorna Trape	Aas09_COI	KF888304			Aas01_16S	KF888279	Hap11
Bulgaria: Gorna Trape	Aas13_COI	KF888308			Aas01_16S	KF888279	Hap17
Bulgaria: Gorna Trape	Aas13_COI	KF888308			Aas01_16S	KF888279	Hap17
Bulgaria: Razdvec	Aas01_COI	KF888296			Aas01_16S	KF888279	Hap01
Bulgaria: Razdvec	Aas01_COI	KF888296			Aas01_16S	KF888279	Hap01
Bulgaria: Razdvec	Aas01_COI	KF888296			Aas01_16S	KF888279	Hap01
Bulgaria: Razdvec	Aas01_COI	KF888296			Aas01_16S	KF888279	Hap01
Bulgaria: Razdvec	Aas10_COI	KF888305			Aas01_16S	KF888279	Hap12
Bulgaria: Razdvec	Aas11_COI	KF888306			Aas01_16S	KF888279	Hap14
Croatia: Bačica Creek, Rešetari	Aas26_COI	KF888321	Lsh10	MW726498	Aas15_16S	KF888293	Hap42
Croatia: Bačica Creek, Rešetari	Aas26_COI	KF888321	Lsh10	MW726496	Aas15_16S	KF888293	Hap42
Croatia: Bačica Creek, Rešetari	Aas26_COI	KF888321	Lsh11	MW726494	Aas15_16S	KF888293	Hap42
Croatia: Bačica Creek, Rešetari	Aas28_COI	KF888323	Lsh12	MW726495	Aas15_16S	KF888293	Hap44
Croatia: Bašnica River, Gračac	Aas27_COI	KF888322	Lsh14	MW726492	Aas15_16S	KF888293	Hap43
Croatia: Bašnica River, Gračac	Aas27_COI	KF888322	Lsh14	MW726491	Aas15_16S	KF888293	Hap43
Croatia: Bedenica	Aas26_COI		Lsh10	MZ456424	AAS15_16S	MZ467212	Hap42
Croatia: Bednja	Aas01_COI		Lsh6	MZ456482			
Croatia: Bednja	Aas01_COI		Lsh6	MZ456483	AAS10_16S	MZ467257	Hap58
Croatia: Bednja	Aas01_COI		Lsh6	MZ456484	AAS10_16S	MZ467258	Hap58
Croatia: Bednja	Aas01_COI		Lsh6	MZ456485	AAS10_16S	MZ467259	Hap58
Croatia: Bednja	Aas01_COI		Lsh6	MZ456486			
Croatia: Bednja	Aas01_COI		Lsh6	MZ456487			
Croatia: Bednja	Aas01_COI		Lsh6	MZ456488			
Croatia: Bednja	Aas01_COI		Lsh6	MZ456489	AAS10_16S	MZ467260	Hap58

Croatia: Bednja	Aas01_COI		Lsh6	MZ456490			
Croatia: Bednja	Aas01_COI		Lsh6	MZ456491	AAS10_16S	MZ467261	Hap58
Croatia: Bijela	Aas27_COI		Lsh14	MZ456385	AAS21_16S	MZ467166	Hap56
Croatia: Bijela	Aas26_COI		Lsh10	MZ456386	AAS15_16S	MZ467167	Hap42
Croatia: Bijela	AAS39_COI		Lsh22	MZ456387	AAS16_16S	MZ467168	Hap57
Croatia: Bijela	Aas26_COI		Lsh10	MZ456388	AAS15_16S	MZ467169	Hap42
Croatia: Bijela	Aas26_COI		Lsh10	MZ456389	AAS15_16S	MZ467170	Hap42
Croatia: Bijela	Aas26_COI		Lsh10	MZ456390	AAS15_16S	MZ467171	Hap42
Croatia: Bijela	AAS39_COI		Lsh22	MZ456391	AAS16_16S	MZ467172	Hap57
Croatia: Bijela	Aas26_COI		Lsh10	MZ456392	AAS15_16S	MZ467173	Hap42
Croatia: Bijela	Aas26_COI		Lsh10	MZ456393	AAS15_16S	MZ467174	Hap42
Croatia: Bijela	Aas26_COI		Lsh10	MZ456394	AAS15_16S	MZ467175	Hap42
Croatia: Bijela	Aas26_COI		Lsh10	MZ456395	AAS15_16S	MZ467176	Hap42
Croatia: Bijela	Aas26_COI		Lsh10	MZ456396	AAS15_16S	MZ467177	Hap42
Croatia: Bijela	Aas26_COI		Lsh10	MZ456397	AAS15_16S	MZ467178	Hap42
Croatia: Bijela	Aas27_COI		Lsh14	MZ456398	AAS21_16S	MZ467179	Hap56
Croatia: Bijela	Aas26_COI		Lsh10	MZ456399	AAS15_16S	MZ467180	Hap42
Croatia: Bijela	Aas26_COI		Lsh10	MZ456400	AAS15_16S	MZ467181	Hap42
Croatia: Bijela	Aas26_COI		Lsh10	MZ456476			
Croatia: Bijela	Aas26_COI		Lsh10	MZ456477	AAS15_16S	MZ467252	Hap42
Croatia: Bijela	Aas26_COI		Lsh10	MZ456478	AAS15_16S	MZ467253	Hap42
Croatia: Bijela	Aas26_COI		Lsh10	MZ456479	AAS15_16S	MZ467254	Hap42
Croatia: Bijela	Aas26_COI		Lsh10	MZ456480	AAS15_16S	MZ467255	Hap42
Croatia: Bijela	Aas26_COI		Lsh10	MZ456481	AAS15_16S	MZ467256	Hap42
Croatia: Braneška	Aas26_COI		Lsh10	MZ456415	AAS15_16S	MZ467201	Hap42
Croatia: Breznica	AAS40_COI		Lsh23	MZ456461	AAS15_16S	MZ467242	Hap59
Croatia: Breznica	AAS40_COI		Lsh23	MZ456462	AAS15_16S	MZ467243	Hap59
Croatia: Breznica	AAS40_COI		Lsh23	MZ456463			
Croatia: Breznica	AAS40_COI		Lsh23	MZ456464	AAS15_16S	MZ467244	Hap59
Croatia: Breznica	AAS40_COI		Lsh23	MZ456465	AAS15_16S	MZ467245	Hap59
Croatia: Breznica	AAS40_COI		Lsh23	MZ456466	AAS15_16S	MZ467246	Hap59
Croatia: Burgeti; Plitvice					AAS10_16S	MZ467139	
Croatia: Burgeti; Plitvice					AAS10_16S	MZ467140	
Croatia: Burgeti; Plitvice	Aas20_COI		Lsh21	MZ456361	AAS20_16S	MZ467141	Hap55
Croatia: Burgeti; Plitvice	Aas20_COI		Lsh2	MZ456362	AAS10_16S	MZ467142	Hap28
Croatia: Burgeti; Plitvice	Aas20_COI		Lsh2	MZ456363	AAS10_16S	MZ467143	Hap28
Croatia: Burgeti; Plitvice	Aas20_COI		Lsh2	MZ456364	AAS10_16S	MZ467144	Hap28
Croatia: Burgeti; Plitvice	Aas20_COI		Lsh2	MZ456365	AAS10_16S	MZ467145	Hap28
Croatia: Burgeti; Plitvice	Aas20_COI		Lsh2	MZ456366	AAS10_16S	MZ467146	Hap28
Croatia: Burgeti; Plitvice	Aas20_COI		Lsh2	MZ456367	AAS10_16S	MZ467147	Hap28
Croatia: Burgeti; Plitvice	Aas20_COI		Lsh2	MZ456368	AAS10_16S	MZ467148	Hap28
Croatia: Burgeti; Plitvice	Aas20_COI		Lsh2	MZ456369	AAS10_16S	MZ467149	Hap28
Croatia: Burgeti; Plitvice	Aas20_COI		Lsh2	MZ456370	AAS10_16S	MZ467150	Hap28
Croatia: Burgeti; Plitvice	Aas20_COI		Lsh2	MZ456371	AAS10_16S	MZ467151	Hap28
Croatia: Burgeti; Plitvice	Aas20_COI		Lsh2	MZ456372	AAS10_16S	MZ467152	Hap28
Croatia: Burgeti; Plitvice	Aas20_COI		Lsh2	MZ456373	AAS10_16S	MZ467153	Hap28
Croatia: Burgeti; Plitvice	Aas20_COI		Lsh2	MZ456374	AAS10_16S	MZ467154	Hap28
Croatia: Burgeti; Plitvice	Aas20_COI		Lsh2	MZ456375	AAS10_16S	MZ467155	Hap28
Croatia: Burgeti; Plitvice	Aas20_COI		Lsh2	MZ456376	AAS10_16S	MZ467156	Hap28
Croatia: Čemernica	Aas26_COI		Lsh10	MZ456421	AAS15_16S	MZ467209	Hap42
Croatia: Česma					AAS15_16S	MZ467208	
Croatia: Dubočanka			Lsh15	MW726477			
Croatia: Dubočanka	Aas27_COI	KF888322			Aas15_16S	KF888293	Hap43
Croatia: Glogovica	Aas26_COI		Lsh10	MZ456451	AAS15_16S	MZ467237	Hap42

Croatia: Glogovica	Aas26_COI		Lsh10	MZ456452			
Croatia: Glogovica	Aas26_COI		Lsh10	MZ456453			
Croatia: Glogovica	Aas26_COI		Lsh10	MZ456454			
Croatia: Glogovica	Aas26_COI		Lsh10	MZ456455			
Croatia: Glogovica	Aas26_COI		Lsh10	MZ456456	AAS15_16S	MZ467238	Hap42
Croatia: Glogovica	Aas26_COI		Lsh10	MZ456457	AAS15_16S	MZ467239	Hap42
Croatia: Glogovica	Aas26_COI		Lsh10	MZ456458	AAS15_16S	MZ467240	Hap42
Croatia: Glogovica	Aas26_COI		Lsh10	MZ456459	AAS15_16S	MZ467241	Hap42
Croatia: Glogovica	Aas26_COI		Lsh10	MZ456460			
Croatia: Ilova	Aas26_COI		Lsh10	MZ456445	AAS15_16S	MZ467232	Hap42
Croatia: Ilova	Aas26_COI		Lsh10	MZ456446			
Croatia: Ilova	Aas26_COI		Lsh10	MZ456492	AAS15_16S	MZ467262	Hap42
Croatia: Ilova	Aas26_COI		Lsh10	MZ456493	AAS15_16S	MZ467263	Hap42
Croatia: Ilova	Aas26_COI		Lsh10	MZ456494	AAS15_16S	MZ467264	Hap42
Croatia: Ilova	Aas26_COI		Lsh10	MZ456495	AAS15_16S	MZ467265	Hap42
Croatia: Ilova	Aas26_COI		Lsh10	MZ456496	AAS15_16S	MZ467266	Hap42
Croatia: Jankovac	Aas27_COI		Lsh15	MZ456444	AAS15_16S	MZ467231	Hap43
Croatia: Jankovac					AAS15_16S	MZ467282	
Croatia: Jankovac, Voćin	Aas26_COI		Lsh10	MW726476			
Croatia: Jankovac, Voćin	Aas01_COI		Lsh6	MW726592		MZ467131	Hap58
Croatia: Jankovac, Voćin	Aas01_COI		Lsh6	MW726591		MZ467132	Hap58
Croatia: Jankovac, Voćin	Aas01_COI		Lsh6	MW726590			
Croatia: Jankovac, Voćin	Aas01_COI		Lsh6	MW726589			
Croatia: Jankovac, Voćin	Aas01_COI		Lsh6	MW726588			
Croatia: Jankovac, Voćin	Aas01_COI		Lsh6	MW726587			
Croatia: Jankovac, Voćin	Aas01_COI		Lsh6	MW726586			
Croatia: Jankovac, Voćin	Aas01_COI		Lsh6	MW726585			
Croatia: Jankovac, Voćin	Aas01_COI		Lsh6	MW726584			
Croatia: Jankovac, Voćin	Aas01_COI		Lsh6	MW726583			
Croatia: Jankovac, Voćin	Aas26_COI		Lsh10	MW726470			
Croatia: Jankovac, Voćin	Aas26_COI		Lsh10	MW726469		MZ467133	Hap42
Croatia: Jankovac, Voćin	Aas26_COI		Lsh10	MW726468			
Croatia: Jankovac, Voćin	Aas26_COI		Lsh10	MW726467			
Croatia: Jaruga, Stajničko polje	Aas20_COI		Lsh2	MW726373			
Croatia: Jaruga, Stajničko polje	Aas20_COI		Lsh2	MW726372			
Croatia: Jaruga, Stajničko polje	Aas20_COI		Lsh2	MW726371			
Croatia: Jaruga, Stajničko polje	Aas20_COI		Lsh2	MW726370			
Croatia: Jaruga, Stajničko polje	Aas20_COI		Lsh2	MW726369			
Croatia: Jaruga, Stajničko polje	Aas20_COI		Lsh2	MW726368			
Croatia: Jaruga, Stajničko polje	Aas20_COI		Lsh2	MW726367			
Croatia: Jaruga, Stajničko polje	Aas20_COI		Lsh2	MW726366			
Croatia: Jaruga, Stajničko polje	Aas20_COI		Lsh2	MW726365			
Croatia: Jaruga, Stajničko polje	Aas20_COI		Lsh2	MW726364			
Croatia: Jaruga, Stajničko polje	Aas20_COI		Lsh2	MW726363			
Croatia: Jaruga, Stajničko polje	Aas20_COI		Lsh2	MW726362			
Croatia: Jaruga, Stajničko polje	Aas20_COI		Lsh2	MW726361			
Croatia: Jaruga, Stajničko polje	Aas20_COI		Lsh2	MW726360			
Croatia: Jaruga, Stajničko polje	Aas20_COI		Lsh2	MW726359			
Croatia: Jaruga, Stajničko polje	Aas20_COI		Lsh2	MW726358			
Croatia: Jaruga, Stajničko polje	Aas20_COI		Lsh2	MW726633	Aas10_16S	MW726334	Hap28
Croatia: Jaruga, Stajničko polje	Aas20_COI		Lsh2	MW726632	Aas10_16S	MW726333	Hap28
Croatia: Jaruga, Stajničko polje	Aas20_COI	KF888315			Aas10_16S	KF888288	Hap28
Croatia: Jaruga, Stajničko polje	Aas20_COI	KF888315			Aas10_16S	KF888288	Hap28
Croatia: Jarun	Aas26_COI		Lsh10	MZ456416	AAS15_16S	MZ467202	Hap42

Croatia: Kamešnica	Aas26_COI		Lsh10	MZ456418	AAS15_16S	MZ467204	Hap42
Croatia: Kamešnica	Aas26_COI		Lsh10	MZ456442	AAS15_16S	MZ467229	Hap42
Croatia: Kamešnica	Aas26_COI		Lsh10	MZ456443	AAS15_16S	MZ467230	Hap42
Croatia: Kikovac	Aas26_COI		Lsh10	MZ456467	AAS15_16S	MZ467247	Hap42
Croatia: Kikovac	Aas26_COI		Lsh10	MZ456468	AAS15_16S	MZ467248	Hap42
Croatia: Kikovac	Aas26_COI		Lsh10	MZ456469	AAS15_16S	MZ467249	Hap42
Croatia: Kikovac	Aas26_COI		Lsh10	MZ456470	AAS15_16S	MZ467250	Hap42
Croatia: Kikovac	Aas26_COI		Lsh10	MZ456471	AAS15_16S	MZ467251	Hap42
Croatia: Kikovac	Aas26_COI		Lsh10	MZ456472			
Croatia: Kikovac	Aas26_COI		Lsh10	MZ456473			
Croatia: Kikovac	Aas26_COI		Lsh10	MZ456474			
Croatia: Kikovac	Aas26_COI		Lsh10	MZ456475			
Croatia: Krapina	Aas27_COI	KF888322	Lsh14	MW726489	Aas15_16S	KF888293	Hap43
Croatia: Krivaja					AAS15_16S	MZ467205	
Croatia: Kutjevačka rijeka	AAS41_COI		Lsh24	MZ456516	AAS15_16S	MZ467276	Hap60
Croatia: Kutjevačka rijeka	AAS41_COI		Lsh24	MZ456517			
Croatia: Kutjevačka rijeka	AAS41_COI		Lsh24	MZ456518			
Croatia: Kutjevačka rijeka	Aas27_COI		Lsh15	MZ456519	AAS15_16S	MZ467277	Hap43
Croatia: Kutjevačka rijeka	Aas27_COI		Lsh15	MZ456520	AAS15_16S	MZ467278	Hap43
Croatia: Kutjevačka rijeka	AAS41_COI		Lsh24	MZ456521	AAS15_16S	MZ467279	Hap60
Croatia: Kutjevačka rijeka	AAS41_COI		Lsh24	MZ456522			
Croatia: Lake Bajer,	Aas26_COI		Lsh11	MZ456422	AAS15_16S	MZ467210	Hap42
Croatia: Lake Borovik	Aas27_COI	KF888322	Lsh15	MW726490	Aas15_16S	KF888293	Hap43
Croatia: Lake Ježevo, Velika Gorica	Aas26_COI	KF888321	Lsh10	MW726485	Aas15_16S	KF888293	Hap42
Croatia: Lake Maksimir	Aas38_COI		Lsh19	MW726388			
Croatia: Lake Maksimir	Aas38_COI		Lsh19	MW726387			
Croatia: Lake Maksimir	Aas38_COI		Lsh19	MW726386			
Croatia: Lake Maksimir	Aas38_COI		Lsh19	MW726385			
Croatia: Lake Maksimir	Aas38_COI		Lsh19	MW726384			
Croatia: Lake Maksimir	Aas38_COI		Lsh19	MW726383			
Croatia: Lake Maksimir	Aas38_COI		Lsh19	MW726382			
Croatia: Lake Maksimir	Aas38_COI		Lsh19	MW726381			
Croatia: Lake Maksimir	Aas38_COI		Lsh19	MW726380			
Croatia: Lake Maksimir	Aas38_COI		Lsh19	MW726379			
Croatia: Lake Maksimir	Aas38_COI		Lsh19	MW726378			
Croatia: Lake Maksimir	Aas38_COI		Lsh19	MW726377			
Croatia: Lake Maksimir	Aas38_COI		Lsh19	MW726376			
Croatia: Lake Maksimir	Aas38_COI		Lsh19	MW726375			
Croatia: Lake Maksimir	Aas38_COI		Lsh19	MW726374			
Croatia: Lake Motičnjak	Aas01_COI		Lsh6	MW726551			
Croatia: Lake Motičnjak	Aas01_COI		Lsh6	MW726550			
Croatia: Lake Motičnjak	Aas01_COI		Lsh6	MW726549			
Croatia: Lake Motičnjak	Aas01_COI		Lsh6	MW726548			
Croatia: Lake Motičnjak	Aas01_COI		Lsh6	MW726547			
Croatia: Lake Motičnjak	Aas01_COI		Lsh6	MW726546			
Croatia: Lake Motičnjak	Aas26_COI		Lsh10	MW726466			
Croatia: Lake Motičnjak	Aas26_COI		Lsh10	MW726465			
Croatia: Lake Motičnjak	Aas26_COI		Lsh10	MW726464			
Croatia: Lake Motičnjak	Aas26_COI		Lsh10	MW726463			
Croatia: Lake Motičnjak	Aas26_COI		Lsh10	MW726462			
Croatia: Lake Motičnjak	Aas26_COI		Lsh10	MW726461			
Croatia: Lake Motičnjak	Aas26_COI		Lsh10	MW726460			
Croatia: Lake Motičnjak	Aas26_COI		Lsh10	MW726459			
Croatia: Lake Motičnjak	Aas26_COI		Lsh10	MW726458			

Croatia: Lake Motičnjak	Aas26_COI		Lsh10	MW726457			
Croatia: Lake Totovec	Aas38_COI		Lsh19	MW726406	Aas17_16S	MZ467134	Hap61
Croatia: Lake Totovec	Aas38_COI		Lsh19	MW726405	Aas17_16S	MZ467135	Hap61
Croatia: Lake Totovec	Aas38_COI		Lsh19	MW726404	Aas17_16S	MZ467136	Hap61
Croatia: Lake Totovec	Aas38_COI		Lsh19	MW726403	Aas17_16S	MZ467137	Hap61
Croatia: Lake Totovec	Aas38_COI		Lsh19	MW726402			
Croatia: Lake Totovec	Aas38_COI		Lsh19	MW726401	Aas17_16S	MZ467138	Hap61
Croatia: Lake Totovec	Aas38_COI		Lsh19	MW726400			
Croatia: Lake Totovec	Aas38_COI		Lsh19	MW726399			
Croatia: Lake Totovec	Aas38_COI		Lsh19	MW726398			
Croatia: Lake Totovec	Aas38_COI		Lsh19	MW726397			
Croatia: Lake Totovec	Aas38_COI		Lsh19	MW726396			
Croatia: Lake Totovec	Aas38_COI		Lsh19	MW726395			
Croatia: Lake Totovec	Aas38_COI		Lsh19	MW726394			
Croatia: Lake Totovec	Aas38_COI		Lsh19	MW726393			
Croatia: Lake Vukovina	Aas26_COI	KF888321			Aas14_16S	KF888292	Hap41
Croatia: Lake Vukovina	Aas26_COI	KF888321			Aas14_16S	KF888292	Hap41
Croatia: Lake Vukovina	Aas26_COI	KF888321			Aas14_16S	KF888292	Hap41
Croatia: Lake Vukovina	Aas26_COI	KF888321			Aas14_16S	KF888292	Hap41
Croatia: Lake Vukovina	Aas26_COI	KF888321			Aas14_16S	KF888292	Hap41
Croatia: Lipnica	Aas26_COI		Lsh10	MZ456527	AAS15_16S	MZ467281	
Croatia: Ljubešćak	Aas01_COI		Lsh6	MZ456401	AAS10_16S	MZ467182	Hap58
Croatia: Ljubešćak	Aas01_COI		Lsh6	MZ456402	AAS10_16S	MZ467183	Hap58
Croatia: Ljubešćak	Aas01_COI		Lsh6	MZ456403	AAS10_16S	MZ467184	Hap58
Croatia: Ljubešćak	Aas01_COI		Lsh6	MZ456404	AAS10_16S	MZ467185	Hap58
Croatia: Lonja, Paka	Aas26_COI		Lsh10	MZ456425	AAS15_16S	MZ467213	Hap42
Croatia: Motičnjak	Aas26_COI		Lsh10	MZ456434	AAS15_16S	MZ467224	Hap42
Croatia: Motičnjak	Aas26_COI		Lsh10	MZ456435	AAS15_16S	MZ467225	Hap42
Croatia: Motičnjak	Aas01_COI		Lsh6	MZ456436	AAS10_16S	MZ467226	Hap58
Croatia: Motičnjak	Aas01_COI		Lsh6	MZ456437			
Croatia: Motičnjak	Aas01_COI		Lsh6	MZ456438			
Croatia: Motičnjak	Aas26_COI		Lsh10	MZ456439			
Croatia: Motičnjak	Aas01_COI		Lsh6	MZ456440	AAS10_16S	MZ467227	Hap58
Croatia: Motičnjak	Aas26_COI		Lsh10	MZ456441	AAS15_16S	MZ467228	Hap42
Croatia: Mrežnica	Aas19_COI	KF888322	Lsh1	MW726635	Aas10_16S	KF888288	Hap27
Croatia: Mrežnica	Aas19_COI	KF888314	Lsh1	MW726634	Aas10_16S	KF888288	Hap27
Croatia: Mrzlovodičko jezero	Aas20_COI		Lsh2	MZ456410	AAS10_16S	MZ467193	Hap28
Croatia: Mrzlovodičko jezero	Aas20_COI		Lsh2	MZ456411	AAS10_16S	MZ467194	Hap28
Croatia: Mrzlovodičko jezero	Aas20_COI		Lsh2	MZ456412	AAS10_16S	MZ467195	Hap28
Croatia: Mrzlovodičko jezero	Aas20_COI		Lsh2	MZ456413	AAS10_16S	MZ467196	Hap28
Croatia: Mrzlovodičko jezero	Aas20_COI		Lsh2	MZ456414	AAS10_16S	MZ467197	Hap28
Croatia: Mrzlovodičko jezero					AAS10_16S	MZ467198	
Croatia: NP Plitvice Lakes	Aas20_COI		Lsh2	MW726392			
Croatia: NP Plitvice Lakes	Aas20_COI		Lsh2	MW726391			
Croatia: NP Plitvice Lakes	Aas20_COI		Lsh2	MW726390			
Croatia: NP Plitvice Lakes	Aas20_COI		Lsh2	MW726389			
Croatia: NP Plitvice Lakes	Aas20_COI	KF888314	Lsh2	MW726631	Aas10_16S	KF888288	Hap28
Croatia: NP Plitvice Lakes	Aas20_COI	KF888315	Lsh2	MW726630	Aas10_16S	KF888288	Hap28
Croatia: Otočac, hatchery	Aas20_COI		Lsh2	MZ456523			
Croatia: Otočac, hatchery	Aas20_COI		Lsh2	MZ456524			
Croatia: Otočac, hatchery	Aas20_COI		Lsh2	MZ456525			
Croatia: Otuča	Aas21_COI		Lsh4	MZ456405	Aas10_16S	MZ467186	Hap29
Croatia: Otuča	Aas26_COI		Lsh10	MZ456406	AAS15_16S	MZ467187	Hap42
Croatia: Otuča	Aas27_COI		Lsh14	MZ456407	AAS15_16S	MZ467188	Hap43

Croatia: Otuča					AAS15_16S	MZ467189	
Croatia: Otuča	Aas27_COI		Lsh14	MZ456408	AAS15_16S	MZ467190	Hap43
Croatia: Otuča					AAS15_16S	MZ467191	
Croatia: Otuča	Aas27_COI		Lsh14	MZ456409	AAS15_16S	MZ467192	Hap43
Croatia: Otuča	Aas27_COI		Lsh14	MZ456526			
Croatia: Paklenica			Lsh10	MW726488	Aas15_16S	MW726247	Hap42
Croatia: Paklenica	Aas26_COI	KF888321	Lsh10	MW726487	Aas15_16S	KF888293	Hap42
Croatia: Pakra			Lsh10	MW726484			
Croatia: Pakra	Aas26_COI	KF888321			Aas15_16S	KF888293	Hap42
Croatia: Pakra	Aas26_COI		Lsh10	MZ456417	AAS15_16S	MZ467203	Hap42
Croatia: Pazinčica	AAS35_COI		Lsh18	MZ456423	AAS17_16S	MZ467211	Hap51
Croatia: Peratovica	Aas26_COI		Lsh10	MZ456447	AAS15_16S	MZ467233	Hap42
Croatia: Peratovica	Aas26_COI		Lsh10	MZ456448	AAS15_16S	MZ467234	Hap42
Croatia: Peratovica	Aas26_COI		Lsh10	MZ456449	AAS15_16S	MZ467235	Hap42
Croatia: Peratovica	Aas26_COI		Lsh10	MZ456450	AAS15_16S	MZ467236	Hap42
Croatia: Potok Orejovec	Aas26_COI		Lsh10	MZ456427	AAS15_16S	MZ467215	Hap42
Croatia: Potok Orejovec	Aas26_COI		Lsh10	MZ456428	AAS15_16S	MZ467216	Hap42
Croatia: Rakov Creek	Aas30_COI	KF888321	Lsh13	MW726493	Aas17_16S	KF888295	Hap46
Croatia: Ribnjak Creek, Vladisovo	Aas26_COI	KF888321	Lsh10	MW726482	Aas15_16S	KF888293	Hap42
Croatia: Ribnjak Creek, Vladisovo	Aas26_COI	KF888321	Lsh10	MW726481	Aas15_16S	KF888293	Hap42
Croatia: Ribnjak Creek, Vladisovo	Aas27_COI	KF888322	Lsh15	MW726480	Aas15_16S	KF888293	Hap43
Croatia: Ričica River	Aas29_COI	KF888324			Aas16_16S	KF888294	Hap45
Croatia: Sloboština	Aas26_COI		Lsh10	MZ456420	AAS15_16S	MZ467207	Hap42
Croatia: Sloboština	Aas26_COI		Lsh10	MZ456497			
Croatia: Sloboština	Aas26_COI		Lsh10	MZ456498			
Croatia: Sloboština	Aas26_COI		Lsh10	MZ456499	AAS15_16S	MZ467267	Hap42
Croatia: Sloboština	Aas26_COI		Lsh10	MZ456500			
Croatia: Sloboština	Aas26_COI		Lsh10	MZ456501	AAS15_16S	MZ467268	Hap42
Croatia: Sloboština	Aas26_COI		Lsh10	MZ456502	AAS15_16S	MZ467269	Hap42
Croatia: Sloboština	Aas26_COI		Lsh10	MZ456503	AAS15_16S	MZ467270	Hap42
Croatia: Sloboština	AAS39_COI		Lsh22	MZ456504	AAS16_16S	MZ467271	Hap57
Croatia: Subocka River	Aas26_COI	KF888321	Lsh10	MW726486	Aas15_16S	KF888293	Hap42
Croatia: Šumetlica Creek	Aas26_COI	KF888321	Lsh10	MW726479	Aas15_16S	KF888293	Hap42
Croatia: Šumetlica Creek	Aas26_COI	KF888321	Lsh10	MW726478	Aas15_16S	KF888293	Hap42
Croatia: Šumetlica Creek	Aas26_COI	KF888321			Aas15_16S	KF888293	Hap42
Croatia: Trećak Creek	Aas26_COI		Lsh10	MW726483	Aas15_16S	MW726244	Hap42
Croatia: Trećak Creek	Aas26_COI		Lsh10	MW726499	Aas15_16S	MW726258	Hap42
Croatia: Trećak Creek	Aas26_COI	KF888321			Aas15_16S	KF888293	Hap42
Croatia: Trećak Creek	Aas26_COI	KF888321			Aas15_16S	KF888293	Hap42
Croatia: tributary of Čeralinica	Aas27_COI		Lsh15	MZ456426	AAS15_16S	MZ467214	Hap43
Croatia: tributary of Vrela	Aas27_COI		Lsh15	MZ456429	AAS15_16S	MZ467217	Hap43
Croatia: Veličanka	Aas27_COI		Lsh15	MZ456505	AAS15_16S	MZ467272	Hap43
Croatia: Veličanka	Aas27_COI		Lsh15	MZ456506	AAS15_16S	MZ467273	Hap43
Croatia: Veličanka	Aas27_COI		Lsh15	MZ456507	AAS15_16S	MZ467274	Hap43
Croatia: Veličanka	Aas27_COI		Lsh15	MZ456508			
Croatia: Veličanka	Aas27_COI		Lsh15	MZ456509			
Croatia: Veličanka	Aas27_COI		Lsh15	MZ456510			
Croatia: Veličanka	Aas27_COI		Lsh15	MZ456511			
Croatia: Veličanka	Aas27_COI		Lsh15	MZ456512			
Croatia: Veličanka	Aas27_COI		Lsh15	MZ456513			
Croatia: Veličanka	Aas27_COI		Lsh15	MZ456514			
Croatia: Veličanka	Aas27_COI		Lsh15	MZ456515	AAS15_16S	MZ467275	Hap43
Croatia: Vuka River, Razbojište	Aas27_COI		Lsh15	MW726456			
Croatia: Vuka River, Razbojište	Aas27_COI		Lsh15	MW726455			

Croatia: Vuka River, Razbojište	Aas27_COI		Lsh15	MW726454			
Croatia: Vuka River, Razbojište	Aas27_COI		Lsh15	MW726453			
Croatia: Vuka River, Razbojište	Aas27_COI		Lsh15	MW726452			
Croatia: Vuka River, Razbojište	Aas27_COI		Lsh15	MW726451			
Croatia: Vuka River, Razbojište	Aas27_COI		Lsh15	MW726450			
Croatia: Vuka River, Razbojište	Aas27_COI		Lsh15	MW726449			
Croatia: Vuka River, Razbojište	Aas27_COI		Lsh15	MW726448			
Croatia: Vuka River, Razbojište	Aas27_COI		Lsh15	MW726447			
Croatia: Vuka River, Razbojište	Aas27_COI		Lsh15	MW726446			
Croatia: Vuka River, Razbojište	Aas27_COI		Lsh15	MW726445			
Croatia: Vuka River, Razbojište	Aas27_COI		Lsh15	MW726444			
Croatia: Vuka River, Razbojište	Aas27_COI		Lsh15	MW726443			
Croatia: Vuka River, Razbojište	Aas27_COI		Lsh15	MW726442			
Croatia: Vuka River, Razbojište	Aas27_COI		Lsh15	MW726441			
Croatia: Vukovina					AAS15_16S	MZ467280	
Croatia:Lomnica	Aas26_COI		Lsh10	MZ456419	AAS15_16S	MZ467206	Hap42
Czech Republic: Kramata	Aas01_COI	KF888296			Aas01_16S	KF888279	Hap01
Czech Republic: Kramata	Aas01_COI	KF888296			Aas01_16S	KF888279	Hap01
Czech Republic: Kramata	Aas01_COI	KF888296			Aas01_16S	KF888279	Hap01
Czech Republic: Kramata	Aas01_COI	KF888296			Aas01_16S	KF888279	Hap01
Czech Republic: Kramata	Aas01_COI	KF888296			Aas01_16S	KF888279	Hap01
Czech Republic: Kramata	Aas01_COI	KF888296			Aas01_16S	KF888279	Hap01
Czech Republic: Kramata	Aas01_COI	KF888296			Aas01_16S	KF888279	Hap01
Czech Republic: Svetlohor	Aas01_COI	KF888296			Aas01_16S	KF888279	Hap01
Czech Republic: Svetlohor	Aas01_COI	KF888296			Aas01_16S	KF888279	Hap01
Czech Republic: Svetlohor	Aas01_COI	KF888296			Aas01_16S	KF888279	Hap01
Czech Republic: Svetlohor	Aas01_COI	KF888296			Aas01_16S	KF888279	Hap01
Czech Republic: Svetlohor	Aas01_COI	KF888296			Aas01_16S	KF888279	Hap01
Czech Republic: Svetlohor	Aas01_COI	KF888296			Aas01_16S	KF888279	Hap01
Czech Republic: Svetlohor	Aas01_COI	KF888296			Aas01_16S	KF888279	Hap01
Czech Republic: Svetlohor	Aas01_COI	KF888296			Aas01_16S	KF888279	Hap01
Czech Republic: U sudu	Aas01_COI	KF888296			Aas01_16S	KF888279	Hap01
Czech Republic: U sudu	Aas01_COI	KF888296			Aas01_16S	KF888279	Hap01
Czech Republic: U sudu	Aas01_COI	KF888296			Aas01_16S	KF888279	Hap01
Czech Republic: U sudu	Aas01_COI	KF888296			Aas01_16S	KF888279	Hap01
Czech Republic: U sudu	Aas18_COI	KF888313			Aas08_16S	KF888286	Hap25
Czech Republic: U sudu	Aas18_COI	KF888313			Aas08_16S	KF888286	Hap25
Estonia: Lake Pangodi	Aas01_COI		Lsh6	MW726347			
Estonia: Lake Pangodi	Aas01_COI		Lsh6	MW726346			
Estonia: Lake Pangodi	Aas01_COI		Lsh6	MW726345			
Estonia: Lake Pangodi	Aas01_COI		Lsh6	MW726344			
Estonia: Lake Pangodi	Aas01_COI		Lsh6	MW726343			
Estonia: Lake Pangodi	Aas01_COI		Lsh6	MW726342			
Estonia: Lake Pangodi	Aas01_COI		Lsh6	MW726341			
Estonia: Lake Pangodi	Aas01_COI		Lsh6	MW726340			
Estonia: Lake Pangodi	Aas01_COI		Lsh6	MW726339			
Estonia: Lake Pangodi	Aas01_COI		Lsh6	MW726338			
Estonia: Oju River	Aas01_COI		Lsh6	MW726357			
Estonia: Oju River	Aas01_COI		Lsh6	MW726356			
Estonia: Oju River	Aas01_COI		Lsh6	MW726355			
Estonia: Oju River	Aas01_COI		Lsh6	MW726354			
Estonia: Oju River	Aas01_COI		Lsh6	MW726353			
Estonia: Oju River	Aas01_COI		Lsh6	MW726352			
Estonia: Oju River	Aas01_COI		Lsh6	MW726351			

Estonia: Oju River	Aas01_COI		Lsh6	MW726350			
Estonia: Oju River	Aas01_COI		Lsh6	MW726349			
Estonia: Oju River	Aas01_COI		Lsh6	MW726348			
Finland: Valkeinen	Aas01_COI	KF888296			Aas01_16S	KF888279	Hap01
Finland: Valkeinen	Aas01_COI	KF888296			Aas01_16S	KF888279	Hap01
Finland: Valkeinen	Aas01_COI	KF888296			Aas01_16S	KF888279	Hap01
Finland: Valkeinen	Aas01_COI	KF888296			Aas03_16S	KF888281	Hap07
Finland: Valkeinen	Aas01_COI	KF888296			Aas03_16S	KF888281	Hap07
Finland: Yla	Aas01_COI	KF888296			Aas01_16S	KF888279	Hap01
Finland: Yla	Aas01_COI	KF888296			Aas01_16S	KF888279	Hap01
Finland: Yla	Aas01_COI	KF888296			Aas01_16S	KF888279	Hap01
Finland: Yla	Aas01_COI	KF888296			Aas01_16S	KF888279	Hap01
Finland: Yla	Aas01_COI	KF888296			Aas01_16S	KF888279	Hap01
France: Breuvannes-en-Bassigny	Aas01_COI	KF888296			Aas01_16S	KF888279	Hap01
France: Breuvannes-en-Bassigny	Aas01_COI	KF888296			Aas01_16S	KF888279	Hap01
France: Breuvannes-en-Bassigny	Aas01_COI	KF888296			Aas01_16S	KF888279	Hap01
France: Breuvannes-en-Bassigny	Aas01_COI	KF888296			Aas01_16S	KF888279	Hap01
France: Breuvannes-en-Bassigny	Aas01_COI	KF888296			Aas01_16S	KF888279	Hap01
France: Breuvannes-en-Bassigny	Aas01_COI	KF888296			Aas01_16S	KF888279	Hap01
France: Breuvannes-en-Bassigny	Aas01_COI	KF888296			Aas01_16S	KF888279	Hap01
France: Breuvannes-en-Bassigny	Aas01_COI	KF888296			Aas01_16S	KF888279	Hap01
France: Breuvannes-en-Bassigny	Aas01_COI	KF888296			Aas01_16S	KF888279	Hap01
France: Breuvannes-en-Bassigny	Aas01_COI	KF888296			Aas01_16S	KF888279	Hap01
France: Contrexéville	Aas01_COI	KF888296			Aas01_16S	KF888279	Hap01
France: Contrexéville	Aas01_COI	KF888296			Aas01_16S	KF888279	Hap01
France: Contrexéville	Aas01_COI	KF888296			Aas01_16S	KF888279	Hap01
France: Contrexéville	Aas01_COI	KF888296			Aas01_16S	KF888279	Hap01
France: Contrexéville	Aas01_COI	KF888296			Aas01_16S	KF888279	Hap01
France: Contrexéville	Aas01_COI	KF888296			Aas01_16S	KF888279	Hap01
France: Contrexéville	Aas01_COI	KF888296			Aas01_16S	KF888279	Hap01
France: Contrexéville	Aas01_COI	KF888296			Aas01_16S	KF888279	Hap01
France: Contrexéville	Aas01_COI	KF888296			Aas01_16S	KF888279	Hap01
France: Contrexéville	Aas01_COI	KF888296			Aas01_16S	KF888279	Hap01
France: Lemberg	Aas01_COI	KF888296			Aas01_16S	KF888279	Hap01
France: Lemberg	Aas01_COI	KF888296			Aas01_16S	KF888279	Hap01
France: Lemberg	Aas01_COI	KF888296			Aas01_16S	KF888279	Hap01
France: Lemberg	Aas01_COI	KF888296			Aas01_16S	KF888279	Hap01
France: Lemberg	Aas21_COI	KF888316			Aas01_16S	KF888279	Hap31
France: Lemberg	Aas21_COI	KF888316			Aas01_16S	KF888279	Hap31
France: Lemberg	Aas21_COI	KF888316			Aas01_16S	KF888279	Hap31
France: Lemberg	Aas21_COI	KF888316			Aas01_16S	KF888279	Hap31
France: Lemberg	Aas01_COI	KF888321			Aas14_16S	KF888292	Hap41
France: Lemberg	Aas01_COI	KF888321			Aas14_16S	KF888292	Hap42
France: Meisenthal	Aas01_COI	KF888296			Aas01_16S	KF888279	Hap01
France: Meisenthal	Aas01_COI	KF888296			Aas01_16S	KF888279	Hap01
France: Meisenthal	Aas01_COI	KF888296			Aas01_16S	KF888279	Hap01
France: Meisenthal	Aas01_COI	KF888296			Aas01_16S	KF888279	Hap01
France: Meisenthal	Aas01_COI	KF888296			Aas01_16S	KF888279	Hap01
France: Meisenthal	Aas01_COI	KF888296			Aas01_16S	KF888279	Hap01
France: Meisenthal	Aas01_COI	KF888296			Aas01_16S	KF888279	Hap01
France: Meisenthal	Aas01_COI	KF888296			Aas01_16S	KF888279	Hap01
France: Meisenthal	Aas01_COI	KF888321			Aas14_16S	KF888292	Hap41
France: Meisenthal	Aas01_COI	KF888321			Aas14_16S	KF888292	Hap41
France: Rocquigny	Aas01_COI	KF888296			Aas01_16S	KF888279	Hap01

France: Rocquigny	Aas01_COI	KF888296	Aas01_16S	KF888279	Hap01
France: Rocquigny	Aas01_COI	KF888296	Aas01_16S	KF888279	Hap01
France: Rocquigny	Aas01_COI	KF888296	Aas01_16S	KF888279	Hap01
France: Rocquigny	Aas01_COI	KF888296	Aas01_16S	KF888279	Hap01
France: Rocquigny	Aas01_COI	KF888296	Aas01_16S	KF888279	Hap01
France: Rocquigny	Aas01_COI	KF888296	Aas01_16S	KF888279	Hap01
France: Rocquigny	Aas01_COI	KF888296	Aas01_16S	KF888279	Hap01
France: Rocquigny	Aas01_COI	KF888296	Aas01_16S	KF888279	Hap01
France: Rocquigny	Aas01_COI	KF888296	Aas01_16S	KF888279	Hap01
France: Signy-le-Petit	Aas01_COI	KF888296	Aas01_16S	KF888279	Hap01
France: Signy-le-Petit	Aas01_COI	KF888296	Aas01_16S	KF888279	Hap01
France: Signy-le-Petit	Aas01_COI	KF888296	Aas01_16S	KF888279	Hap01
France: Signy-le-Petit	Aas01_COI	KF888296	Aas01_16S	KF888279	Hap01
France: Signy-le-Petit	Aas01_COI	KF888296	Aas01_16S	KF888279	Hap01
France: Signy-le-Petit	Aas01_COI	KF888296	Aas01_16S	KF888279	Hap01
France: Signy-le-Petit	Aas01_COI	KF888296	Aas01_16S	KF888279	Hap01
France: Signy-le-Petit	Aas01_COI	KF888296	Aas01_16S	KF888279	Hap01
France: Signy-le-Petit	Aas01_COI	KF888296	Aas01_16S	KF888279	Hap01
France: Signy-le-Petit	Aas01_COI	KF888296	Aas01_16S	KF888279	Hap01
France: Sturzelbronn	Aas01_COI	KF888296	Aas01_16S	KF888279	Hap01
France: Sturzelbronn	Aas01_COI	KF888296	Aas01_16S	KF888279	Hap01
France: Sturzelbronn	Aas01_COI	KF888296	Aas01_16S	KF888279	Hap01
France: Sturzelbronn	Aas01_COI	KF888296	Aas01_16S	KF888279	Hap01
France: Sturzelbronn	Aas01_COI	KF888296	Aas01_16S	KF888279	Hap01
France: Sturzelbronn	Aas01_COI	KF888296	Aas01_16S	KF888279	Hap01
France: Sturzelbronn	Aas21_COI	KF888316	Aas01_16S	KF888279	Hap31
France: Sturzelbronn	Aas21_COI	KF888316	Aas01_16S	KF888279	Hap31
France: Sturzelbronn	Aas21_COI	KF888316	Aas01_16S	KF888279	Hap31
France: Sturzelbronn	Aas21_COI	KF888316	Aas01_16S	KF888279	Hap31
Germany: Aachen	Aas01_COI	KF888296	Aas01_16S	KF888279	Hap01
Germany: Aachen	Aas01_COI	KF888296	Aas01_16S	KF888279	Hap01
Germany: Aachen	Aas01_COI	KF888296	Aas01_16S	KF888279	Hap01
Germany: Aachen	Aas01_COI	KF888296	Aas01_16S	KF888279	Hap01
Germany: Aachen	Aas01_COI	KF888296	Aas01_16S	KF888279	Hap01
Germany: Aachen	Aas01_COI	KF888296	Aas01_16S	KF888279	Hap01
Germany: Aachen	Aas01_COI	KF888296	Aas01_16S	KF888279	Hap01
Germany: Aachen	Aas01_COI	KF888296	Aas01_16S	KF888279	Hap01
Germany: Aachen	Aas01_COI	KF888296	Aas01_16S	KF888279	Hap01
Germany: Aachen	Aas01_COI	KF888296	Aas01_16S	KF888279	Hap01
Germany: Aachen	Aas01_COI	KF888296	Aas01_16S	KF888279	Hap01
Germany: Aachen	Aas01_COI	KF888296	Aas01_16S	KF888279	Hap01
Germany: Aachen	Aas01_COI	KF888296	Aas01_16S	KF888279	Hap01
Germany: Aachen	Aas01_COI	KF888296	Aas01_16S	KF888279	Hap01
Germany: Aachen	Aas01_COI	KF888296	Aas01_16S	KF888279	Hap01
Germany: Aachen	Aas01_COI	KF888296	Aas01_16S	KF888279	Hap01
Germany: Aachen	Aas01_COI	KF888296	Aas01_16S	KF888279	Hap01
Germany: Aachen	Aas01_COI	KF888296	Aas01_16S	KF888279	Hap01
Germany: Aachen	Aas01_COI	KF888296	Aas01_16S	KF888279	Hap01
Germany: Aachen	Aas01_COI	KF888296	Aas01_16S	KF888279	Hap01
Germany: Aar	Aas01_COI	KF888296	Aas01_16S	KF888279	Hap01
Germany: Aar	Aas01_COI	KF888296	Aas01_16S	KF888279	Hap01
Germany: Aar	Aas01_COI	KF888296	Aas01_16S	KF888279	Hap01
Germany: Aar	Aas01_COI	KF888296	Aas01_16S	KF888279	Hap01
Germany: Aar	Aas01_COI	KF888296	Aas01_16S	KF888279	Hap01

Germany: Allna	Aas01_COI	KF888296	Aas01_16S	KF888279	Hap01
Germany: Allna	Aas01_COI	KF888296	Aas01_16S	KF888279	Hap01
Germany: Allna	Aas01_COI	KF888296	Aas01_16S	KF888279	Hap01
Germany: Allna	Aas01_COI	KF888296	Aas07_16S	KF888285	Hap21
Germany: Ambach	Aas01_COI	KF888296	Aas01_16S	KF888279	Hap01
Germany: Ambach	Aas01_COI	KF888296	Aas01_16S	KF888279	Hap01
Germany: Ambach	Aas01_COI	KF888296	Aas01_16S	KF888279	Hap01
Germany: Ambach	Aas01_COI	KF888296	Aas01_16S	KF888279	Hap01
Germany: Ambach	Aas01_COI	KF888296	Aas01_16S	KF888279	Hap01
Germany: Barthe	Aas12_COI	KF888307	Aas01_16S	KF888279	Hap16
Germany: Barthe	Aas12_COI	KF888307	Aas01_16S	KF888279	Hap16
Germany: Barthe	Aas12_COI	KF888307	Aas01_16S	KF888279	Hap16
Germany: Barthe	Aas12_COI	KF888307	Aas01_16S	KF888279	Hap16
Germany: Barthe	Aas12_COI	KF888307	Aas01_16S	KF888279	Hap16
Germany: Breitweiher/Rhön	Aas01_COI	KF888296	Aas01_16S	KF888279	Hap01
Germany: Breitweiher/Rhön	Aas01_COI	KF888296	Aas01_16S	KF888279	Hap01
Germany: Clausthal-Zellerfeld*	Aas01_COI	JN254659			
Germany: Dautphe	Aas01_COI	KF888296	Aas01_16S	KF888279	Hap01
Germany: Dautphe	Aas01_COI	KF888296	Aas01_16S	KF888279	Hap01
Germany: Dautphe	Aas01_COI	KF888296	Aas01_16S	KF888279	Hap01
Germany: Dautphe	Aas01_COI	KF888296	Aas01_16S	KF888279	Hap01
Germany: Dielbach (Woog)	Aas01_COI	KF888296	Aas01_16S	KF888279	Hap01
Germany: Dielbach (Woog)	Aas01_COI	KF888296	Aas01_16S	KF888279	Hap01
Germany: Dielbach (Woog)	Aas01_COI	KF888296	Aas01_16S	KF888279	Hap01
Germany: Dielbach (Woog)	Aas04_COI	KF888299	Aas01_16S	KF888279	Hap05
Germany: Dielbach (Woog)	Aas21_COI	KF888316	Aas01_16S	KF888279	Hap31
Germany: Dielbach (Woog)	Aas21_COI	KF888316	Aas01_16S	KF888279	Hap31
Germany: Dielbach (Woog)	Aas21_COI	KF888316	Aas01_16S	KF888279	Hap31
Germany: Dielbach (Woog)	Aas26_COI	KF888321	Aas14_16S	KF888292	Hap41
Germany: Donsbach	Aas01_COI	KF888296	Aas01_16S	KF888279	Hap01
Germany: Donsbach	Aas01_COI	KF888296	Aas01_16S	KF888279	Hap01
Germany: Donsbach	Aas01_COI	KF888296	Aas01_16S	KF888279	Hap01
Germany: Donsbach	Aas01_COI	KF888296	Aas01_16S	KF888279	Hap01
Germany: Donsbach	Aas01_COI	KF888296	Aas01_16S	KF888279	Hap01
Germany: Eichelbach	Aas01_COI	KF888296	Aas01_16S	KF888279	Hap01
Germany: Eichelbach	Aas01_COI	KF888296	Aas01_16S	KF888279	Hap01
Germany: Eichelbach	Aas01_COI	KF888296	Aas01_16S	KF888279	Hap01
Germany: Eichelbach	Aas01_COI	KF888296	Aas01_16S	KF888279	Hap01
Germany: Eichelbach	Aas01_COI	KF888296	Aas01_16S	KF888279	Hap01
Germany: Eifel	Aas01_COI	KF888296	Aas01_16S	KF888279	Hap01
Germany: Eifel	Aas20_COI	KF888315	Aas03_16S	KF888281	Hap32
Germany: Farm Augsburg	Aas01_COI	KF888296	Aas01_16S	KF888279	Hap01
Germany: Farm Augsburg	Aas01_COI	KF888296	Aas01_16S	KF888279	Hap01
Germany: Farm Augsburg	Aas01_COI	KF888296	Aas01_16S	KF888279	Hap01
Germany: Farm Augsburg	Aas01_COI	KF888296	Aas01_16S	KF888279	Hap01
Germany: Farm Augsburg	Aas01_COI	KF888296	Aas01_16S	KF888279	Hap01
Germany: Farm Augsburg	Aas01_COI	KF888296	Aas01_16S	KF888279	Hap01
Germany: Farm Augsburg	Aas01_COI	KF888296	Aas01_16S	KF888279	Hap01
Germany: Farm Augsburg	Aas01_COI	KF888296	Aas01_16S	KF888279	Hap01
Germany: Farm Augsburg	Aas05_COI	KF888300	Aas01_16S	KF888279	Hap06
Germany: Farm Augsburg	Aas05_COI	KF888300	Aas01_16S	KF888279	Hap06
Germany: Farm Augsburg	Aas05_COI	KF888300	Aas01_16S	KF888279	Hap06
Germany: Farm Augsburg	Aas01_COI	KF888296	Aas03_16S	KF888281	Hap07
Germany: Farm Augsburg	Aas06_COI	KF888301	Aas01_16S	KF888279	Hap08

Germany: Farm Augsburg	Aas20_COI	KF888315	Aas03_16S	KF888281	Hap32
Germany: Farm Augsburg	Aas20_COI	KF888315	Aas03_16S	KF888281	Hap32
Germany: Farm Oeversee	Aas02_COI	KF888297	Aas01_16S	KF888279	Hap03
Germany: Farm Oeversee	Aas02_COI	KF888297	Aas01_16S	KF888279	Hap03
Germany: Farm Oeversee	Aas02_COI	KF888297	Aas01_16S	KF888279	Hap03
Germany: Farm Oeversee	Aas02_COI	KF888297	Aas01_16S	KF888279	Hap03
Germany: Farm Oeversee	Aas03_COI	KF888298	Aas01_16S	KF888279	Hap04
Germany: Fischbach	Aas01_COI	KF888296	Aas01_16S	KF888279	Hap01
Germany: Fischbach	Aas01_COI	KF888296	Aas01_16S	KF888279	Hap01
Germany: Fischbach	Aas01_COI	KF888296	Aas01_16S	KF888279	Hap01
Germany: Fischbach	Aas22_COI	KF888317	Aas01_16S	KF888279	Hap30
Germany: Fischbach	Aas22_COI	KF888317	Aas01_16S	KF888279	Hap30
Germany: Fischbach	Aas22_COI	KF888317	Aas01_16S	KF888279	Hap30
Germany: Fohnbach	Aas01_COI	KF888296	Aas01_16S	KF888279	Hap01
Germany: Fohnbach	Aas01_COI	KF888296	Aas01_16S	KF888279	Hap01
Germany: Fohnbach	Aas01_COI	KF888296	Aas01_16S	KF888279	Hap01
Germany: Fohnbach	Aas01_COI	KF888296	Aas01_16S	KF888279	Hap01
Germany: Fohnbach	Aas01_COI	KF888296	Aas01_16S	KF888279	Hap01
Germany: Gansbach	Aas01_COI	KF888296	Aas01_16S	KF888279	Hap01
Germany: Gansbach	Aas01_COI	KF888296	Aas01_16S	KF888279	Hap01
Germany: Gansbach	Aas01_COI	KF888296	Aas01_16S	KF888279	Hap01
Germany: Gansbach	Aas01_COI	KF888296	Aas01_16S	KF888279	Hap01
Germany: Gansbach	Aas01_COI	KF888296	Aas01_16S	KF888279	Hap01
Germany: Geierstein/Roth	Aas01_COI	KF888296	Aas01_16S	KF888279	Hap01
Germany: Geierstein/Roth	Aas01_COI	KF888296	Aas01_16S	KF888279	Hap01
Germany: Geierstein/Roth	Aas01_COI	KF888296	Aas01_16S	KF888279	Hap01
Germany: Geierstein/Roth	Aas01_COI	KF888296	Aas01_16S	KF888279	Hap01
Germany: Geierstein/Roth	Aas01_COI	KF888296	Aas01_16S	KF888279	Hap01
Germany: Geierstein/Roth	Aas01_COI	KF888296	Aas01_16S	KF888279	Hap01
Germany: Geierstein/Roth	Aas01_COI	KF888296	Aas01_16S	KF888279	Hap01
Germany: Geierstein/Roth	Aas01_COI	KF888296	Aas01_16S	KF888279	Hap01
Germany: Geierstein/Roth	Aas01_COI	KF888296	Aas01_16S	KF888279	Hap01
Germany: Geierstein/Roth	Aas01_COI	KF888296	Aas01_16S	KF888279	Hap01
Germany: Giebelsbach	Aas01_COI	KF888296	Aas01_16S	KF888279	Hap01
Germany: Gut Rietberg	Aas01_COI	KF888296	Aas01_16S	KF888279	Hap01
Germany: Gut Rietberg	Aas01_COI	KF888296	Aas01_16S	KF888279	Hap01
Germany: Gut Rietberg	Aas01_COI	KF888296	Aas01_16S	KF888279	Hap01
Germany: Gut Rietberg	Aas01_COI	KF888296	Aas01_16S	KF888279	Hap01
Germany: Gut Rietberg	Aas20_COI	KF888315	Aas03_16S	KF888281	Hap32
Germany: Hartmann/Rehbach	Aas01_COI	KF888296	Aas01_16S	KF888279	Hap01
Germany: Hartmann/Rehbach	Aas01_COI	KF888296	Aas01_16S	KF888279	Hap01
Germany: Hartmann/Rehbach	Aas01_COI	KF888296	Aas01_16S	KF888279	Hap01
Germany: Hartmann/Rehbach	Aas01_COI	KF888296	Aas01_16S	KF888279	Hap01
Germany: Hartmann/Rehbach	Aas01_COI	KF888296	Aas01_16S	KF888279	Hap01
Germany: Jäglitz	Aas01_COI	KF888296	Aas01_16S	KF888279	Hap01
Germany: Jäglitz	Aas01_COI	KF888296	Aas01_16S	KF888279	Hap01
Germany: Jäglitz	Aas01_COI	KF888296	Aas01_16S	KF888279	Hap01
Germany: Jäglitz	Aas01_COI	KF888296	Aas01_16S	KF888279	Hap01
Germany: Jäglitz	Aas01_COI	KF888296	Aas01_16S	KF888279	Hap01
Germany: Jäglitz	Aas26_COI	KF888321	Aas14_16S	KF888292	Hap41
Germany: Jäglitz	Aas26_COI	KF888321	Aas14_16S	KF888292	Hap41
Germany: Kallenbach	Aas01_COI	KF888296	Aas01_16S	KF888279	Hap01
Germany: Kallenbach	Aas01_COI	KF888296	Aas01_16S	KF888279	Hap01
Germany: Kallenbach	Aas01_COI	KF888296	Aas01_16S	KF888279	Hap01

Germany: Kallenbach	Aas01_COI	KF888296	Aas01_16S	KF888279	Hap01
Germany: Kallenbach	Aas01_COI	KF888296	Aas01_16S	KF888279	Hap01
Germany: Klausbach	Aas01_COI	KF888296	Aas01_16S	KF888279	Hap01
Germany: Klausbach	Aas01_COI	KF888296	Aas01_16S	KF888279	Hap01
Germany: Klausbach	Aas01_COI	KF888296	Aas01_16S	KF888279	Hap01
Germany: Klausbach	Aas20_COI	KF888315	Aas03_16S	KF888281	Hap32
Germany: Klausbach	Aas20_COI	KF888315	Aas03_16S	KF888281	Hap32
Germany: Klausbach	Aas20_COI	KF888315	Aas03_16S	KF888281	Hap32
Germany: Klausbach	Aas20_COI	KF888315	Aas03_16S	KF888281	Hap32
Germany: Koppelsee	Aas01_COI	KF888296	Aas01_16S	KF888279	Hap01
Germany: Koppelsee	Aas01_COI	KF888296	Aas01_16S	KF888279	Hap01
Germany: Koppelsee	Aas01_COI	KF888296	Aas01_16S	KF888279	Hap01
Germany: Langsee	Aas02_COI	KF888297	Aas01_16S	KF888279	Hap03
Germany: Langsee	Aas02_COI	KF888297	Aas01_16S	KF888279	Hap03
Germany: Langsee	Aas02_COI	KF888297	Aas01_16S	KF888279	Hap03
Germany: Langsee	Aas02_COI	KF888297	Aas01_16S	KF888279	Hap03
Germany: Langsee	Aas02_COI	KF888297	Aas01_16S	KF888279	Hap03
Germany: Langsee	Aas02_COI	KF888297	Aas01_16S	KF888279	Hap03
Germany: Langsee	Aas02_COI	KF888297	Aas01_16S	KF888279	Hap03
Germany: Langsee	Aas02_COI	KF888297	Aas01_16S	KF888279	Hap03
Germany: Langsee	Aas03_COI	KF888298	Aas01_16S	KF888279	Hap04
Germany: Lasterbach	Aas01_COI	KF888296	Aas01_16S	KF888279	Hap01
Germany: Lasterbach	Aas01_COI	KF888296	Aas01_16S	KF888279	Hap01
Germany: Lasterbach	Aas01_COI	KF888296	Aas01_16S	KF888279	Hap01
Germany: Lasterbach	Aas01_COI	KF888296	Aas01_16S	KF888279	Hap01
Germany: Lasterbach	Aas01_COI	KF888296	Aas01_16S	KF888279	Hap01
Germany: Madenmühlen	Aas01_COI	KF888296	Aas01_16S	KF888279	Hap01
Germany: Madenmühlen	Aas01_COI	KF888296	Aas01_16S	KF888279	Hap01
Germany: Madenmühlen	Aas01_COI	KF888296	Aas01_16S	KF888279	Hap01
Germany: Madenmühlen	Aas01_COI	KF888296	Aas01_16S	KF888279	Hap01
Germany: Madenmühlen	Aas01_COI	KF888296	Aas01_16S	KF888279	Hap01
Germany: Madenmühlen 2	Aas01_COI	KF888296	Aas01_16S	KF888279	Hap01
Germany: Madenmühlen 2	Aas01_COI	KF888296	Aas01_16S	KF888279	Hap01
Germany: Madenmühlen 2	Aas01_COI	KF888296	Aas01_16S	KF888279	Hap01
Germany: Madenmühlen 2	Aas01_COI	KF888296	Aas01_16S	KF888279	Hap01
Germany: Mahlscheid	Aas01_COI	KF888296	Aas01_16S	KF888279	Hap01
Germany: Mahlscheid	Aas01_COI	KF888296	Aas01_16S	KF888279	Hap01
Germany: Mahlscheid	Aas01_COI	KF888296	Aas01_16S	KF888279	Hap01
Germany: Mahlscheid	Aas01_COI	KF888296	Aas01_16S	KF888279	Hap01
Germany: Mahlscheid	Aas01_COI	KF888296	Aas01_16S	KF888279	Hap01
Germany: Mahlscheid	Aas01_COI	KF888296	Aas01_16S	KF888279	Hap01
Germany: Meerbach	Aas01_COI	KF888296	Aas01_16S	KF888279	Hap01
Germany: Meisertalweiher	Aas01_COI	KF888296	Aas01_16S	KF888279	Hap01
Germany: Meisertalweiher	Aas01_COI	KF888296	Aas01_16S	KF888279	Hap01
Germany: Meisertalweiher	Aas01_COI	KF888296	Aas01_16S	KF888279	Hap01
Germany: Meisertalweiher	Aas01_COI	KF888296	Aas01_16S	KF888279	Hap01
Germany: Meisertalweiher	Aas01_COI	KF888296	Aas01_16S	KF888279	Hap01
Germany: Merzkrebse	Aas01_COI	KF888296	Aas01_16S	KF888279	Hap01
Germany: Merzkrebse	Aas01_COI	KF888296	Aas01_16S	KF888279	Hap01
Germany: Merzkrebse	Aas01_COI	KF888296	Aas01_16S	KF888279	Hap01
Germany: Merzkrebse	Aas01_COI	KF888296	Aas01_16S	KF888279	Hap01
Germany: Merzkrebse	Aas01_COI	KF888296	Aas01_16S	KF888279	Hap01
Germany: Mühlgraben Caldern	Aas01_COI	KF888296	Aas01_16S	KF888279	Hap01
Germany: Mühlgraben Caldern	Aas01_COI	KF888296	Aas01_16S	KF888279	Hap01

Germany: Mühlgraben Caldern	Aas05_COI	KF888300	Aas01_16S	KF888279	Hap06
Germany: Nanzenbach	Aas01_COI	KF888296	Aas01_16S	KF888279	Hap01
Germany: Ocherbach	Aas01_COI	KF888296	Aas01_16S	KF888279	Hap01
Germany: Ocherbach	Aas01_COI	KF888296	Aas01_16S	KF888279	Hap01
Germany: Ocherbach	Aas01_COI	KF888296	Aas01_16S	KF888279	Hap01
Germany: Ocherbach	Aas01_COI	KF888296	Aas01_16S	KF888279	Hap01
Germany: Ocherbach	Aas01_COI	KF888296	Aas01_16S	KF888279	Hap01
Germany: Perf	Aas01_COI	KF888296	Aas01_16S	KF888279	Hap01
Germany: Perf	Aas01_COI	KF888296	Aas01_16S	KF888279	Hap01
Germany: Perf	Aas01_COI	KF888296	Aas01_16S	KF888279	Hap01
Germany: Perf	Aas01_COI	KF888296	Aas01_16S	KF888279	Hap01
Germany: Perf	Aas01_COI	KF888296	Aas01_16S	KF888279	Hap01
Germany: Pollichia Woog	Aas20_COI	KF888315	Aas03_16S	KF888281	Hap32
Germany: Pollichia Woog	Aas20_COI	KF888315	Aas03_16S	KF888281	Hap32
Germany: Pollichia Woog2	Aas01_COI	KF888296	Aas01_16S	KF888279	Hap01
Germany: Pollichia Woog2	Aas20_COI	KF888315	Aas03_16S	KF888281	Hap32
Germany: Pollichia Woog2	Aas20_COI	KF888315	Aas03_16S	KF888281	Hap32
Germany: Saarbach (Lagerweiher)	Aas01_COI	KF888296	Aas01_16S	KF888279	Hap01
Germany: Saarbach (Woog)	Aas01_COI	KF888296	Aas01_16S	KF888279	Hap01
Germany: Saarbach (Woog)	Aas01_COI	KF888296	Aas01_16S	KF888279	Hap01
Germany: Saarbach (Woog)	Aas01_COI	KF888296	Aas01_16S	KF888279	Hap01
Germany: Saarbach (Woog)	Aas01_COI	KF888296	Aas01_16S	KF888279	Hap01
Germany: Saarbach (Woog)	Aas21_COI	KF888316	Aas01_16S	KF888279	Hap31
Germany: Saarbach/Saar-bachhammer	Aas21_COI	KF888316	Aas01_16S	KF888279	Hap31
Germany: Saarbach/Saar-bachhammer	Aas21_COI	KF888316	Aas01_16S	KF888279	Hap31
Germany: Saarbacher Hammer	Aas21_COI	KF888316	Aas01_16S	KF888279	Hap31
Germany: Saarbacher Mühlweiher	Aas21_COI	KF888316	Aas01_16S	KF888279	Hap31
Germany: Saarbacher Mühlweiher	Aas21_COI	KF888316	Aas01_16S	KF888279	Hap31
Germany: Salzbach	Aas01_COI	KF888296	Aas01_16S	KF888279	Hap01
Germany: Salzbach	Aas01_COI	KF888296	Aas01_16S	KF888279	Hap01
Germany: Salzbach	Aas01_COI	KF888296	Aas01_16S	KF888279	Hap01
Germany: Salzbach	Aas01_COI	KF888296	Aas01_16S	KF888279	Hap01
Germany: Salzbach	Aas01_COI	KF888296	Aas01_16S	KF888279	Hap01
Germany: Schlettenbachtal	Aas20_COI	KF888315	Aas03_16S	KF888281	Hap32
Germany: Schwarze Elster	Aas01_COI	KF888296	Aas01_16S	KF888279	Hap01
Germany: Schwarze Elster	Aas01_COI	KF888296	Aas01_16S	KF888279	Hap01
Germany: Schwarze Elster	Aas01_COI	KF888296	Aas01_16S	KF888279	Hap01
Germany: Schwarze Elster	Aas01_COI	KF888296	Aas01_16S	KF888279	Hap01
Germany: Schwarze Elster	Aas01_COI	KF888296	Aas01_16S	KF888279	Hap01
Germany: Schwarze Elster	Aas01_COI	KF888296	Aas01_16S	KF888279	Hap01
Germany: Spielberg	Aas01_COI	KF888296	Aas01_16S	KF888279	Hap01
Germany: Spielberg	Aas01_COI	KF888296	Aas01_16S	KF888279	Hap01
Germany: Spielberg	Aas20_COI	KF888315	Aas03_16S	KF888281	Hap32
Germany: Spielberg	Aas20_COI	KF888315	Aas03_16S	KF888281	Hap32
Germany: Spielberg	Aas20_COI	KF888315	Aas03_16S	KF888281	Hap32
Germany: Steinbruch Rot	Aas01_COI	KF888296	Aas01_16S	KF888279	Hap01
Germany: Steinbruch Rot	Aas01_COI	KF888296	Aas01_16S	KF888279	Hap01
Germany: Steinbruch Rot	Aas01_COI	KF888296	Aas01_16S	KF888279	Hap01
Germany: Steinbruch Rot	Aas01_COI	KF888296	Aas01_16S	KF888279	Hap01
Germany: Steinbruch Rot	Aas01_COI	KF888296	Aas01_16S	KF888279	Hap01
Germany: Stepenitz	Aas01_COI	KF888296	Aas01_16S	KF888279	Hap01
Germany: Stepenitz	Aas01_COI	KF888296	Aas01_16S	KF888279	Hap01
Germany: Stepenitz	Aas01_COI	KF888296	Aas01_16S	KF888279	Hap01
Germany: Stepenitz	Aas01_COI	KF888296	Aas01_16S	KF888279	Hap01

Germany: Stepenitz	Aas01_COI	KF888296	Aas01_16S	KF888279	Hap01
Germany: Stippbach	Aas01_COI	KF888296	Aas01_16S	KF888279	Hap01
Germany: Stippbach	Aas01_COI	KF888296	Aas01_16S	KF888279	Hap01
Germany: Stippbach	Aas01_COI	KF888296	Aas01_16S	KF888279	Hap01
Germany: Stippbach	Aas01_COI	KF888296	Aas01_16S	KF888279	Hap01
Germany: Stippbach	Aas01_COI	KF888296	Aas01_16S	KF888279	Hap01
Germany: Tonkuhle	Aas01_COI	KF888296	Aas01_16S	KF888279	Hap01
Germany: Tonkuhle	Aas01_COI	KF888296	Aas01_16S	KF888279	Hap01
Germany: Tonkuhle	Aas01_COI	KF888296	Aas01_16S	KF888279	Hap01
Germany: Urff	Aas01_COI	KF888296	Aas01_16S	KF888279	Hap01
Germany: Urff	Aas01_COI	KF888296	Aas01_16S	KF888279	Hap01
Germany: Urff	Aas01_COI	KF888296	Aas01_16S	KF888279	Hap01
Germany: Urff	Aas01_COI	KF888296	Aas01_16S	KF888279	Hap01
Germany: Waldteich bei Wallenfels	Aas01_COI	KF888296	Aas01_16S	KF888279	Hap01
Germany: Waldteich bei Wallenfels	Aas01_COI	KF888296	Aas01_16S	KF888279	Hap01
Germany: Waldteich bei Wallenfels	Aas01_COI	KF888296	Aas01_16S	KF888279	Hap01
Germany: Waldteich bei Wallenfels	Aas01_COI	KF888296	Aas01_16S	KF888279	Hap01
Germany: Waldteich bei Wallenfels	Aas01_COI	KF888296	Aas02_16S	KF888280	Hap02
Germany: Waldteich, Irrschelde	Aas01_COI	KF888296	Aas01_16S	KF888279	Hap01
Germany: Waldteich, Irrschelde	Aas01_COI	KF888296	Aas01_16S	KF888279	Hap01
Germany: Waldteich, Irrschelde	Aas01_COI	KF888296	Aas01_16S	KF888279	Hap01
Germany: Waldteich, Irrschelde	Aas01_COI	KF888296	Aas01_16S	KF888279	Hap01
Germany: Waldteich, Irrschelde	Aas01_COI	KF888296	Aas01_16S	KF888279	Hap01
Germany: Wielenbach	Aas01_COI	KF888296	Aas01_16S	KF888279	Hap01
Germany: Wielenbach	Aas01_COI	KF888296	Aas01_16S	KF888279	Hap01
Germany: Wolfsägertal	Aas01_COI	KF888296	Aas01_16S	KF888279	Hap01
Germany: Wolfsägertal	Aas01_COI	KF888296	Aas01_16S	KF888279	Hap01
Germany: Wolfsägertal	Aas04_COI	KF888299	Aas01_16S	KF888279	Hap05
Germany: Wolfsägertal	Aas21_COI	KF888316	Aas01_16S	KF888279	Hap31
Germany: Wolfsägertal	Aas21_COI	KF888316	Aas01_16S	KF888279	Hap31
Germany: Wolfsägertal	Aas20_COI	KF888315	Aas03_16S	KF888281	Hap32
Germany: Wolfsägertal	Aas20_COI	KF888315	Aas03_16S	KF888281	Hap32
Germany: Zoo Zajak, Petstore	Aas01_COI	KF888296	Aas01_16S	KF888279	Hap01
Germany: Zoo Zajak, Petstore	Aas01_COI	KF888296	Aas01_16S	KF888279	Hap01
Germany: Zoo Zajak, Petstore	Aas01_COI	KF888296	Aas01_16S	KF888279	Hap01
Germany: Zoo Zajak, Petstore	Aas01_COI	KF888296	Aas01_16S	KF888279	Hap01
Germany: Zoo Zajak, Petstore	Aas01_COI	KF888296	Aas01_16S	KF888279	Hap01
Greece: Aoo1	C5	KY067209	A2	KY048194	A2C5
Greece: Aoo1	C8	KY067210	A2	KY048194	A2C8
Greece: Aoo2	C21	KY067215	A2	KY048194	A2C21
Greece: Aoo2	C50	KY067226	A2	KY048194	A2C50
Greece: Arahthos	C21	KY067215	A2	KY048199	A2C21
Greece: Begoritida/Agra	C15	KY067213	A2	KY048194	A2C15
Greece: Chani Kaber Aga	C21	KY067215	A2	KY048197	A2C21
Greece: Chani Kaber Aga	C21	KY067215	A2	KY048194	A2C21
Greece: Doxa	C2	KY067208	A2	KY048195	A2C2
Greece: Doxa	C2	KY067208	A2	KY048194	A2C2
Greece: Fragkades	C14	KY067212	A2	KY048194	A2C14
Greece: Fragkades	C14	KY067212	A2	KY048194	A2C14
Greece: Kalamas	C29	KY067218	A2	KY048194	A2C29
Greece: Kalamas	C33	KY067222	A2	KY048194	A2C33
Greece: Kalivia	C30	KY067219	A1	KY048198	A1C30
Greece: Kalivia	C35	KY067224	A2	KY048194	A2C35
Greece: Karya	C1	KY067207	A2	KY048202	A2C1
Greece: Karya	C40	KY067225	A2	KY048194	A2C40

Greece: Kefalovriso	C32	KY067221			A3	KY048201	A3C32
Greece: Koniskos	C31	KY067220			A2	KY048194	A2C31
Greece: Koniskos	C31	KY067220			A2	KY048194	A2C31
Greece: Krania	C31	KY067220			A6	KY048194	A6C31
Greece: Krania	C31	KY067220			A2	KY048200	A2C31
Greece: Loggas	C31	KY067220			A2	KY048194	A2C31
Greece: Loggas	C31	KY067220			A2	KY048195	A2C31
Greece: Neochori	C27	KY067216			A2	KY048194	A2C27
Greece: Neochori	C27	KY067216			A2	KY048194	A2C27
Greece: Palaifyto	C28	KY067217			A2	KY048197	A2C28
Greece: Palaifyto	C34	KY067223			A2	KY048194	A2C34
Greece: Perivoli	C10	KY067211			A16	KY048193	A16C10
Greece: Pertouli	C1	KY067207			A2	KY048200	0A2C21
Greece: Skotina	C1	KY067207			A2	KY048194	A2C1
Greece: Skotina	C1	KY067207			A2	KY048194	A2C1
Greece: Tsivlo	C1	KY067207			A4	KY048196	A4C1
Greece: Tsivlo	C51	KY067227			A4	KY048196	A4C51
Greece: Tzaravina	C52	KY067228			A2	KY048194	A2C52
Greece: Tzaravina	C18	KY067214			A2	KY048194	A2C18
Hungary: Kádárta	Aas01_COI	KF888296			Aas01_16S	KF888279	Hap01
Hungary: Kádárta	Aas01_COI	KF888296			Aas01_16S	KF888279	Hap01
Hungary: Kádárta	Aas01_COI	KF888296			Aas01_16S	KF888279	Hap01
Hungary: Pét	Aas07_COI	KF888302			Aas01_16S	KF888279	Hap09
Kosovo: Unnamed stream Ferizaj	Aas16_COI		Lsh3	MW726629	Aas09_16S	MW726330	Hap26
Kosovo: Unnamed stream Ferizaj	Aas16_COI	KF888311			Aas09_16S	KF888287	Hap26
Macedonia: Mantova	AAS34_COI		Lsh17	MZ456377	AAS19_16S	MZ467157	Hap50
Macedonia: Mantova					AAS19_16S	MZ467158	
Macedonia: Mantova	AAS34_COI		Lsh17	MZ456378	AAS19_16S	MZ467159	Hap50
Macedonia: Mantova	AAS34_COI		Lsh17	MZ456379	AAS19_16S	MZ467160	Hap50
Macedonia: Mantova	AAS34_COI		Lsh17	MZ456380	AAS19_16S	MZ467161	Hap50
Macedonia: Mantova	AAS34_COI		Lsh17	MZ456381	AAS19_16S	MZ467162	Hap50
Macedonia: Mantova	AAS34_COI		Lsh17	MZ456382	AAS19_16S	MZ467163	Hap50
Macedonia: Mantova	AAS34_COI		Lsh17	MZ456383	AAS19_16S	MZ467164	Hap50
Macedonia: Mantova	AAS34_COI		Lsh17	MZ456384	AAS19_16S	MZ467165	Hap50
Montenegro: Lake Liverovići, Gračenica	Aas37_COI		Lsh4	MW726626	Aas15_16S	MW726327	Hap42
Montenegro: Lake Liverovići, Gračenica	Aas37_COI		Lsh16	MW726473	Aas15_16S	MW726241	Hap54
Montenegro: Lake Liverovići, Gračenica	Aas26_COI	KF888321			Aas15_16S	KF888293	Hap42
Montenegro: Zeta River	Aas20_COI	KF888325	Lsh2	MW726628	Aas10_16S	KF888288	Hap28
Montenegro: Zeta River	Aas21_COI	KF888316	Lsh4	MW726627	Aas10_16S	KF888288	Hap29
Montenegro: Zeta River	Aas26_COI	KF888321	Lsh10	MW726475	Aas15_16S	KF888293	Hap42
Montenegro: Zeta River	Aas26_COI	KF888321	Lsh10	MW726474	Aas15_16S	KF888293	Hap42
Montenegro: Zeta River	Aas26_COI	KF888321	Lsh10	MW726472	Aas15_16S	KF888293	Hap42
Montenegro: Zeta River	Aas26_COI	KF888321	Lsh10	MW726471	Aas15_16S	KF888293	Hap42
Netherlands: Rhine	Aas01_COI	KF888296			Aas01_16S	KF888279	Hap01
Netherlands: Rhine	Aas01_COI	KF888296			Aas01_16S	KF888279	Hap01
Netherlands: Rhine	Aas01_COI	KF888296			Aas01_16S	KF888279	Hap01
Netherlands: Rhine	Aas01_COI	KF888296			Aas01_16S	KF888279	Hap01
Netherlands: Rhine	Aas01_COI	KF888296			Aas01_16S	KF888279	Hap01
Netherlands: Rhine	Aas01_COI	KF888296			Aas01_16S	KF888279	Hap01
Netherlands: Rhine	Aas01_COI	KF888296			Aas01_16S	KF888279	Hap01
Netherlands: Rhine	Aas01_COI	KF888296			Aas01_16S	KF888279	Hap01
Netherlands: Rhine	Aas01_COI	KF888296			Aas01_16S	KF888279	Hap01
Netherlands: Rhine	Aas01_COI	KF888296			Aas01_16S	KF888279	Hap01
Poland: Bez Nazwu	Aas01_COI	KF888296			Aas01_16S	KF888279	Hap01

Poland: Bez Nazwu	Aas01_COI	KF888296	Aas01_16S	KF888279	Hap01
Poland: Bez Nazwu	Aas01_COI	KF888296	Aas01_16S	KF888279	Hap01
Poland: Bez Nazwu	Aas01_COI	KF888296	Aas01_16S	KF888279	Hap01
Poland: Bez Nazwu	Aas01_COI	KF888296	Aas01_16S	KF888279	Hap01
Poland: Czarne	Aas01_COI	KF888296	Aas01_16S	KF888279	Hap01
Poland: Czarne	Aas01_COI	KF888296	Aas01_16S	KF888279	Hap01
Poland: Czarne	Aas01_COI	KF888296	Aas01_16S	KF888279	Hap01
Poland: Czarne	Aas01_COI	KF888296	Aas01_16S	KF888279	Hap01
Poland: Czarne	Aas01_COI	KF888296	Aas01_16S	KF888279	Hap01
Poland: Lake 1 close to Miastko	Aas01_COI	KF888296	Aas01_16S	KF888279	Hap01
Poland: Lake 1 close to Miastko	Aas01_COI	KF888296	Aas01_16S	KF888279	Hap01
Poland: Lake 1 close to Miastko	Aas01_COI	KF888296	Aas01_16S	KF888279	Hap01
Poland: Lake 1 close to Miastko	Aas01_COI	KF888296	Aas01_16S	KF888279	Hap01
Poland: Lake 1 close to Miastko	Aas01_COI	KF888296	Aas01_16S	KF888279	Hap01
Poland: Lake 2 close to Miastko	Aas01_COI	KF888296	Aas01_16S	KF888279	Hap01
Poland: Lake 2 close to Miastko	Aas01_COI	KF888296	Aas01_16S	KF888279	Hap01
Poland: Lake 2 close to Miastko	Aas01_COI	KF888296	Aas01_16S	KF888279	Hap01
Poland: Lake 2 close to Miastko	Aas01_COI	KF888296	Aas01_16S	KF888279	Hap01
Poland: Lake 2 close to Miastko	Aas01_COI	KF888296	Aas01_16S	KF888279	Hap01
Poland: Lake 3 close to Miastko	Aas01_COI	KF888296	Aas01_16S	KF888279	Hap01
Poland: Lake 3 close to Miastko	Aas01_COI	KF888296	Aas01_16S	KF888279	Hap01
Poland: Lake 3 close to Miastko	Aas01_COI	KF888296	Aas01_16S	KF888279	Hap01
Poland: Lake 3 close to Miastko	Aas01_COI	KF888296	Aas01_16S	KF888279	Hap01
Poland: Lake 3 close to Miastko	Aas01_COI	KF888296	Aas01_16S	KF888279	Hap01
Poland: Rakowe Duze	Aas01_COI	KF888296	Aas01_16S	KF888279	Hap01
Poland: Rakowe Duze	Aas01_COI	KF888296	Aas01_16S	KF888279	Hap01
Poland: Rakowe Duze	Aas01_COI	KF888296	Aas01_16S	KF888279	Hap01
Poland: Rakowe Duze	Aas01_COI	KF888296	Aas01_16S	KF888279	Hap01
Poland: Rakowe Duze	Aas01_COI	KF888296	Aas01_16S	KF888279	Hap01
Poland: Rosko	Aas01_COI	KF888296	Aas01_16S	KF888279	Hap01
Poland: Rosko	Aas01_COI	KF888296	Aas01_16S	KF888279	Hap01
Poland: Rosko	Aas01_COI	KF888296	Aas05_16S	KF888283	Hap15
Poland: Rosko	Aas01_COI	KF888296	Aas05_16S	KF888283	Hap15
Poland: Rosko	Aas14_COI	KF888309	Aas01_16S	KF888279	Hap18
Poland: Seki	Aas01_COI	KF888296	Aas01_16S	KF888279	Hap01
Poland: Seki	Aas01_COI	KF888296	Aas01_16S	KF888279	Hap01
Poland: Seki	Aas01_COI	KF888296	Aas01_16S	KF888279	Hap01
Poland: Seki	Aas01_COI	KF888296	Aas01_16S	KF888279	Hap01
Poland: Seki	Aas01_COI	KF888296	Aas01_16S	KF888279	Hap01
Poland: Tomczyna	Aas01_COI	KF888296	Aas01_16S	KF888279	Hap01
Poland: Tomczyna	Aas01_COI	KF888296	Aas01_16S	KF888279	Hap01
Poland: Tomczyna	Aas01_COI	KF888296	Aas01_16S	KF888279	Hap01
Poland: Tomczyna	Aas01_COI	KF888296	Aas01_16S	KF888279	Hap01
Poland: Tomczyna	Aas01_COI	KF888296	Aas01_16S	KF888279	Hap01
Romania: Agrij	Aas01_COI	KF888296	Aas01_16S	KF888279	Hap01
Romania: Almasu Mic de Munte	Aas01_COI	JN254659			
Romania: Almasu Mic de Munte	Aas01_COI	JN254659			
Romania: Almasu Mic de Munte	AAS31_COI	JN254677			
Romania: Anina	Aas01_COI	JN254659			
Romania: Anina	Aas01_COI	JN254659			
Romania: Araneag	Aas01_COI	JN254659			
Romania: Araneag	Aas20_COI	JN254678			
Romania: Araneag	Aas16_COI	JN254669			
Romania: Bacaia	Aas01_COI	KF888296	Aas01_16S	KF888279	Hap01
Romania: Bacaia	Aas01_COI	KF888296	Aas01_16S	KF888279	Hap01

Romania: Baita	Aas01_COI	JN254659					
Romania: Baita	Aas01_COI	JN254659					
Romania: Balsa	Aas20_COI	KF888315			Aas13_16S	KF888291	Hap40
Romania: Balsa	Aas20_COI	KF888315			Aas13_16S	KF888291	Hap40
Romania: Balsa	Aas01_COI	KF888296			Aas01_16S	KF888279	Hap01
Romania: Balsa	Aas01_COI	KF888296			Aas01_16S	KF888279	Hap01
Romania: Balsa	Aas01_COI	KF888296			Aas01_16S	KF888279	Hap01
Romania: Balsa2	Aas20_COI	KF888315			Aas13_16S	KF888291	Hap40
Romania: Barcau	Aas01_COI	KF888296			Aas01_16S	KF888279	Hap01
Romania: Barcau	Aas20_COI	KF888315			Aas13_16S	KF888291	Hap40
Romania: Bezid	Aas01_COI		Lsh6	MW726612			
Romania: Bezid	Aas01_COI		Lsh6	MW726611			
Romania: Bezid	Aas01_COI		Lsh6	MW726610			
Romania: Bezid	Aas01_COI		Lsh6	MW726609			
Romania: Bezid	Aas01_COI		Lsh6	MW726608			
Romania: Bezid	Aas01_COI		Lsh6	MW726607			
Romania: Bezid	Aas01_COI		Lsh6	MW726606			
Romania: Bezid	Aas01_COI		Lsh6	MW726605			
Romania: Bichigiu	Aas01_COI	KF888296			Aas01_16S	KF888279	Hap01
Romania: Bogata	Aas17_COI	KF888312			Aas06_16S	KF888284	Hap24
Romania: Bradisoru de Jos	Aas16_COI	JN254669					
Romania: Buhui	Aas01_COI	KF888296			Aas01_16S	KF888279	Hap01
Romania: Buhui	Aas01_COI	KF888296			Aas01_16S	KF888279	Hap01
Romania: Caianu	Aas01_COI	KF888296			Aas01_16S	KF888279	Hap01
Romania: Caras	Aas16_COI	KF888311			Aas06_16S	KF888284	Hap23
Romania: Carasova	Aas16_COI	JN254669					
Romania: Carasova	Aas16_COI	JN254669					
Romania: Carnecea	Aas16_COI	JN254669					
Romania: Carpan	Aas23_COI	KF888318			Aas01_16S	KF888279	Hap34
Romania: Ciornovãt	Aas16_COI	KF888311			Aas06_16S	KF888284	Hap23
Romania: Ciornovãt	Aas16_COI	KF888311			Aas07_16S	KF888285	Hap22
Romania: Ciornovãt	Aas16_COI	KF888311			Aas07_16S	KF888285	Hap22
Romania: Ciornovãt	Aas16_COI	KF888311			Aas07_16S	KF888285	Hap22
Romania: Ciornovãt	Aas16_COI	KF888311			Aas07_16S	KF888285	Hap22
Romania: Ciornovãt	Aas16_COI	KF888311			Aas07_16S	KF888285	Hap22
Romania: Ciornovãt	Aas16_COI	KF888311			Aas07_16S	KF888285	Hap22
Romania: Cladovita	Aas01_COI	KF888296			Aas01_16S	KF888279	Hap01
Romania: Cladovita	Aas01_COI	KF888296			Aas01_16S	KF888279	Hap01
Romania: Cladovita	Aas08_COI	KF888303			Aas01_16S	KF888279	Hap10
Romania: Cladovita	Aas08_COI	KF888303			Aas01_16S	KF888279	Hap10
Romania: Clocotici	Aas24_COI	KF888319			Aas06_16S	KF888284	Hap36
Romania: Clocotici	Aas20_COI	KF888315			Aas06_16S	KF888284	Hap37
Romania: Clocotici	Aas20_COI	KF888315			Aas06_16S	KF888284	Hap37
Romania: Clocotici	Aas20_COI	KF888315			Aas06_16S	KF888284	Hap37
Romania: Clocotici	Aas20_COI	KF888315			Aas06_16S	KF888284	Hap37
Romania: Clocotici	Aas20_COI	KF888315			Aas06_16S	KF888284	Hap37
Romania: Clocotici	Aas20_COI	KF888315			Aas06_16S	KF888284	Hap37
Romania: Clocotici	Aas20_COI	KF888315			Aas06_16S	KF888284	Hap37
Romania: Comarnic	Aas16_COI	KF888311			Aas06_16S	KF888284	Hap23
Romania: Conop	Aas01_COI	KF888296			Aas01_16S	KF888279	Hap01
Romania: Conop	Aas20_COI	KF888315			Aas13_16S	KF888291	Hap40
Romania: Conop	Aas20_COI	KF888315			Aas13_16S	KF888291	Hap40
Romania: Crisul	Aas01_COI	KF888296			Aas01_16S	KF888279	Hap01
Romania: Crisul	Aas01_COI	KF888296			Aas01_16S	KF888279	Hap01
Romania: Crisul	Aas01_COI	KF888296			Aas01_16S	KF888279	Hap01

Romania: Derjana	Aas20_COI	KF888315			Aas13_16S	KF888291	Hap40
Romania: Dognecea	Aas01_COI	KF888296			Aas01_16S	KF888279	Hap01
Romania: Dognecea	Aas16_COI	KF888311			Aas06_16S	KF888284	Hap23
Romania: Dragoiestilor	Aas20_COI	KF888315			Aas13_16S	KF888291	Hap40
Romania: Dragoiestilor	Aas20_COI	KF888315			Aas13_16S	KF888291	Hap40
Romania: Dragoiestilor	Aas20_COI	KF888315			Aas13_16S	KF888291	Hap40
Romania: Dragoiestilor	Aas20_COI	KF888315			Aas13_16S	KF888291	Hap40
Romania: Dragoiestilor	Aas20_COI	KF888315			Aas13_16S	KF888291	Hap40
Romania: Forotic	Aas16_COI	JN254669					
Romania: Forotic	Aas16_COI	JN254669					
Romania: Forotic	Aas16_COI	JN254669					
Romania: Forotic	Aas16_COI	JN254669					
Romania: Forotic	Aas16_COI	JN254669					
Romania: Forotic	Aas16_COI	JN254669					
Romania: Galben	Aas20_COI	KF888315			Aas11_16S	KF888289	Hap33
Romania: Garliste	Aas16_COI	JN254669					
Romania: Garliste	Aas16_COI	JN254669					
Romania: Garliste	Aas16_COI	JN254669					
Romania: Garliste	Aas16_COI	JN254669					
Romania: Garliste	Aas16_COI	JN254669					
Romania: Geoagiu	Aas01_COI	KF888296			Aas01_16S	KF888279	Hap01
Romania: Giacas	Aas01_COI	KF888296			Aas01_16S	KF888279	Hap01
Romania: Giacas	Aas01_COI	KF888296			Aas01_16S	KF888279	Hap01
Romania: Hartagani	Aas23_COI	JN254679					
Romania: Hartibaciu	Aas16_COI	KF888311			Aas06_16S	KF888284	Hap23
Romania: Hartibaciu	Aas16_COI	KF888311			Aas06_16S	KF888284	Hap23
Romania: Hartibaciu	Aas16_COI	KF888311			Aas06_16S	KF888284	Hap23
Romania: Iazul	Aas16_COI	KF888311			Aas06_16S	KF888284	Hap23
Romania: Maierus	Aas17_COI	KF888312			Aas06_16S	KF888284	Hap24
Romania: Moravita	Aas01_COI	KF888296			Aas01_16S	KF888279	Hap01
Romania: Moravita	Aas01_COI	KF888296			Aas06_16S	KF888284	Hap19
Romania: Moravita	Aas16_COI	KF888311			Aas06_16S	KF888284	Hap23
Romania: Moravita	Aas16_COI	KF888311			Aas06_16S	KF888284	Hap23
Romania: Moravita	Aas16_COI	KF888311			Aas06_16S	KF888284	Hap23
Romania: Nadas	Aas01_COI	KF888296			Aas01_16S	KF888279	Hap01
Romania: Nadas	Aas01_COI	KF888296			Aas01_16S	KF888279	Hap01
Romania: Nadas	Aas01_COI	KF888296			Aas01_16S	KF888279	Hap01
Romania: Nadas	Aas01_COI	KF888296			Aas01_16S	KF888279	Hap01
Romania: Nadas	Aas01_COI	KF888296			Aas01_16S	KF888279	Hap01
Romania: Nadas	Aas01_COI	KF888296			Aas01_16S	KF888279	Hap01
Romania: Nadas	Aas01_COI	KF888296			Aas01_16S	KF888279	Hap01
Romania: Nadas	Aas01_COI	KF888296			Aas01_16S	KF888279	Hap01
Romania: Nadas	Aas24_COI	KF888319			Aas01_16S	KF888279	Hap35
Romania: Nadas	Aas20_COI	KF888315			Aas12_16S	KF888290	Hap38
Romania: Nadas	Aas20_COI	KF888315			Aas12_16S	KF888290	Hap38
Romania: Nadas	Aas20_COI	KF888315			Aas12_16S	KF888290	Hap38
Romania: Natra	Aas16_COI	KF888311			Aas06_16S	KF888284	Hap23
Romania: Niraj	Aas01_COI	KF888296			Aas01_16S	KF888279	Hap01
Romania: Ocna de Fier	Aas01_COI	JN254659					
Romania: Ocna de Fier	Aas16_COI	JN254669					
Romania: Ocna de Fier	Aas16_COI	JN254669					
Romania: Ocna de Fier	Aas16_COI	JN254669					
Romania: Oituz	Aas16_COI	KF888311			Aas06_16S	KF888284	Hap23
Romania: Petresti (Sebes)	Aas36_COI		Lsh8	MW726532			
Romania: Petresti (Sebes)	Aas36_COI		Lsh8	MW726531			

Romania: Petresti (Sebes)	Aas36_COI		Lsh8	MW726530			
Romania: Petresti (Sebes)	Aas36_COI		Lsh8	MW726529			
Romania: Petresti (Sebes)	Aas36_COI		Lsh8	MW726528			
Romania: Petresti (Sebes)	Aas36_COI		Lsh8	MW726527			
Romania: Petresti (Sebes)	Aas36_COI		Lsh8	MW726526			
Romania: Poiana	Aas01_COI	KF888296			Aas01_16S	KF888279	Hap01
Romania: Racas	Aas20_COI	KF888315			Aas13_16S	KF888291	Hap40
Romania: Ravistea	Aas16_COI	KF888311			Aas06_16S	KF888284	Hap23
Romania: Ravistea	Aas16_COI	KF888311			Aas06_16S	KF888284	Hap23
Romania: Ravistea	Aas16_COI	KF888311			Aas06_16S	KF888284	Hap23
Romania: Ravistea	Aas16_COI	KF888311			Aas06_16S	KF888284	Hap23
Romania: Ravistea	Aas16_COI	KF888311			Aas06_16S	KF888284	Hap23
Romania: Ravistea	Aas16_COI	KF888311			Aas06_16S	KF888284	Hap23
Romania: Ravistea	Aas16_COI	KF888311			Aas06_16S	KF888284	Hap23
Romania: Ravistea	Aas16_COI	KF888311			Aas06_16S	KF888284	Hap23
Romania: Rupea	Aas16_COI	KF888311			Aas06_16S	KF888284	Hap23
Romania: Rupea	Aas16_COI	KF888311			Aas06_16S	KF888284	Hap23
Romania: Rupea	Aas16_COI	KF888311			Aas06_16S	KF888284	Hap23
Romania: Rupea	Aas16_COI	KF888311			Aas06_16S	KF888284	Hap23
Romania: Sarasau	Aas01_COI	KF888296			Aas01_16S	KF888279	Hap01
Romania: Schiopu	Aas01_COI	KF888296			Aas01_16S	KF888279	Hap01
Romania: Simbrezi	Aas16_COI	KF888311			Aas06_16S	KF888284	Hap23
Romania: Simbrezi	Aas16_COI	KF888311			Aas06_16S	KF888284	Hap23
Romania: Sinca	Aas16_COI	KF888311			Aas06_16S	KF888284	Hap23
Romania: Sohodol	Aas20_COI	KF888315			Aas13_16S	KF888291	Hap40
Romania: Solocma	Aas01_COI	KF888296			Aas01_16S	KF888279	Hap01
Romania: Solocma	Aas01_COI	KF888296			Aas01_16S	KF888279	Hap01
Romania: Solocma	Aas01_COI	KF888296			Aas01_16S	KF888279	Hap01
Romania: Somesul Rece	Aas01_COI		Lsh9	MW726514			
Romania: Somesul Rece	Aas01_COI		Lsh6	MW726513			
Romania: Somesul Rece	Aas01_COI		Lsh6	MW726512			
Romania: Somesul Rece	Aas01_COI		Lsh6	MW726511			
Romania: Somesul Rece	Aas01_COI		Lsh6	MW726510			
Romania: Somesul Rece	Aas01_COI		Lsh6	MW726509			
Romania: Somesul Rece	Aas01_COI		Lsh6	MW726508			
Romania: Somesul Rece	Aas01_COI		Lsh6	MW726507			
Romania: Somesul Rece	Aas01_COI		Lsh6	MW726506			
Romania: Somesul Rece	Aas01_COI		Lsh6	MW726505			
Romania: Somesul Rece	Aas01_COI		Lsh6	MW726504			
Romania: Somesul Rece	Aas01_COI		Lsh6	MW726503			
Romania: Somesul Rece	Aas01_COI		Lsh6	MW726502			
Romania: Somesul Rece	Aas01_COI		Lsh6	MW726501			
Romania: Somesul Rece	Aas01_COI		Lsh6	MW726500			
Romania: Stramba	Aas20_COI	KF888315			Aas13_16S	KF888291	Hap40
Romania: Tamasesti	Aas01_COI	KF888296			Aas01_16S	KF888279	Hap01
Romania: Tamasesti	Aas01_COI	KF888296			Aas01_16S	KF888279	Hap01
Romania: Tamasesti	Aas01_COI	KF888296			Aas01_16S	KF888279	Hap01
Romania: Tamasesti	Aas01_COI	KF888296			Aas01_16S	KF888279	Hap01
Romania: Taraia	Aas16_COI	KF888311			Aas06_16S	KF888284	Hap23
Romania: Tebea	Aas20_COI	KF888315			Aas13_16S	KF888291	Hap40
Romania: Tetisu	Aas01_COI	KF888296			Aas01_16S	KF888279	Hap01
Romania: Toplita	Aas16_COI	KF888311			Aas06_16S	KF888284	Hap23
Romania: Valea Adanca	Aas01_COI	KF888296			Aas01_16S	KF888279	Hap01
Romania: Valea Adanca	Aas15_COI	KF888310			Aas06_16S	KF888284	Hap20
Romania: Valea Boului	Aas01_COI	KF888296			Aas01_16S	KF888279	Hap01

Romania: Valea Crisului	Aas01_COI	KF888296			Aas01_16S	KF888279	Hap01
Romania: Valea Crisului	Aas01_COI	KF888296			Aas01_16S	KF888279	Hap01
Romania: Valea Crisului	Aas01_COI	KF888296			Aas01_16S	KF888279	Hap01
Romania: Valea Crisului	Aas01_COI	KF888296			Aas01_16S	KF888279	Hap01
Romania: Valea Holita	Aas01_COI	KF888296			Aas01_16S	KF888279	Hap01
Romania: Valea Mare	Aas01_COI	KF888296			Aas01_16S	KF888279	Hap01
Romania: Valea Pestilor	Aas25_COI	KF888320			Aas01_16S	KF888279	Hap39
Romania: Valea Stoiaca	Aas01_COI	KF888296			Aas01_16S	KF888279	Hap01
Romania: Valea Stoiaca	Aas20_COI	KF888315			Aas13_16S	KF888291	Hap40
Romania: Valea Tejei	Aas01_COI	KF888296			Aas01_16S	KF888279	Hap01
Romania: Venetia	Aas16_COI	KF888311			Aas06_16S	KF888284	Hap23
Serbia: Buser Lake, Despotovac	Aas16_COI		Lsh3	MW726582	Aas09_16S	MW726306	Hap26
Serbia: Buser Lake, Despotovac	Aas16_COI		Lsh3	MW726581	Aas09_16S	MW726305	Hap26
Serbia: Buser Lake, Despotovac	Aas16_COI		Lsh3	MW726580	Aas09_16S	MW726304	Hap26
Serbia: Gazivode Lake	Aas16_COI		Lsh3	MW726578	Aas09_16S	MW726302	Hap26
Serbia: Gazivode Lake	Aas16_COI		Lsh3	MW726577	Aas09_16S	MW726301	Hap26
Serbia: Gazivode Lake	Aas16_COI		Lsh3	MW726576	Aas09_16S	MW726300	Hap26
Serbia: Gazivode Lake	Aas16_COI		Lsh3	MW726575	Aas09_16S	MW726299	Hap26
Serbia: Gazivode Lake	Aas16_COI		Lsh3	MW726574	Aas09_16S	MW726298	Hap26
Serbia: Gazivode Lake	Aas16_COI		Lsh3	MW726573	Aas09_16S	MW726297	Hap26
Serbia: Gazivode Lake	Aas16_COI		Lsh3	MW726572	Aas09_16S	MW726296	Hap26
Serbia: Gazivode Lake	Aas16_COI		Lsh3	MW726571	Aas09_16S	MW726295	Hap26
Serbia: Gazivode Lake	Aas16_COI		Lsh3	MW726570	Aas09_16S	MW726294	Hap26
Serbia: Gazivode Lake	Aas16_COI		Lsh3	MW726569	Aas09_16S	MW726293	Hap26
Serbia: Gazivode Lake	Aas16_COI		Lsh3	MW726568			
Serbia: Grliško Lake, Zaječar	Aas16_COI		Lsh3	MW726604	Aas09_16S	MW726315	Hap26
Serbia: Grliško Lake, Zaječar	Aas16_COI		Lsh3	MW726603	Aas09_16S	MW726314	Hap26
Serbia: Grliško Lake, Zaječar	Aas16_COI		Lsh3	MW726602	Aas09_16S	MW726313	Hap26
Serbia: Grliško Lake, Zaječar	Aas16_COI		Lsh3	MW726601	Aas09_16S	MW726312	Hap26
Serbia: Grliško Lake, Zaječar	Aas16_COI		Lsh3	MW726600	Aas09_16S	MW726311	Hap26
Serbia: Grliško Lake, Zaječar	Aas16_COI		Lsh3	MW726599	Aas09_16S	MW726310	Hap26
Serbia: Grliško Lake, Zaječar	Aas16_COI		Lsh3	MW726598	Aas09_16S	MW726309	Hap26
Serbia: Grliško Lake, Zaječar	Aas16_COI		Lsh3	MW726597	Aas09_16S	MW726308	Hap26
Serbia: Grliško Lake, Zaječar	Aas16_COI		Lsh3	MW726596	Aas09_16S	MW726307	Hap26
Serbia: Grliško Lake, Zaječar	Aas16_COI		Lsh3	MW726595			
Serbia: Grliško Lake, Zaječar	Aas16_COI		Lsh3	MW726594			
Serbia: Grliško Lake, Zaječar	Aas16_COI		Lsh3	MW726593			
Serbia: Gruža Lake, Knič	Aas16_COI		Lsh3	MW726579	Aas09_16S	MW726303	Hap26
Serbia: Kačer River, Ljig	Aas33_COI		Lsh5	MW726614	Aas10_16S	MW726317	Hap49
Serbia: Kačer River, Ljig	Aas33_COI		Lsh5	MW726613	Aas10_16S	MW726316	Hap49
Serbia: Kačer River, Ljig	Aas33_COI		Lsh5	MW726625	Aas10_16S	MW726326	Hap49
Serbia: Kačer River, Ljig	Aas33_COI		Lsh5	MW726624	Aas10_16S	MW726325	Hap49
Serbia: Kačer River, Ljig	Aas33_COI		Lsh5	MW726623	Aas10_16S	MW726324	Hap49
Serbia: Kačer River, Ljig	Aas33_COI		Lsh5	MW726622	Aas10_16S	MW726323	Hap49
Serbia: Kačer River, Ljig	Aas33_COI		Lsh5	MW726621	Aas10_16S	MW726322	Hap49
Serbia: Kačer River, Ljig	Aas33_COI		Lsh5	MW726620	Aas10_16S	MW726321	Hap49
Serbia: Kačer River, Ljig	Aas33_COI		Lsh5	MW726619	Aas10_16S	MW726320	Hap49
Serbia: Kačer River, Ljig	Aas33_COI		Lsh5	MW726618	Aas10_16S	MW726319	Hap49
Serbia: Kačer River, Ljig	Aas33_COI		Lsh5	MW726617	Aas10_16S	MW726318	Hap49
Serbia: Kačer River, Ljig	Aas33_COI		Lsh5	MW726616			
Serbia: Kačer River, Ljig	Aas33_COI		Lsh5	MW726615			
Serbia: Korenica Lake, Despotovac	Aas16_COI		Lsh3	MW726567	Aas09_16S	MW726292	Hap26
Serbia: Korenica Lake, Despotovac	Aas16_COI		Lsh3	MW726566	Aas09_16S	MW726291	Hap26
Serbia: Korenica Lake, Despotovac	Aas16_COI		Lsh3	MW726565	Aas09_16S	MW726290	Hap26
Serbia: Korenica Lake, Despotovac	Aas16_COI		Lsh3	MW726564	Aas09_16S	MW726289	Hap26

Serbia: Korenica Lake, Despotovac	Aas16_COI	Lsh3	MW726563	Aas09_16S	MW726288	Hap26
Serbia: Korenica Lake, Despotovac	Aas16_COI	Lsh3	MW726562	Aas09_16S	MW726287	Hap26
Serbia: Korenica Lake, Despotovac	Aas16_COI	Lsh3	MW726561	Aas09_16S	MW726286	Hap26
Serbia: Korenica Lake, Despotovac	Aas16_COI	Lsh3	MW726560	Aas09_16S	MW726285	Hap26
Serbia: Korenica Lake, Despotovac	Aas16_COI	Lsh3	MW726559	Aas09_16S	MW726284	Hap26
Serbia: Korenica Lake, Despotovac	Aas16_COI	Lsh3	MW726558	Aas09_16S	MW726283	Hap26
Serbia: Lepenica River, Kragujevac	Aas16_COI	Lsh3	MW726515	AAS18_16S	MW726259	Hap52
Serbia: Milošev ribnjak, Kragujevac	Aas16_COI	Lsh3	MW726557	Aas09_16S	MW726282	Hap26
Serbia: Milošev ribnjak, Kragujevac	Aas16_COI	Lsh3	MW726556	Aas09_16S	MW726281	Hap26
Serbia: Milošev ribnjak, Kragujevac	Aas16_COI	Lsh3	MW726555	Aas09_16S	MW726280	Hap26
Serbia: Milošev ribnjak, Kragujevac	Aas16_COI	Lsh3	MW726554	Aas09_16S	MW726279	Hap26
Serbia: Milošev ribnjak, Kragujevac	Aas16_COI	Lsh3	MW726553	Aas09_16S	MW726278	Hap26
Serbia: Milošev ribnjak, Kragujevac	Aas16_COI	Lsh3	MW726552	Aas09_16S	MW726277	Hap26
Serbia: Petrovačka River, Kragujevac	Aas16_COI	Lsh3	MW726545			
Serbia: Petrovačka River, Kragujevac	Aas16_COI	Lsh3	MW726544	Aas09_16S	MW726276	Hap26
Serbia: Petrovačka River, Kragujevac	Aas16_COI	Lsh3	MW726543	Aas09_16S	MW726275	Hap26
Serbia: Petrovačka River, Kragujevac	Aas16_COI	Lsh3	MW726542	Aas09_16S	MW726274	Hap26
Serbia: Petrovačka River, Kragujevac	Aas16_COI	Lsh3	MW726541	Aas09_16S	MW726273	Hap26
Serbia: Petrovačka River, Kragujevac	Aas16_COI	Lsh3	MW726540	Aas09_16S	MW726272	Hap26
Serbia: Petrovačka River, Kragujevac	Aas16_COI	Lsh3	MW726539	Aas09_16S	MW726271	Hap26
Serbia: Petrovačka River, Kragujevac	Aas16_COI	Lsh3	MW726538	Aas09_16S	MW726270	Hap26
Serbia: Petrovačka River, Kragujevac	Aas16_COI	Lsh3	MW726537	Aas09_16S	MW726269	Hap26
Serbia: Petrovačka River, Kragujevac	Aas32_COI	Lsh7	MW726536	Aas09_16S	MW726268	Hap53
Serbia: Petrovačka River, Kragujevac	Aas16_COI	Lsh3	MW726535	Aas09_16S	MW726267	Hap26
Serbia: Petrovačka River, Kragujevac	Aas16_COI	Lsh3	MW726534	Aas09_16S	MW726266	Hap26
Serbia: Petrovačka River, Kragujevac	Aas16_COI	Lsh3	MW726533	Aas09_16S	MW726265	Hap26
Serbia: Resnički Stream, Kragujevac	Aas16_COI	Lsh3	MW726525	Aas09_16S	MW726264	Hap26
Serbia: Resnički Stream, Kragujevac	Aas16_COI	Lsh3	MW726524			
Serbia: Resnički Stream, Kragujevac	Aas16_COI	Lsh3	MW726523	Aas09_16S	MW726263	Hap26
Serbia: Resnički Stream, Kragujevac	Aas16_COI	Lsh3	MW726522	Aas09_16S	MW726262	Hap26
Serbia: Resnički Stream, Kragujevac	Aas16_COI	Lsh3	MW726521	Aas09_16S	MW726261	Hap26
Serbia: Resnički Stream, Kragujevac	Aas16_COI	Lsh3	MW726520			
Serbia: Resnički Stream, Kragujevac	Aas16_COI	Lsh3	MW726519			
Serbia: Resnički Stream, Kragujevac	Aas16_COI	Lsh3	MW726518	Aas09_16S	MW726260	Hap26
Serbia: Resnički Stream, Kragujevac	Aas16_COI	Lsh3	MW726517			
Serbia: Resnički Stream, Kragujevac	Aas16_COI	Lsh3	MW726516			
Slovenia: Bloščica				AAS17_16S	MZ467199	
Slovenia: Bloščica				AAS17_16S	MZ467200	
Slovenia: Čolnišček				AAS17_16S	MZ467218	
Slovenia: Kočevska reka	Aas35_COI	Lsh18	MW726413	AAS17_16S	MW726217	Hap51
Slovenia: Kočevska reka	Aas35_COI	Lsh18	MW726412	AAS17_16S	MW726216	Hap51
Slovenia: Kočevska reka	Aas35_COI	Lsh18	MW726411	AAS17_16S	MW726215	Hap51
Slovenia: Kočevska reka	Aas35_COI	Lsh18	MW726410	AAS17_16S	MW726214	Hap51
Slovenia: Kočevska reka	Aas35_COI	Lsh18	MW726409	AAS17_16S	MW726213	Hap51
Slovenia: Kočevska reka	Aas35_COI	Lsh18	MW726408	AAS17_16S	MW726212	Hap51
Slovenia: Kočevska reka	Aas35_COI	Lsh18	MW726407	AAS17_16S	MW726211	Hap51
Slovenia: Lake Bled	Aas35_COI	Lsh18	MW726427	AAS17_16S	MW726230	Hap51
Slovenia: Lake Bled	Aas35_COI	Lsh18	MW726426	AAS17_16S	MW726229	Hap51
Slovenia: Lake Bled	Aas35_COI	Lsh18	MW726425	AAS17_16S	MW726228	Hap51
Slovenia: Lake Bloke	Aas35_COI	Lsh18	MW726424	AAS17_16S	MW726227	Hap51
Slovenia: Lake Bloke	Aas35_COI	Lsh18	MW726423	AAS17_16S	MW726226	Hap51
Slovenia: Lake Bloke	Aas35_COI	Lsh18	MW726422	AAS17_16S	MW726225	Hap51
Slovenia: Lake Bloke	Aas35_COI	Lsh18	MW726421	AAS17_16S	MW726224	Hap51
Slovenia: Lake Bloke	Aas35_COI	Lsh18	MW726420	AAS17_16S	MW726223	Hap51
Slovenia: Lake Bloke	Aas35_COI	Lsh18	MW726419	AAS17_16S	MW726222	Hap51

Slovenia: Lake Bloke	Aas35_COI	Lsh18	MW726418	AAS17_16S	MW726221	Hap51
Slovenia: Lake Bloke	Aas35_COI	Lsh18	MW726417	AAS17_16S	MW726220	Hap51
Slovenia: Lake Bloke	Aas35_COI	Lsh18	MW726416	AAS17_16S	MW726219	Hap51
Slovenia: Lake Bloke	Aas35_COI	Lsh18	MW726415	AAS17_16S	MW726218	Hap51
Slovenia: Lake Bloke	Aas35_COI	Lsh18	MW726414			
Slovenia: pritoka Bloščice, Mlaka	AAS35_COI	Lsh18	MZ456432	AAS17_16S	MZ467221	Hap51
Slovenia: Rakiški graben, Rakitna	AAS35_COI	Lsh18	MZ456433	AAS17_16S	MZ467222	Hap51
Slovenia: Rakiški graben, Rakitna				AAS17_16S	MZ467223	
Slovenia: Šmihelski stream	AAS35_COI	Lsh18	MZ456431	AAS17_16S	MZ467220	Hap51
Slovenia: Vogršček	Aas27_COI	Lsh14	MZ456430	AAS15_16S	MZ467219	Hap43
