# Supplementary material for: Integrating population genetics and species distribution modelling to guide conservation of the noble crayfish, Astacus astacus, in Croatia
Source: Sci Rep. 2022 Feb 7;12:2040. doi: 10.1038/s41598-022-06027-8 (PMC8821615; doi:10.1038/s41598-022-06027-8)
Supplement: Supplementary file 2 — Supplementary Information. [file 41598_2022_6027_MOESM2_ESM.docx]

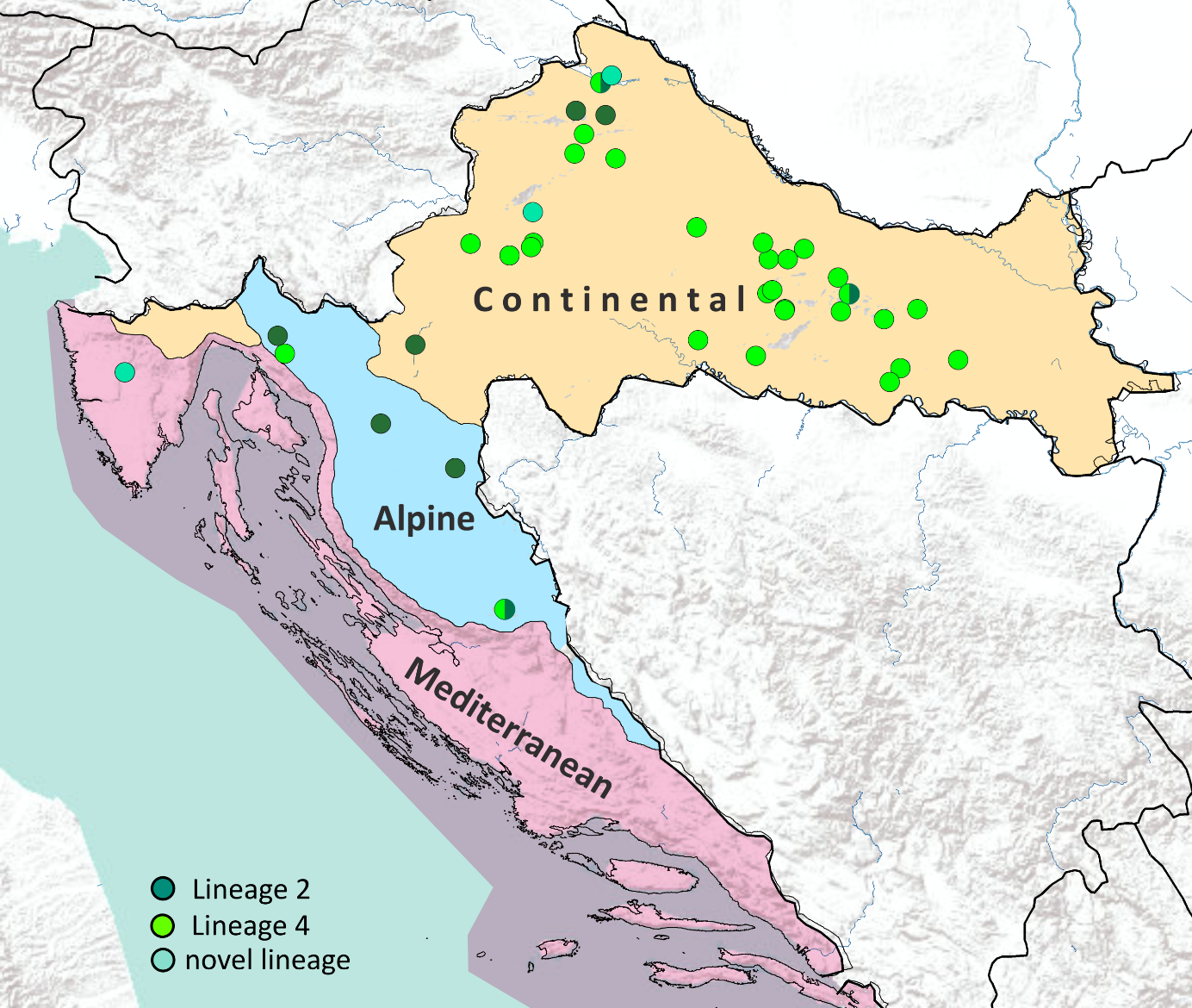


**Supplementary Figure** **S1.**

Geographical distribution of mtDNA lineages of *Astacus astacus* in Croatia with indication of three biogeographical regions (Alpine, Continental and Mediterranean). Map was produced in ArcGIS 10.3 program package and finished in the program Inscape 1.0 by authors of this study.

.


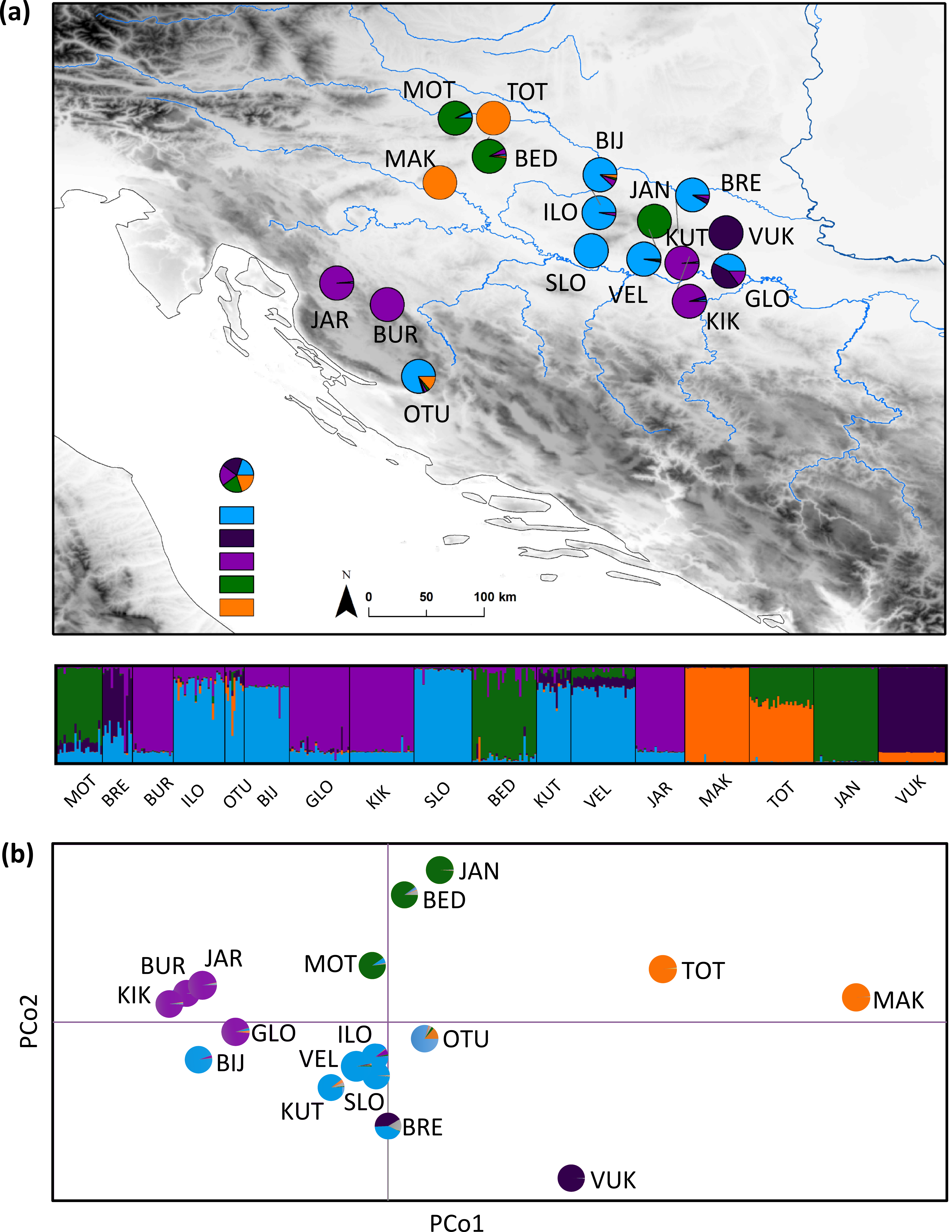


**Supplementary Figure** **S2.**

Genetic structure of the 17 studied *Astacus astacus* populations (see Supplementary Table S2 for abbreviation) based on 15 microsatellites. (a) Genetic clustering inferred by STRUCTURE with the suggested K = 5 clusters. (b) Plots of the first two axes of a principal coordinates analysis (PCoA) based on Nei’ DA genetic distances. Each dot represents one population with colours depicting genetic cluster identified in STUCTURE. Grey shading in the map indicates altitude in the study area. Map was produced in ArcGIS 10.3 program package by authors of this study.


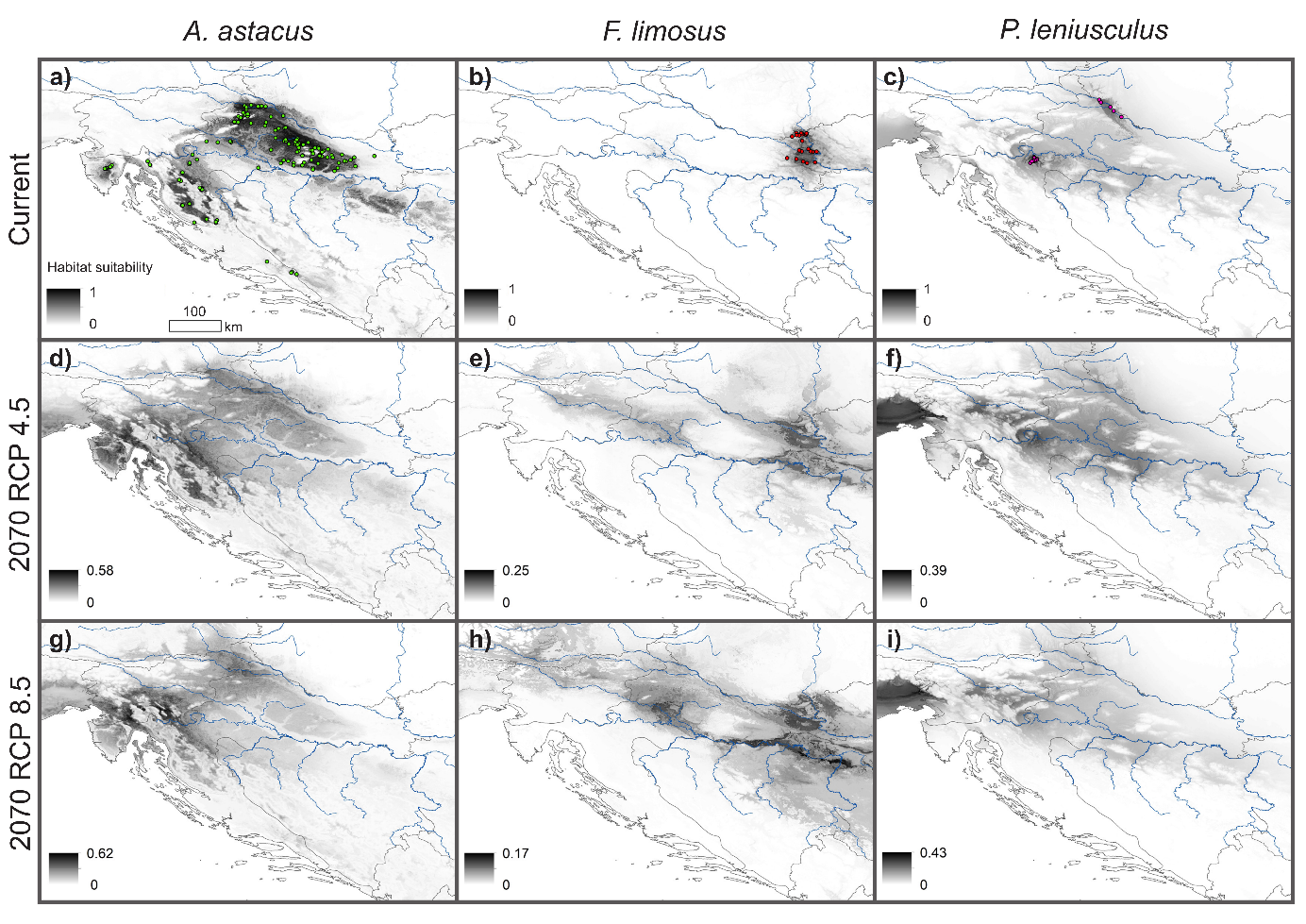


**Supplementary Figure** **S3.**

Ensemble potential habitat suitability for indigenous *Astacus astacus* and the two non- indigenous species, *Pacifastacus leniusculus* and *Faxonius limosus* in Croatia under current conditions (a-c), future RCP 4.5 scenario (d-f), and future RCP 8.5 scenario (g-f) in 2070 based on SDMs. Note that habitat suitability values in current projections are on the scale from 0 (unsuitable) to 1 (high suitability), while in the future projections habitat suitability values are on the scale from 0 (unsuitable) to maximum projected habitat suitability value. Maps were produced in ArcGIS 10.3 program package by authors of this study.

**Supplementary Table** **S2.** Probability (bold indicates significant p-values; p < 0.05) of bottleneck for *Astacus astacus* population using Wilcoxon sign rank test under three different mutational models: infinite allele model (IAM), stepwise mutation model (SMM) and two-phase model (TPM).

|  |  | **Wilcoxon sign-rank test 1-tail** | | | **Wilcoxon sign-rank test 2-tail** | | |
| --- | --- | --- | --- | --- | --- | --- | --- |
| **Population** | **Pop. Ab.** | **IAM** | **TPM** | **SMM** | **IAM** | **TPM** | **SMM** |
| Motičnjak | MOT | **0.00** | **0.02** | 0.47 | **0.00** | **0.04** | 0.93 |
| Breznica | BRE | **0.03** | 0.19 | 0.82 | 0.06 | 0.39 | 0.39 |
| Burgeti | BUR | 0.26 | 0.72 | 0.96 | 0.52 | 0.60 | 0.09 |
| Ilova | ILO | **0.00** | **0.02** | 0.53 | **0.00** | **0.04** | 0.98 |
| Otuča | OTU | **0.01** | 0.10 | 0.40 | **0.02** | 0.21 | 0.80 |
| Bijela | BIJ | **0.04** | 0.26 | 0.68 | 0.08 | 0.52 | 0.68 |
| Glogovica | GLO | **0.01** | 0.49 | 0.99 | **0.01** | 0.98 | **0.03** |
| Kikovac | KIK | **0.01** | 0.08 | 0.64 | **0.02** | 0.17 | 0.76 |
| Sloboština | SLO | **0.00** | **0.02** | 0.48 | **0.00** | **0.04** | 0.95 |
| Bednja | BED | 0.08 | 0.85 | 0.99 | 0.17 | 0.33 | **0.03** |
| Kutjevačka | KUT | **0.00** | 0.08 | 0.83 | **0.00** | 0.17 | 0.36 |
| Veličanka | VEL | 0.05 | 0.40 | 0.96 | 0.09 | 0.80 | 0.08 |
| Jaruga | JAR | **0.00** | **0.00** | **0.00** | **0.00** | **0.00** | **0.00** |
| Maksimir | MAK | 0.27 | 0.62 | 0.85 | 0.54 | 0.81 | 0.33 |
| Totovec | TOT | **0.00** | **0.00** | **0.00** | **0.00** | **0.00** | **0.00** |
| Jankovec | JAN | **0.02** | 0.15 | 0.70 | **0.04** | 0.30 | 0.64 |
| Vuka | VUK | **0.00** | **0.03** | 0.17 | **0.00** | 0.06 | 0.34 |

**Supplementary Table** **S3.** Analysis of Molecular Variance (AMOVA) within and among populations of *Astacus astacus* (level of significance is based on 10,000 iterations). Populations were grouped based on their affiliation to genetic clusters obtained by STRUCTURE analysis.

|  | **Sum of squares** | **Variance components** | **Percentage of variation** |
| --- | --- | --- | --- |
| Among groups | 232.76 | 0.39 | 6.31 |
| Among populations within groups | 1262.36 | 1.68 | 27.01 |
| Within populations | 333.755 | 4.16 | 66.67 |

**Supplementary Table** **S4.** Mean AUC across 10 replicate runs and standard deviation values for each modelling method used for building species distribution models (SDMs) of *Astacus astacus* and the two invasive non-indigenous crayfish species (*Faxonius limosus* and *Pacifastacus leniusculus*).

|  | ***Astacus astacus*** | ***Faxonius limosus*** | ***Pacifastacus leniusculus*** |
| --- | --- | --- | --- |
| **RF** | 0.99 (± 0.006) | 0.99 (± 0.001) | 0.99 (± 0.032) |
| **GBM** | 0.98 (± 0.004) | 0.98 (± 0.037) | 0.98 (± 0.045) |
| **MAXENT** | 0.97 (± 0.019) | 0.99 (± 0.001) | 0.99 (± 0.009) |
